# Identification of gene networks mediating regional resistance to tauopathy in late-onset Alzheimer’s disease

**DOI:** 10.1371/journal.pgen.1010681

**Published:** 2023-03-27

**Authors:** Christopher A. Ayoub, Connor S. Wagner, Jeff Kuret

**Affiliations:** 1 Biomedical Sciences Graduate Program, Ohio State University, Columbus, Ohio, United States of America; 2 Medical Scientist Training Program, Ohio State University, Columbus, Ohio, United States of America; 3 Department of Biological Chemistry & Pharmacology, Ohio State University, Columbus, Ohio, United States of America; The Jackson Laboratory, UNITED STATES

## Abstract

Neurofibrillary lesions composed of tau protein aggregates are defining hallmarks of Alzheimer’s Disease. Despite tau filaments appearing to spread between networked brain regions in a prion-like manner, certain areas including cerebellum resist trans-synaptic spread of tauopathy and degeneration of their constituent neuronal cell bodies. To identify molecular correlates of resistance, we derived and implemented a ratio of ratios approach for disaggregating gene expression data on the basis of regional vulnerability to tauopathic neurodegeneration. When applied to vulnerable pre-frontal cortex as an internal reference for resistant cerebellum, the approach segregated adaptive changes in expression into two components. The first was enriched for neuron-derived transcripts associated with proteostasis including specific members of the molecular chaperone family and was unique to resistant cerebellum. When produced as purified proteins, each of the identified chaperones depressed aggregation of 2N4R tau in vitro at sub-stoichiometric concentrations, consistent with the expression polarity deduced from ratio of ratios testing. In contrast, the second component enriched for glia- and microglia-derived transcripts associated with neuroinflammation, segregating these pathways from susceptibility to tauopathy. These data support the utility of ratio of ratios testing for establishing the polarity of gene expression changes with respect to selective vulnerability. The approach has the potential to identify new targets for drug discovery predicated on their ability to promote resistance to disease in vulnerable neuron populations.

## Author summary

Alzheimer’s disease (AD) is defined in part by the appearance of intraneuronal aggregates composed of tau protein. Because their spread through the brain correlates with cognitive decline and neurodegeneration, tau lesions serve as markers for staging disease and for recognizing nerve cell populations differentially vulnerable or resistant to AD pathogenesis. Here we compared a resistant to a vulnerable region of human brain to identify associated molecular signatures and their relationship to risk factors deduced through large-scale human genetics studies. We found that expression of genes associated with protection against intracellular protein misfolding and aggregation accompanied resistance, and that the magnitude of change correlated directly with tau burden appearing in connected brain regions. Resistance also was accompanied by expression changes in two validated AD risk genes that reproduced the directionality reported through genetic analysis. These data support the use of selective vulnerability for interrogating the directionality of gene expression changes in AD and for identifying candidate mediators of resistance to tau pathology.

## Introduction

Late-onset Alzheimer’s disease (LOAD) is a high-prevalence, primarily sporadic disorder defined biologically by neurodegeneration and the appearance of intra- and extracellular lesions composed of tau and Aβ, respectively [1]. Tau lesions in particular are sensitive biomarkers of disease because their appearance correlates with neuronal loss [2–4] and functional cognitive deficits [5]. Tau lesions first appear in the locus coeruleus of brainstem, then march hierarchically toward association (*e*.*g*., pre-frontal cortex) and finally primary neocortical areas [6,7]. The spatiotemporal pattern of increasing pathological tau burden has been codified by the Braak staging system and rationalized by a prion-like mechanism of cell-to-cell spread. Specifically, the prion-like hypothesis posits that the propagation of misfolded tau protein across synapses within a connectivity network is responsible for templating tau lesion formation in naïve cell populations, forming toxic aggregate conformers [8–10].

**Fig 1:**
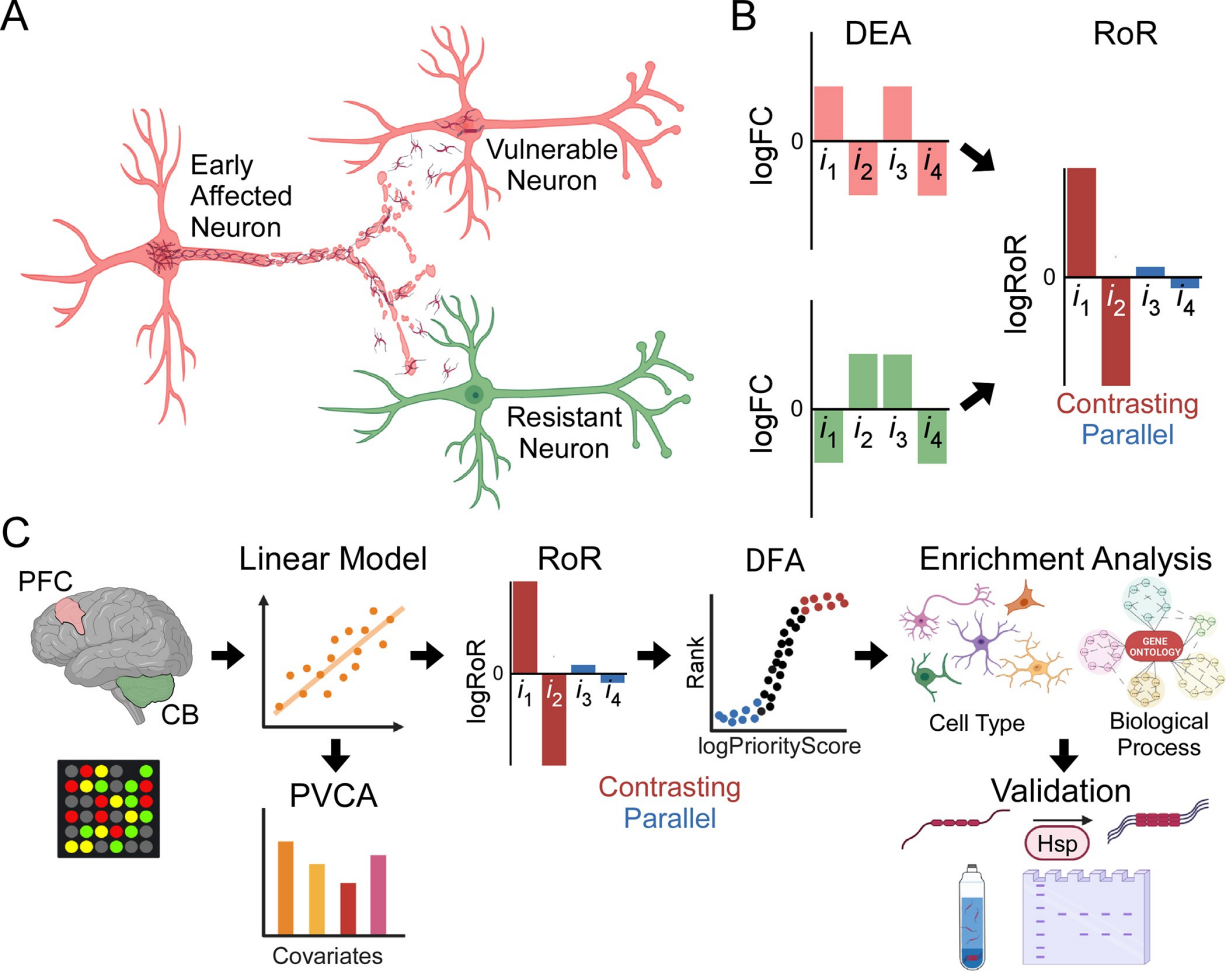
Approach for identifying gene expression changes associated with resistance to tauopathy. (**A**) Early affected neuron undergoing tauopathic neurodegeneration projects tau seeds to downstream neurons, some of which are vulnerable to prion-like templating and degeneration whereas others are resistant. (**B**) The ratio of ratios (RoR) approach extends differential expression analysis (DEA) by disaggregating region-specific log fold changes (logFC; disease versus normal control) into parallel and contrasting components, reflecting common and differing responses to tauopathy, respectively (illustrated for hypothetical gene transcripts *i*_1-4_). (**C**) Analysis pipeline from microarray measurement of gene expression to biological validation. See text and **S1 Code** for details. Created with BioRender.com.

Nonetheless, certain neuron populations in affected connectivity networks appear less vulnerable to tau lesion formation than others (**Fig 1A**). Primary neocortical regions, for example, develop tau pathology only late in disease [10]. This seemingly differential susceptibility to tauopathy results from their distal position in a connectivity chain rather than the vulnerability of their constituent cell populations to tauopathy. Conversely, the cerebellum (CB) neither develops tau lesions [11,12] nor experiences neuron loss [13] in LOAD despite being exposed to tauopathic insults early (owing to receiving direct input from the locus coeruleus [14]) and continuously (through connections to the default mode network [15]) throughout the course of disease. By the time AD cases enter late Braak stages, tau seeds in CB accumulate to experimentally detectable levels [16]. CB also atrophies [13] and develops amyloid plaques [11,12,17], but these pathological changes likely reflect degeneration of axons projecting to CB from AD-affected regions rather than local neuron loss (i.e., they reflect forms of Wallerian degeneration, [18]). CB is, therefore, a brain region that resists trans-synaptic spread of tau pathology despite being exposed to misfolded tau seeds throughout the course of LOAD. However, the granular layer of CB does develop tau lesions in early-onset AD caused by certain autosomal dominant mutations [19], indicating its neurons can be seeded by tau aggregates, but resist doing so owing to protective mechanisms that are breached only in the most aggressive forms of familial AD.

Resistance to tau lesion formation in CB could arise from intrinsic or adaptive mechanisms [20]. The former may reflect the vastly different transcriptional programming within constituent cells of CB relative to brain regions affected in AD [21], whereas the latter may stem from immune, stress and/or other responses to AD pathogenesis [22]. Intrinsic risk modulators that protect against or raise LOAD risk have been identified through genome-wide association studies (GWAS) [23–26], whereas adaptive mechanisms have been interrogated through proteomic and transcriptomic approaches [27–36]. Co-expression network analysis has identified rank-ordered “modules” (*i*.*e*., sets of genes that act in concert) that correlate with LOAD, with activation of immune/microglial-related genes being especially robust examples [36]. Although extension of the approach across multiple regions of interest has revealed region-to-region commonalities in the LOAD phenotype [36–42], it has not resolved local resistance of constituent cell populations from regional vulnerability arising from their positions within a connectivity chain.

Here we develop and test a ratio of ratios approach for characterizing adaptive changes to tauopathy and apply it to pre-frontal cortex (PFC) and CB in late-stage LOAD (**Fig 1B and 1C**). We show how this approach can be used to categorize expression changes associated with resistance to tauopathy in connected brain regions.

### Ratio of ratios model for analysis of gene expression

Standard omics analysis of LOAD leverages case-control samples to quantify log fold changes in expression (log *FC*) with disease (**Fig 1B**). The one-factor design of these studies sweeps up all differentially expressed genes, including those associated with cell death and related downstream events. Eq (1) applies this approach to the vulnerable PFC region for each gene product *i*, where the angular brackets represent the geometric average across all cases. Eq (2) does likewise for resistant region CB.


$$\log\;FC=\log_{2}\left(\frac{\langle {PFC}_{i}^{LOAD}\rangle }{\langle {PFC}_{i}^{Control}\rangle }\right)$$ (1)

$$\log\;FC=\log_{2}\left(\frac{\langle {CB}_{i}^{LOAD}\rangle }{\langle {CB}_{i}^{Control}\rangle }\right)$$ (2)

Reported results from this approach have emphasized common adaptive responses to LOAD in both regions at the transcript level with respect to expression modules [36] and affected cell types [42] despite their vastly different vulnerabilities to tauopathy. To better capture responses associated with resistance, we propose to combine Eq 1 and 2 to create a “ratio of ratios” (RoR) test for interregional commonalities and differences in gene expression. RoR calculations have been used extensively in biological and physical sciences to deliver relative quantification of gene expression [43,44] as well as to disaggregate data and dampen noise [45]. Application of this strategy to the problem of LOAD generates Eq (3):

$$log\;RoR={log}_{2}\left(\frac{\langle {PFC}_{i}^{LOAD}/{CB}_{i}^{LOAD}\rangle }{\langle {PFC}_{i}^{Control}/{CB}_{i}^{Control}\rangle }\right)$$ (3)

Here the numerator and denominator of Eq (3) are taken as the two ratios because of the availability of datasets containing matched samples in perfect registry with respect to age, sex, post-mortem interval, and other covariates. As a result, the ratios are calculated for every gene product *i* for every case before averaging to yield log RoR that is interrogated statistically using a single *t*-test (**Fig 1C**). In practice, however, Eq (3) can be rearranged owing to the quotient rule of logarithms to a form compatible with unmatched samples:

$$\log\;RoR=log_{2}FC\;PFC–log_{2}FC\;CB$$ (4)

When applied to PFC and CB regions, positive log RoR values arise from LOAD-dependent increases in expression in degenerating region PFC relative to resistant region CB, or through decreases in expression in CB relative to PFC. Conversely, negative RoR values result from LOAD-dependent decreases in PFC relative to CB expression, or from increased CB relative to PFC expression. As a result, the absolute value of log RoR magnitude provides an alternative metric for rank ordering gene expression changes associated with vulnerability. For example, parallel changes in LOAD-dependent expression occurring in both PFC and CB dampen log RoR value to near zero, reflecting lack of specific association with either region (**Fig 1B**). Conversely, AD changes occurring in contrasting directions between PFC and CB will show an amplified magnitude by RoR relative to Eq 1 or Eq 2 (**Fig 1B**).

Transcripts ranked by RoR can be further categorized through use of a desirability function [46] that numerically divides rank orders determined with Eq (3) by Eq (1). The resulting Priority Score *P* creates a continuous ranking of genes, where log *P* > 0 corresponds to contrasting genes that are amplified by RoR relative to PFC-only, and log *P* < 0 corresponds to parallel genes that are dampened by RoR relative to PFC-only. Desirability function analysis (DFA) categorizes RoR data in two ways. First, LOAD-dependent expression changes associated with cell death or other processes occurring in PFC but not in resistant CB rank similarly when analyzed by either Eq (1) or (3) and therefore distribute around log *P* = 0. In contrast, genes associated with processes occurring selectively in CB distribute with log *P* >> 0. As a result, contrasting expression associated with resistant CB region can be separated from events in vulnerable PFC. These transcripts are of special interest because they include differential disease responses associated with adaptive resistance in CB. Second, LOAD-dependent changes that occur in parallel in both regions will distribute with log *P* << 0. These transcripts are of interest because they reflect disease processes common to both regions including Wallerian degeneration and amyloid plaque deposition.

## Results

### Data overview

**Fig 2:**
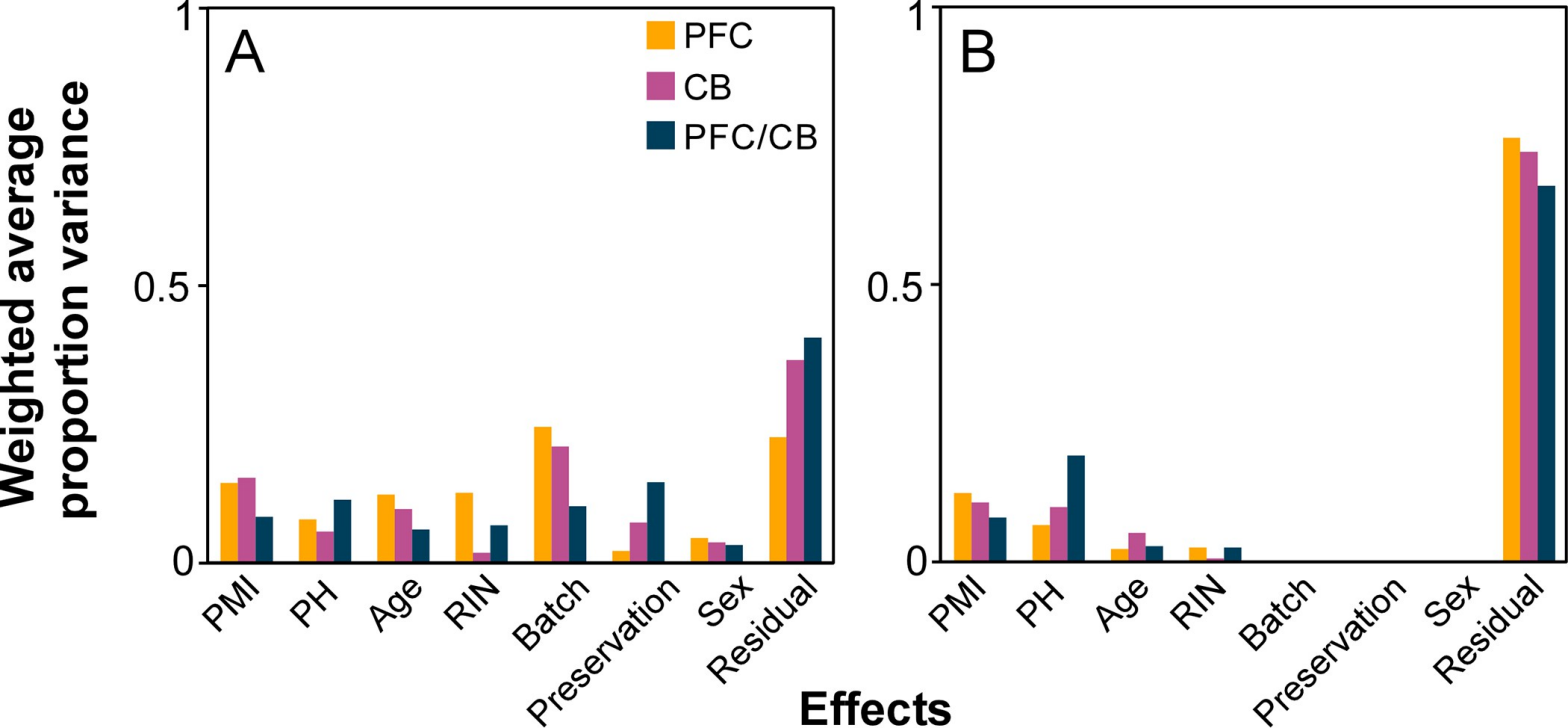
PVCA of array data. Each bar represents the proportion of total variance attributable to its associated factor before (**A**) and after (**B**) linear modeling. Because the ratio groups had pH and RNA-integrity numbers from each tissue, these PVCA calculations included nine covariates whereas PFC-only and CB-only analyses included seven covariates. Although the interregional PFC/CB ratio attenuated the variance owing to covariates, linear modeling was required to minimize their effects.

To identify gene products associated with LOAD resistance, microarray data deposited by the Harvard Brain Tissue Resource Center (Gene Expression Omnibus repository: GSE44772; [36]) was interrogated using the workflow summarized above (**Fig 1C**). This dataset leverages a 39,280 gene probeset of which 25,852 map to named genes. It was chosen for analysis because of its regions of interest, which includes matched samples from the resistant CB and vulnerable PFC for each individual (i.e., dorsolateral PFC, Brodmann area 9), its large sample size (129 LOAD and 101 control replicates), which provides substantial statistical power, and its preparation from tissue homogenates, which captures the broad range of cell types affected in LOAD [42]. It also provides covariates associated with patients (disease status, age, post-mortem interval, sex, and Braak stage; **Table A in S1 Data**) and samples (pH, RNA integrity number, preservation method, and batch; **Table B in S1 Data**). To estimate the contribution of covariates to overall variation in these data, and the effectiveness of Eq (3) and model linearization at removing it, principal variance component analysis (PVCA; [47,48]) was performed. For non-linearized data, batch effects, post-mortem interval, pH, age, RNA integrity, preservation method, and sex explained the majority of total variance so that only 23–37% was random (**Fig 2A**). The interregional PFC/CB fold change calculation (i.e., the numerator and denominator ratios of Eq (3)) modestly depressed non-random contributions, yielding a modest improvement (**Fig 2A**). However, this improvement paled in comparison to linear modeling, which completely removed batch effects and raised overall random variance to >65% of total **(Fig 2B)**. Overall, PVCA analysis indicated that batch effects and other covariates do not necessarily affect brain regions identically, and that minimizing their impact required linearization prior to calculation of Eq (1) or (3).

### Prioritization

**Fig 3:**
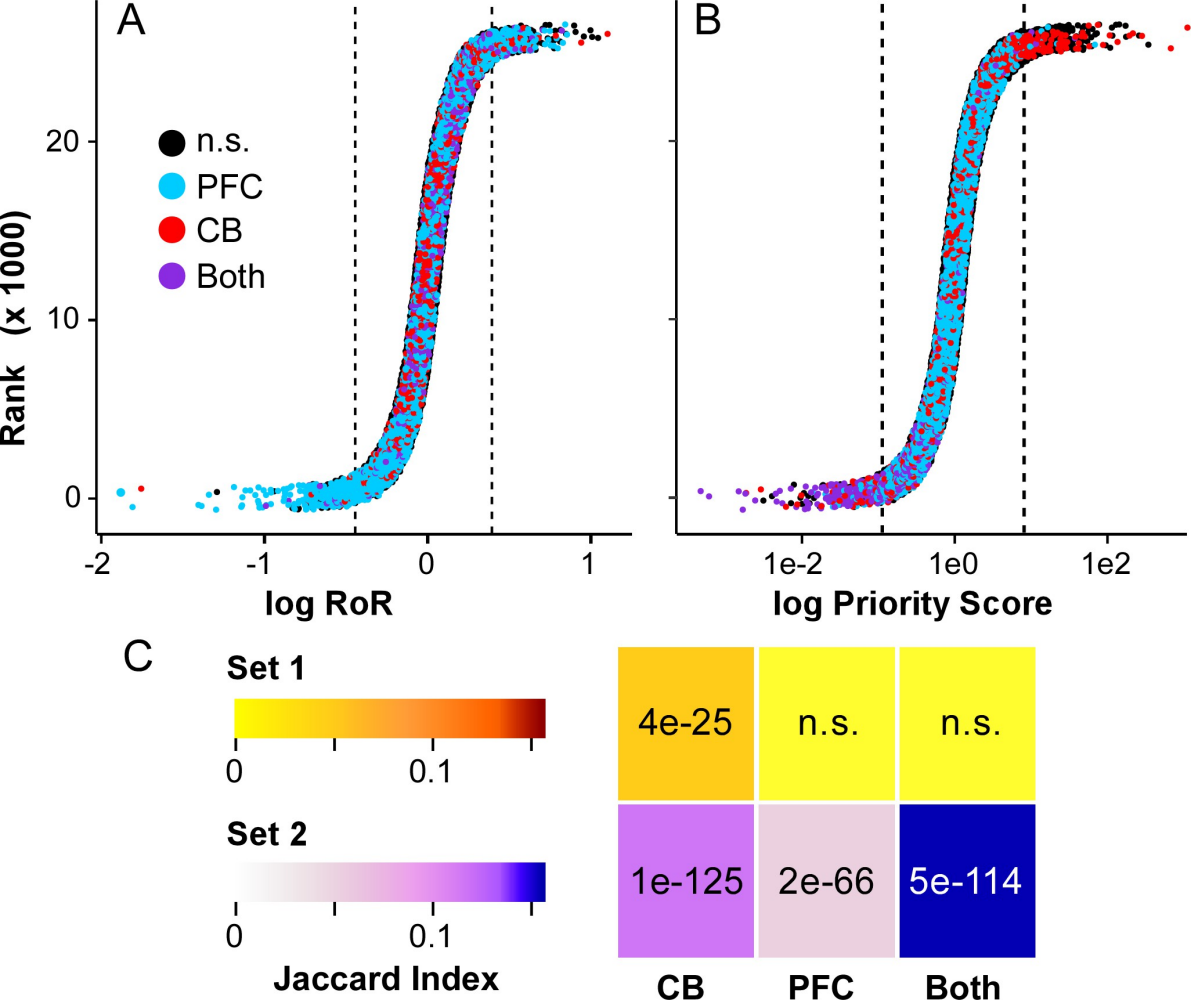
Rank distribution of named transcripts after RoR analysis. Jitter plots, where data points represent 25,852 named genes. Those corresponding to previously identified Braak correlated genes in PFC only (blue), CB only (red) or both PFC and CB (purple) are highlighted. All other genes are colored black. Dashed lines correspond to top and bottom 500 transcripts in each distribution. (**A**) Distribution of log RoR values, where Braak correlated genes show an even distribution across the sigmoid curve. (**B**) Distribution of log Priority Scores after subjecting RoR data to DFA. See text for details. (**C**) Enrichment analysis of Gene Sets 1 and 2 with Braak correlated transcripts, where color gradients indicate overlap by Jaccard index and numerals indicate Benjamini-Hochberg corrected *p*-values calculated by Fisher’s Exact Test. See text for details.

To disaggregate data according to relative change between regions of interest, the 25,852-member probe set corresponding to named genes was analyzed by Eq (1) and (3) to yield lists of gene expression changes with LOAD ranked by PFC region only and by RoR (**Table C in S1 Data**). These were then subjected to DFA to identify parallel and contrasting components. Results are illustrated by highlighting the distribution of 3,757 Braak Stage correlated transcripts previously identified in GSE44772 by Zhang et al. [36]. Plotting rank versus log RoR revealed that all named transcripts distributed across three log2 units (**Fig 3A**). Transcripts that correlated with Braak stage in PFC scattered evenly across the entire distribution, whereas those in CB or in both PFC and CB dispersed more centrally (**Fig 3A**). These results are consistent with larger LOAD effects in PFC than in CB in this dataset [36]. Subsequent DFA yielded a monotonic sigmoid distribution of priority scores centered on the *y*-axis and spanning ~7 orders of magnitude (**Fig 3B**). In this plot, however, Braak correlated transcripts distributed such that those observed only in CB displayed large log *P* values, whereas those common to PFC and CB displayed very small log *P* values. In contrast, PFC-specific Braak correlated genes distributed mostly within two orders of magnitude of log *P* = 0 (**Fig 3B**).

The upper and lower limbs of the prioritization curve corresponded to ~500 probes each, which provided a symmetric threshold to define the genes of interest for detailed analysis (**Fig 3B**). These were further filtered by RoR *t*-test, where *p* < 0.05 was required to confirm contrasting expression, whereas *p* ≥ 0.05 was required to define parallel expression. Out of the top 500 probes, the filter for contrast was met by 432 transcripts distributed among 402 unique genes. This gene set was termed “Set 1” (**Table D in S1 Data)**. In contrast, out of the bottom 500 probes (i.e., those with lowest Priority Scores), the filter for parallel expression was met by 442 transcripts distributed among 404 unique genes. This gene set was termed “Set 2” (**Table E in S1 Data**). Gene Sets 1 and 2 were then subjected to enrichment and transcription factor network analyses described below.

### Braak stage enrichment analysis

As explained above, contrasting Set 1 and parallel Set 2 transcripts were both expected to reflect disease processes associated with LOAD. To test whether these processes included pathological tau burden, their overlap with Braak stage correlated genes was investigated. Results showed that Set 1 enriched with transcripts correlating with Braak stage in CB but not in PFC (**Fig 3C**). Although Set 2 displayed an even stronger enrichment with transcripts correlating with Braak stage in CB, it differed from Set 1 by also enriching with Braak correlated PFC transcripts (**Fig 3C**), consistent with it being defined on the basis of parallel expression. These data indicate that the RoR approach could identify genes of interest associated with a LOAD pathology-related phenotype (i.e., correlation with Braak stage) while disaggregating them into two distinct components defined by their contrasting or parallel nature.

### Cell type-specific enrichment analysis (CSEA)

**Fig 4:**
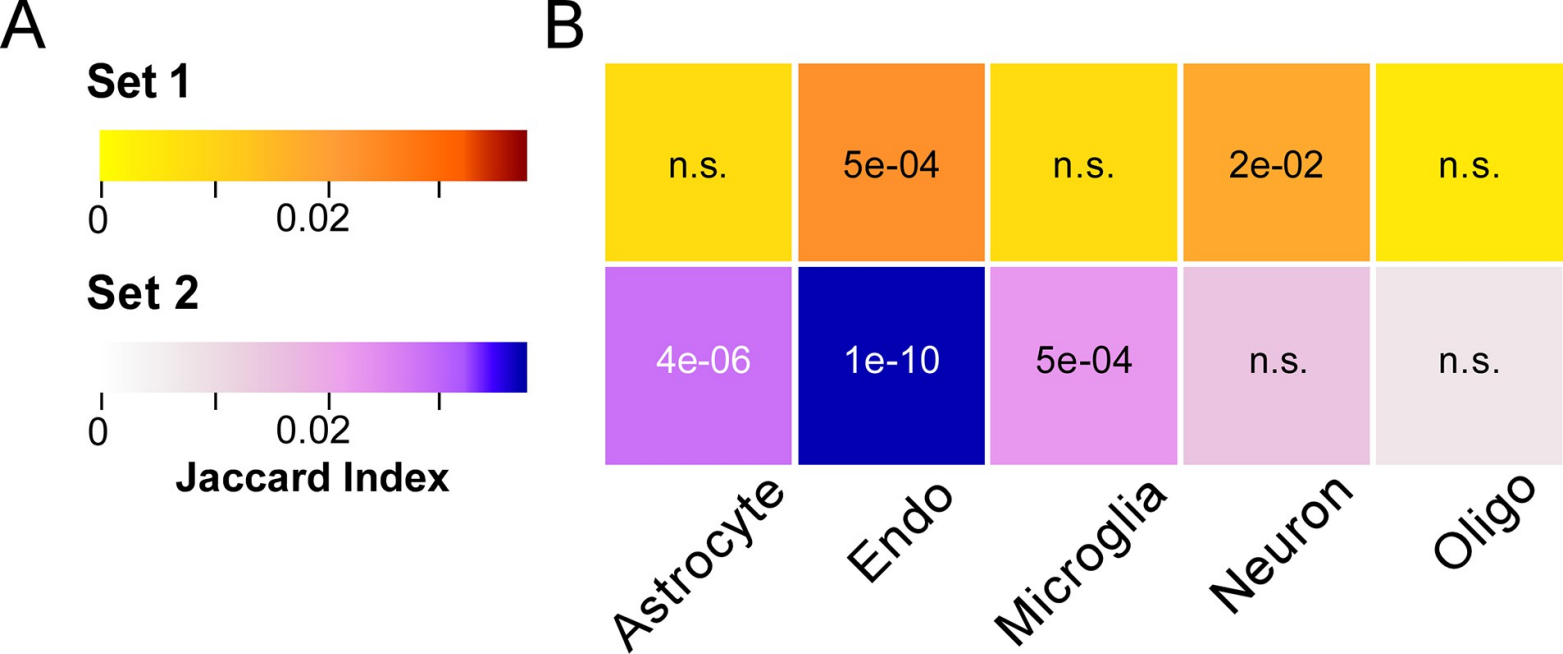
Enrichment analysis of Gene Sets 1 and 2. Overlap comparison of genes in Set 1 and 2 with previously reported cell-type specific markers from human brain [49] (Endo, endothelial cells; Oligo, oligodendrocytes), where color gradients indicate overlap by Jaccard index and numerals indicate Benjamini-Hochberg corrected *p*-values calculated by Fisher’s Exact Test. Both Sets 1 and 2 enriched for endothelial cell specific genes, while Set 1 enriched uniquely for neuron specific markers, and Set 2 uniquely for astrocyte and microglia specific markers.

To identify which cell types contributed to contrasting and parallel expression patterns, the overlap between Sets 1 and 2 and human brain cell type specific marker genes defined by immunopanning- and flow cytometry-based methods was explored [49]. Set 1 demonstrated enrichment of neuronal and endothelial cell-specific genes (**Fig 4**). Set 2 also showed enrichment for endothelial cell-specific genes, but otherwise enriched for astrocyte- and microglia-specific transcripts (**Fig 4**). The enrichment of neuronal genes among Set 1 genes supports the premise that Set 1 genes represent genes affecting NFT formation, a neuronal phenotype. Similarly, the enrichment of astrocytic and microglial genes among Set 2 genes suggests this gene Set associates with microglial activation and astrogliosis in AD brain. The shared enrichment of endothelial genes between Sets 1 and 2 suggests these cells may contribute to multiple aspects of AD pathogenesis.

**Fig 5:**
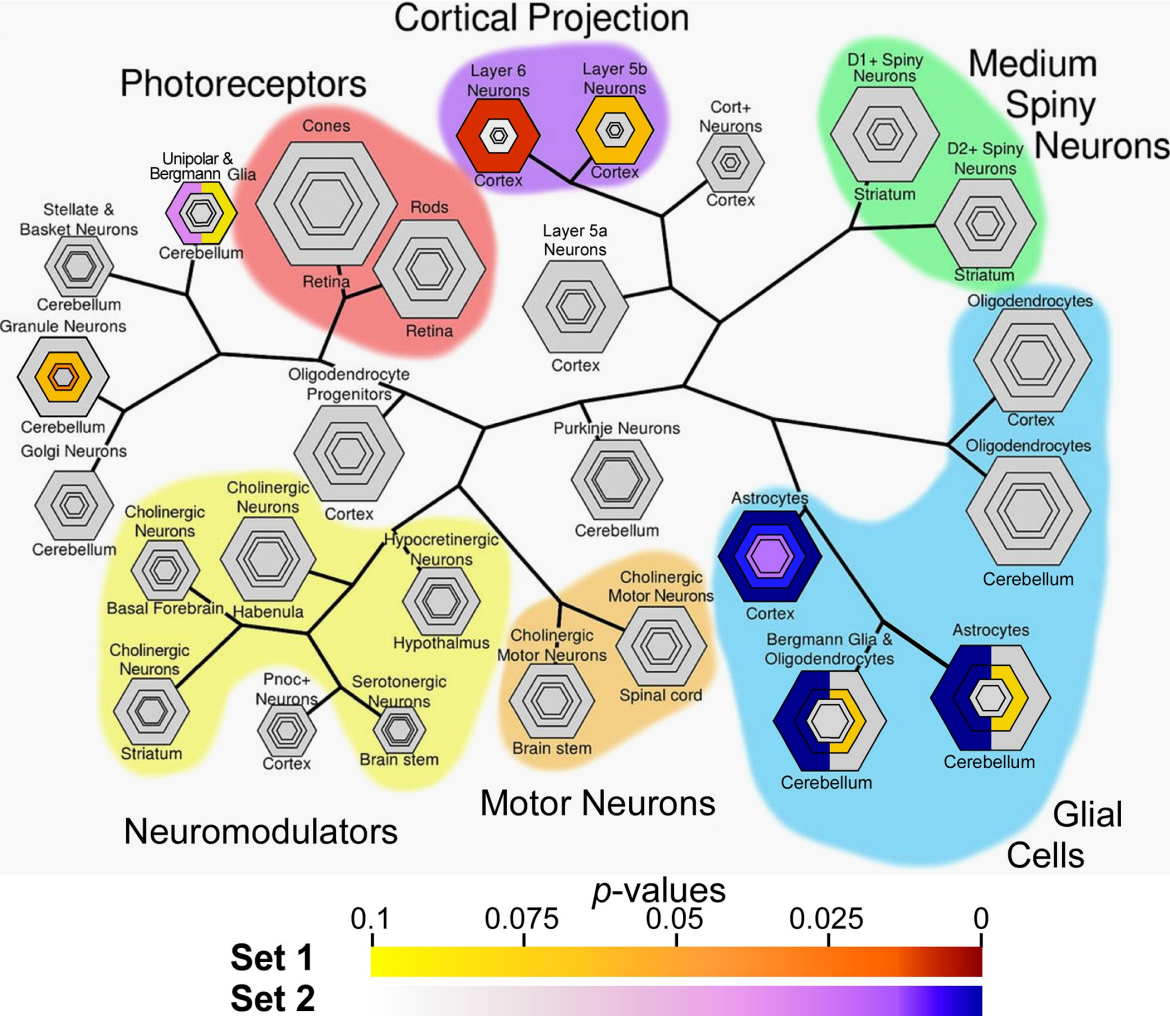
Cell specific enrichment analysis (CSEA) of Gene Sets 1 and 2. Bulls-eye plots, where hexagon size scales with specificity for listed cell types. Each concentric ring conforms to specificity index probability statistic (pSI) at thresholds of 0.05, 0.01, 0.001 and 0.0001 (ordered from outermost to innermost ring), where lower thresholds represent higher cell specificity. Statistical overlap between gene sets with cell-type specific markers are depicted according to Benjamini-Hochberg adjusted *p*-values. Gene Set 1 uniquely enriched for genes expressed by layer 5b and layer 6 neurons of the cortex and granule neurons of the CB, and more weakly with unipolar cells/Bergmann glia of CB. In contrast, Set 2 genes enriched with astrocytes very strongly across all levels of specificity, and more weakly with unipolar cells/Bergmann glia of CB. Neither gene set associated significantly with oligodendrocytes.

To refine this analysis, cell-type enrichment was further interrogated using “CSEA tool”, an algorithm that quantifies enrichment in terms of relative specificity for individual neuronal and glial cell subtypes [50,51]. This tool was chosen because it was trained on actively translated transcripts and includes neocortical and CB regions of interest. As a result, it resolves neurons and glia into subpopulations by function as well as location and morphology [52]. Results confirmed that Set 1 transcripts enriched in neurons, with highest specificity for granule neurons of CB (**Fig 5**). Enrichment also was observed in excitatory unipolar brush interneurons residing in the granular layer, but not in Purkinje cells or in inhibitory interneurons of the stellate, golgi, or basket cell classes (**Fig 5**). In neocortex, Set 1 transcripts enriched with greatest specificity in layer 6, with low specificity in layer 5b, and not at all in layer 5a of neocortical neurons (**Fig 5**). The enrichment order layer 6 > 5b > 5a correlated inversely with the reported frequency of Aβ [53] and tau [54] lesions in these lamina. Nonetheless, Set 1 transcript enrichment was not limited to neurons. In CB, this gene set also associated with moderate specificity with astrocytes (including Bergmann Glia, which are unipolar astrocytes that modulate synaptic activities in the Purkinje layer of CB, reviewed in [55]), but not with mature or precursor oligodendrocytes in any brain region (**Fig 5**). These data indicate that Set 1 transcripts enrich primarily in excitatory neurons and secondarily with astrocytes of CB region. Contrary to this pattern, Set 2 transcripts appeared almost exclusively glial, with strong enrichment in astrocytes within CB and PFC at multiple levels of specificity (**Fig 5**). As with Set 1 transcripts, no association with mature or precursor oligodendrocytes was detected (**Fig 5**). Taken together, CSEA results indicate that Set 1 transcripts potentially associated with resistance to tauopathy enrich primarily with excitatory neurons of the granular layer of CB, whereas Set 2 transcripts associated primarily with microglia and astrocytes undergoing similar adaptive changes within resistant CB and vulnerable PFC regions.

### Differential expression analysis

**Fig 6:**
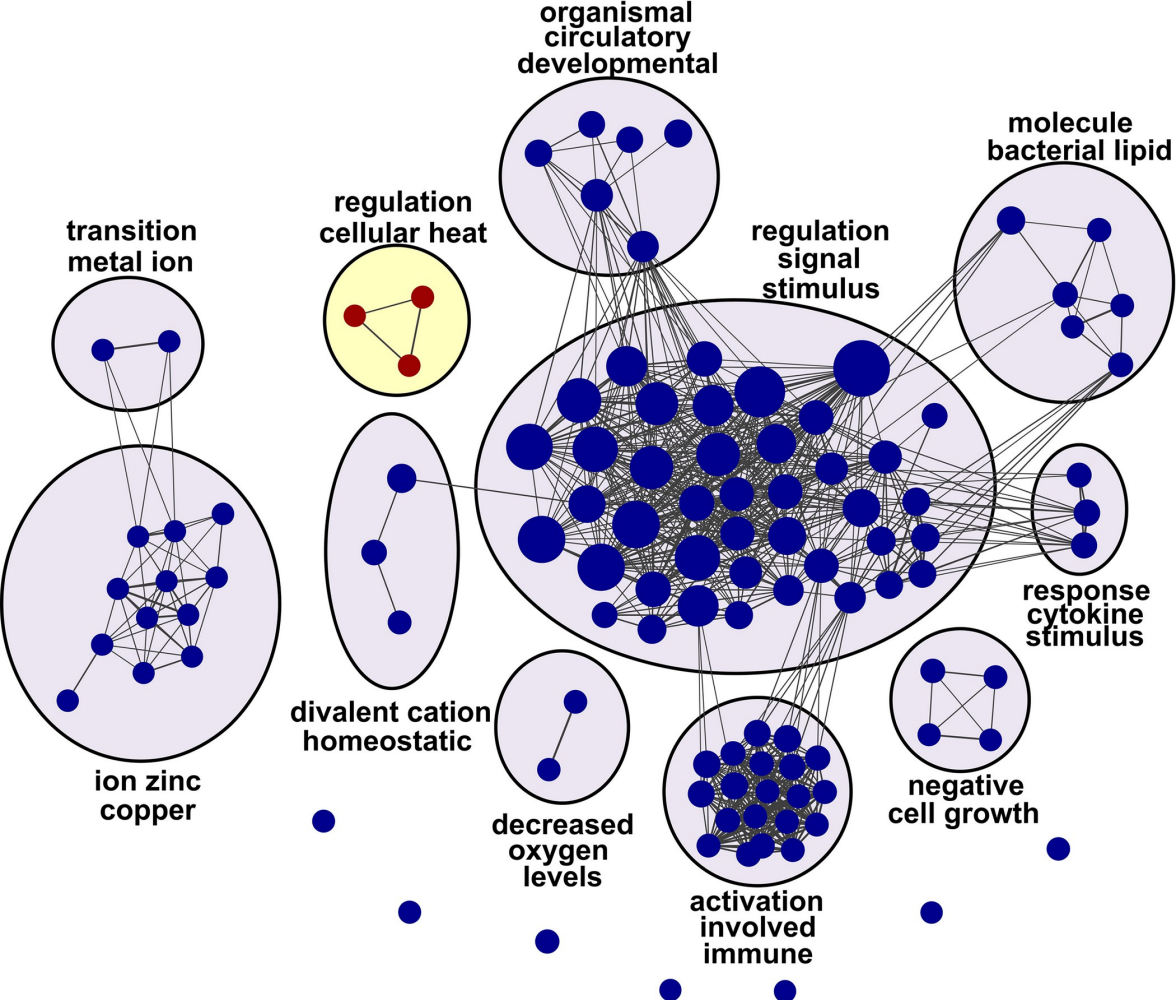
Gene set enrichment analysis (GSEA) of Gene Sets 1 and 2. Enrichment maps of biological process gene sets defined by the Gene Ontology Biological Processes database, where each node represents a GO category and node diameter is proportional to the number of genes in each gene set (red nodes, contrasting Set 1; blue nodes, parallel Set 2). Edges indicate overlapping genes between nodes, where edge width is proportional to the Jaccard index of overlap between the two gene sets. Enrichment maps highlight heat shock response and chaperone genes among contrasting Gene Set 1, and homeostasis and immune genes among parallel Set 2. List of genes in enriched GO categories available in supplemental data (Tables F and G in S1 Data).

**Fig 7:**
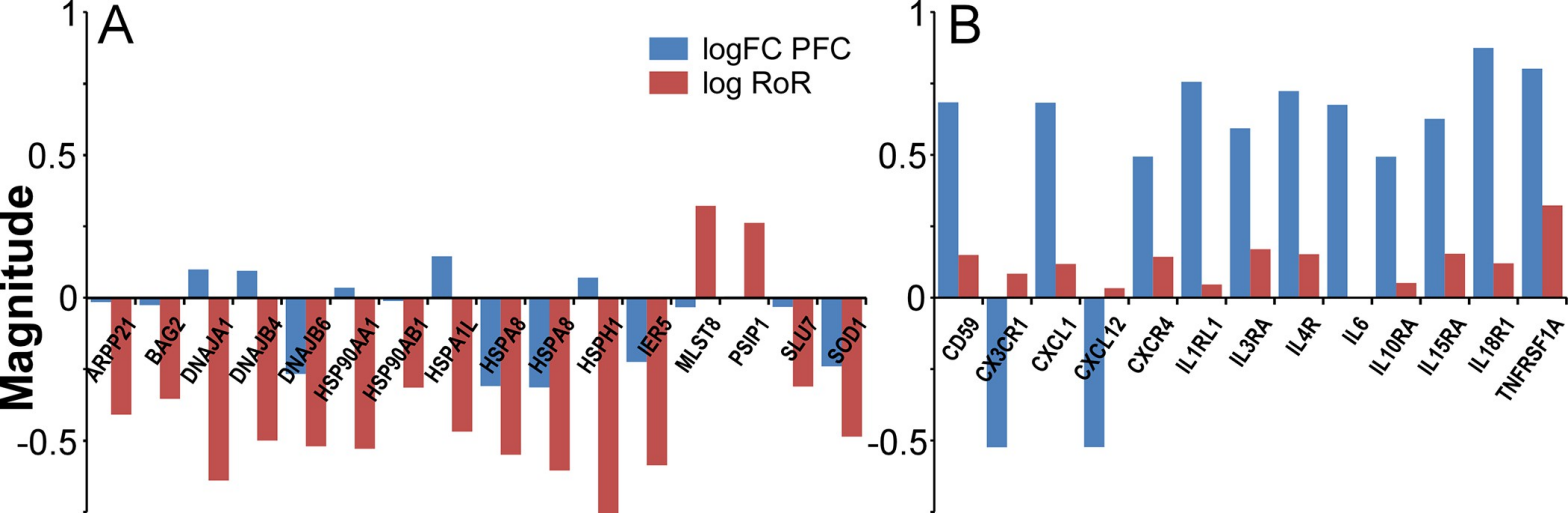
Differential expression of individual members of Gene Sets 1 and 2. The magnitude of log FC and log RoR calculated by Eq (1) (PFC region, blue) and (3) (log RoR, red) were plotted for selected GO biological process enriched genes identified in **Fig 6**. (**A**) Contrasting Set 1 genes annotated to “heat cellular temperature” GO categories. Log RoR reveals polarity of changes with respect to tauopathy vulnerability. (**B**) Set 2 genes belonging to interleukin and cytokine signaling pathways. Log RoR values trend toward zero owing to both CB and PFC changing expression in parallel.

To characterize functional relationships among transcripts identified by RoR prioritization, each gene set was tested for enrichment of specific gene ontology (GO) and functional pathway terms (gene set enrichment analysis; GSEA [56–58]). Three biological processes were enriched in contrasting Set 1 transcripts, all of which were associated with proteostasis and heat shock response components (**Fig 6**). When viewed at the single gene level, these transcripts showed little or no change with LOAD in PFC region, but substantial change with LOAD according to RoR analysis (**Fig 7A**). For molecular chaperones *BAG2*, *DNAJA1*, *DNAJB4*, *DNAJB6*, *HSP90AA1*, *HSP90AB1*, *HSPA1L*, *HSPA8*, *and HSPH1*, transcriptional regulator *IER5*, and antioxidant *SOD1*, fold change correlated inversely with LOAD vulnerability (**Fig 7A**). The negative polarity with vulnerability arose not because these transcripts were depressed in vulnerable PFC region, but because all became elevated with disease in resistant CB (**Fig 7A**).

In contrast to these findings, Set 2 transcripts enriched with several dense webs of biological processes related to cellular stress and immune categories, including leukocyte activation, response to low oxygen or bacterial lipids, metal ion homeostasis, and negative growth regulation (**Fig 6**). These processes relate most closely to the functions of glial and microglial cells, in accordance with CSEA characterization of this gene set. Although transcripts associated with stress processes showed marked changes with LOAD in PFC, the changes were greatly attenuated after RoR analysis regardless of their polarity. For example, members of Set 2 transcripts associated with interleukin and cytokine signaling pathways implicated in LOAD (reviewed in [59]), including pro-inflammatory IL6 and TNFα receptor *TNFRSF1A*, followed this pattern (**Fig 7B**). Moreover, the pro-inflammatory “master regulator” *IL1β* also was strongly upregulated in both regions, though it did not rank in the top 500 transcripts by priority score (**Table C in S1 Data**). These data reveal that neuroinflammatory processes were up regulated in both vulnerable and resistant brain regions, suggesting they more closely associated with the reactive gliosis and Wallerian degeneration occurring in both regions rather than with active spread of tauopathy. The pattern also extended to certain protective immune regulators in Set 2, including *CX3CR1*, the receptor for fractalkine (*CX3CL1*), which was strongly depressed in CB but also in PFC, and *CD59*, an antagonist of membrane attack complex, which was strongly up regulated in both regions (**Fig 7B**). These data dissociate key elements of neuroinflammation and reactive gliosis from active spread of tau pathology.

**Fig 8:**
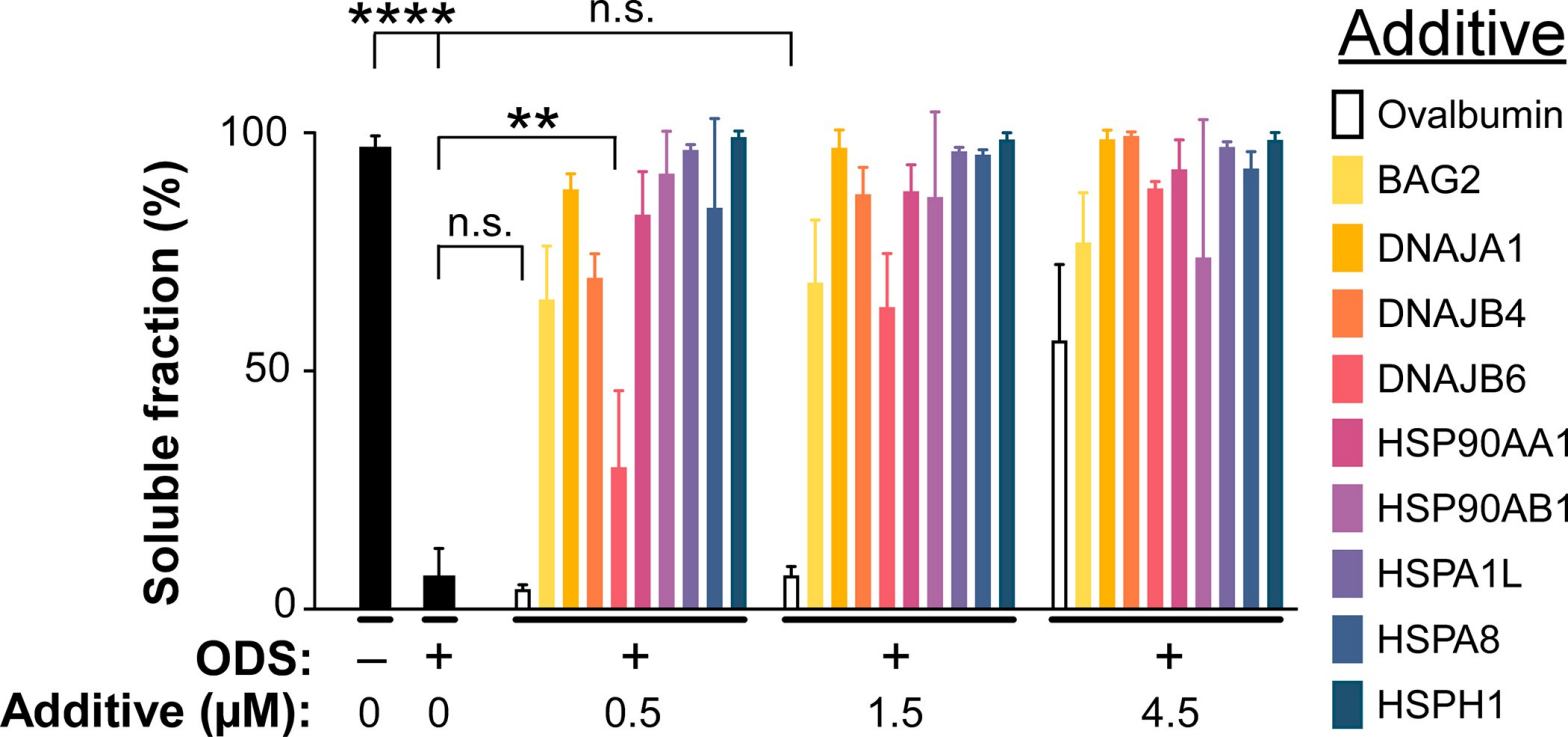
Molecular chaperones associated with tauopathy resistance antagonize 2N4R tau aggregation at sub-stoichiometric concentrations. Full length 2N4R tau (1.5 μM) was incubated (16 h at 37°C) with or without ODS inducer (50 μM) in assembly buffer and various concentrations of each chaperone or ovalbumin control protein (0, 0.5, 1.5, 4.5 μM). Aggregation products were centrifuged and the amount of tau protein present in supernatant and pellet fractions determined by SDS-PAGE. Each bar represents the proportion of tau in the supernatant fraction (triplicate determination ± S.D.). n.s. = not significant, ** *p* < 0.01, **** *p* < 0.0001 for comparison with soluble tau levels in ODS control samples. All comparisons to ODS control were significant at to the level of p < 0.0001, unless marked otherwise. Although tau remained completely soluble under these conditions in the absence of ODS inducer, the presence of ODS shifted tau out of the soluble fraction into insoluble forms. The additional presence of chaperone proteins depressed aggregation, leading to concentration dependent increases in soluble tau relative to the ODS only control.

GSEA data suggest that RoR analysis has the potential to unmask correlations while establishing their polarity with respect to vulnerability to tauopathy. To test the polarity of gene products prioritized by the approach, all nine molecular chaperones identified in Set 1 were expressed in bacteria, purified and then tested for ability to chaperone tau misfolding in an in vitro model of tau fibrillation. These assays capture inhibition of tau aggregation resulting from direct chaperone binding to tau monomers and aggregates to depress fibril nucleation and elongation [60–73]. ATP was omitted as co-substrate because chaperone disaggregase activity, which is ATP dependent [67], has not been reported against tau aggregates [70]. Assays leveraged the full-length human 2N4R tau isoform and octadecyl sulfate (ODS) as aggregation inducer because these conditions foster completion of nucleation, growth and plateau aggregation phases within 16 h incubation under near physiological conditions of ionic strength, pH and low micromolar tau concentrations [74]. In addition, 2N4R is the longest isoform expressed in the adult human central nervous system [75], ensuring that all tau sequence motifs in human brain were present in the assays. In the absence of chaperones, tau aggregation was completely dependent on the presence of ODS inducer (**Fig 8**). The presence of molecular chaperones at 1:3 and 1:1 stoichiometries inhibited ODS-induced 2N4R tau aggregation in a concentration dependent manner (**Fig 8**). However, ovalbumin, a control protein devoid of chaperone activity, had no statistically significant effect on aggregation. Although further increases in chaperone concentrations to 4.5 μM (i.e., 3:1 stoichiometry with respect to tau protein) completely inhibited aggregation, ovalbumin too was inhibitory under these conditions, indicating that non-specific effects dominated at high protein concentrations. These data confirm the importance of working at low chaperone and tau concentrations when assessing aggregation propensity, and show that all molecular chaperones identified as a result of GSEA antagonize tau aggregation in vitro.

These data provide evidence that disaggregation of data through RoR analysis can yield insight into the polarity of adaptive changes with respect to LOAD resistance. In the case of molecular chaperones, they implicate induction of specific members of different chaperone structural classes as one mechanism through which resistance to tauopathy is generated in CB.

### Interaction network analysis

**Fig 9:**
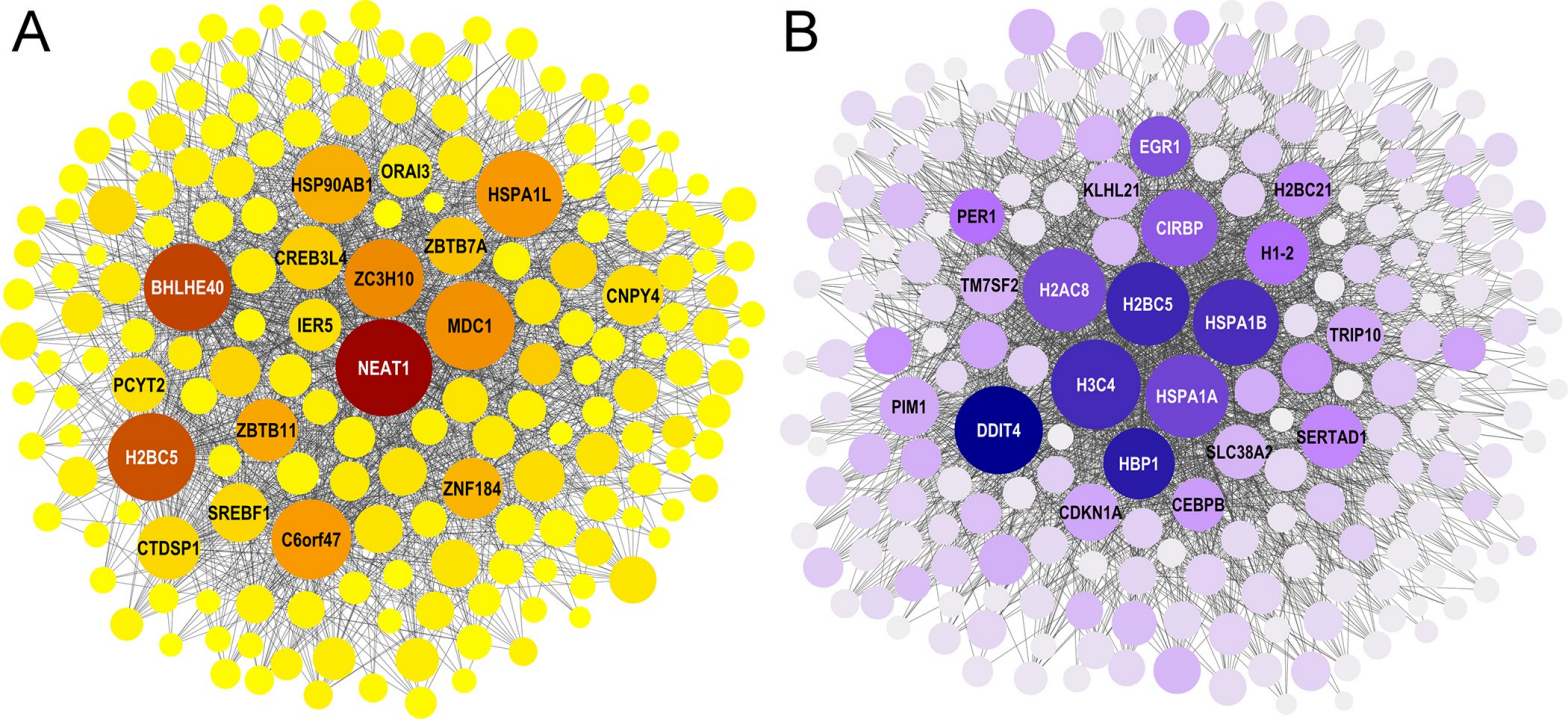
Interaction networks generated from Gene Sets 1 and 2. Node size represents degree centrality (i.e., increasing size represents greater numbers of edges intersecting the node) whereas color gradient represents betweenness centrality (i.e., increasing color depth represents greater numbers of shortest paths passing through the node). Networks filtered to top 20 nodes by degree and nearest neighbors for simplicity (but all nodes are listed in Tables H and I in S1 Data). (**A**) Contrasting Gene Set 1 seeded network. *NEAT1*, a long noncoding RNA, was the most central node by both degree and betweenness criteria. (**B**) Parallel Gene Set 2 seeded network. Target genes *DDIT4*, *HSPA1A*, *HSPA1B*, and several histone genes were the most central nodes.

The identification of transcription factor *IER5* in Set 1 by GSEA indicates that resistance may be coordinated at the transcript level. To identify additional candidate hub regulators, Gene Sets 1 and 2 were interrogated using NetworkAnalyst 3.0 [76]. This tool maps enriched genes onto specific interactions between transcription factors and their targets established by means of chromatin immunoprecipitation sequencing data, while quantifying the centrality of each network node according to degree (i.e., a direct count of nearest neighbors) and betweenness (i.e., a count of shortest paths through the node) criteria [76]. When applied to contrasting Set 1 transcripts, the 319 gene set members recognized by the ENCODE database seeded a 651-node network containing 8,597 edges. The top 20 nodes ranked by degree centrality contained 18 members of Set 1, including *IER5* and chaperones *HSPA1L* and *HSP90AB1* (**Fig 9A**). Other transcription factors within the top 20 nodes included *BHLHE40*, *CREB3L4*, *SREBF1*, *ZBTB7A*, *ZBTB11*, *ZNF184* and *ZC3H10*. However, the most central node by both degree and betweenness measures was the long noncoding RNA (lncRNA) *NEAT1*, a nonprotein transcriptional regulator. Although *NEAT1* was elevated in vulnerable PFC region, it was strongly depressed in CB, making its overall positive correlation with vulnerability among the highest calculated based on RoR fold change (**Table D in S1 Data**). These data implicate depression in *NEAT1* lncRNA expression with resistance in the CB region.

In contrast to these findings, parallel Set 2 transcripts yielded 379 recognized genes that seeded a distinct 678-node network containing 8,594 edges, with the top 20 nodes by degree centrality all being members of Set 2 (**Fig 9B**). Among these were the transcription factors *CEBPB*, *EGR1*, *HBP1*, *PER1* and *SERTAD1*. However, the most central node by degree was *H3C4*, a member of the histone family associated with epigenetic regulation [77], whereas the most central by betweenness (and second most central by degree) was *DDIT4*. Both *H3C4* and *DDIT4* were highly overexpressed in LOAD, but appeared in Set 2 because this change occurred in both PFC and CB regions (**Table E in S1 Data**). *DDIT4* has been reported to up-regulate in response to Aβ [78,79], and so is positioned to mediate transcriptional regulation associated with reactive gliosis occurring in both regions rather than the active spread of tau pathology. Overall, interaction analysis indicated that the responses to LOAD identified in gene sets 1 and 2 were mediated by vastly different programs.

### Overlap with genetic loci

**Fig 10:**
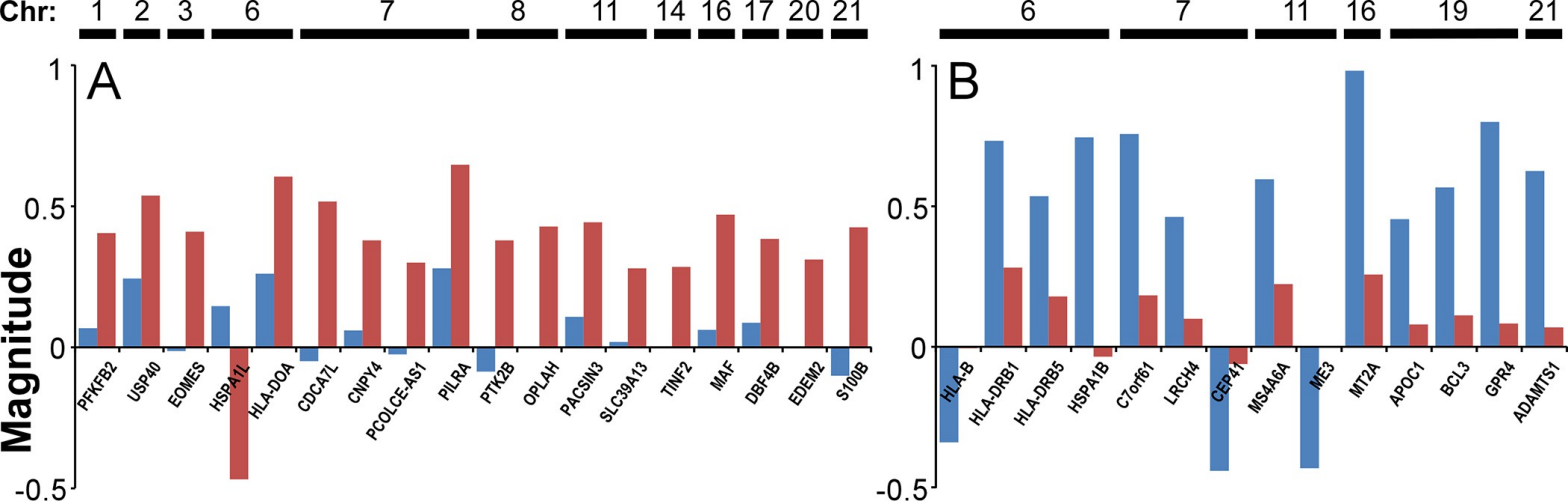
Differential expression of LOAD risk factor genes in Gene Sets 1 and 2. The magnitude of log FC and log RoR calculated by Eq (1) (PFC region, blue) and (3) (RoR, red) were plotted for risk factor loci-associated genes identified in (**A**) Set 1 (Table H in S1 Data) and (**B**) Set 2 (Table I in S1 Data), where chr signifies chromosomal assignment. RoR reveals polarity of candidate risk gene expression changes with respect to tauopathy vulnerability.

At least 38 genetic loci have been implicated in LOAD [25,26,80]. Because most genetic variation within them is intronic and intergenic [25], each locus could potentially affect the expression of multiple genes. Positional mapping, linkage disequilibrium boundary, expression quantitative trait loci (eQTL), and chromatin interaction mapping studies have implicated as many as 1239 candidate effectors at these loci [25, 26, 80]. The polarity of expression with respect to LOAD vulnerability has been investigated for only a minority of them. To identify candidate causal mediators of vulnerability to tauopathy, Gene Sets 1 and 2 were investigated for overlap with candidate LOAD effectors. Out of the 854 candidate mediators quantified in dataset GSE44772, 18 associated with primarily neuronal Set 1 whereas 14 associated with primarily glial/microglial Set 2 (**Fig 9** and **Tables J and K in S1 Data**). Loci on chromosomes 6, 7 and 19 appeared as “hotspots”, with multiple candidates at each locus appearing in the gene sets (**Tables J and K in S1 Data**). When viewed at the single gene level, the polarity of candidate effectors in Set 1 was mostly positive (i.e., increased expression correlated with vulnerability to tauopathy), not because these transcripts were elevated in vulnerable PFC region, but because they were depressed in resistant CB (**Fig 10A**). Two of these genes, *PILRA* and *PTK2B*, have been reported to recapitulate this polarity on eQTL analysis [81]. These data support use of RoR analysis to deduce polarity of candidate causal genes in the context of resistance to tauopathy.

In contrast to these findings, Set 2 causal mediators shared reduction in prioritization after RoR calculation regardless of polarity (**Fig 10B**). One of these genes, *MS4A6A*, has been reported to correlate positively with LOAD risk [81]. These data position Set 2 causal mediators in association with Wallerian neurodegeneration and reactive gliosis common to both PFC and CB regions. Overall, these data show that disaggregation of gene expression data through RoR analysis has the potential to provide novel insight into polarity and functionality of causal mediators identified on the basis of genetics.

## Discussion

Previous investigations of changes in gene expression between LOAD and cognitively normal control cases have focused on commonalities among multiple brain regions regardless of their vulnerability to trans-synaptic spread of tau pathology [36–42]. Here we found that disaggregation of these data through RoR analysis provided complementary information on the changes in gene expression that accompany LOAD. The approach is simple, as it is governed by a single *t*-test, and transparent, as the results of prioritization can be readily visualized. When applied to differentially vulnerable brain regions, the analysis quantifies the polarity of expression change with respect to regional vulnerability, rather than mere presence of disease. Although demonstrated using gene array data collected from brain homogenates, the approach also is compatible with proteomics data and may be especially useful when interrogating disease-associated changes at the single cell level. Moreover, it may be applicable to other neurodegenerative disorders mediated by prion-like trans-synaptic spread of protein aggregates through connectivity networks.

Nonetheless, the approach also faces limitations. First, the workflow adopted here leveraged matched samples where interregional covariates were in perfect registry, a condition which may not be available in other datasets. However, PVCA analysis showed that matching alone had little effect on covariate contributions to variance, and that the major processing step needed to maximize random variance was linear modeling. This result suggests that a simplified form of RoR analysis embodied in Eq (4) will be adequate for datasets that lack interregional matching. Second, positive RoR tests can arise from changes in either of the regions under investigation, and so may reflect gross tissue changes associated with cell death rather than modulation of specific molecular pathways. However, the DFA implemented here minimized contributions from the degenerating region so that changes occurring in the non-degenerating region could be characterized selectively.

When administered to resistant CB and vulnerable PFC regions, RoR analysis revealed substantial differences in regional responses to LOAD. With respect to contrasting Set 1, several lines of evidence suggest it overlaps with a putative tauopathy resistance signature in CB. First, Set 1 is enriched in Braak stage correlated transcripts from resistant CB but not vulnerable PFC, indicating that it selectively appears in response to pathological tau burden. Second, it derived primarily from excitatory neuronal cell types and enriched with molecular chaperone genes having negative polarity with respect to vulnerability to tauopathy. Although proteostasis factors predicted by literature references have been implicated in intrinsic resistance to tauopathy [82], here nine specific members of the chaperone family were found elevated as part of an adaptive response to LOAD tauopathy. Using in vitro tau aggregation assays, we validated all nine as being capable of depressing tau fibrillation at substoichiometric ratios with respect to physiological bulk tau protein concentrations. Moreover, a subset of these proteins have been previously linked to AD, including DNAJA1 and BAG2, which promote clearance of misfolded tau aggregates *in vitro* [72,83,84], HSPA8, which binds tau *in vitro* upon pharmacologic destabilization of microtubules [63], and HSP90 complex, which interacts with tau oligomers as well as monomer [85]. HSP90 interactions depend in part on the specific cochaperones complexed with it, including HSP70, AHA1, and FKBP5 [64,71,86,87]. Identification of individual family isoforms is important because the binding affinity of chaperones for human tau isoforms has been reported to be highly specific [70]. Moreover, gene products that protect against the propagation of tau pathology between neurons through changes in their activities or levels could be promising therapeutic targets. This is especially important when targeting excitatory neurons, which are intrinsically vulnerable to tau pathology [88]. Our finding that the proteostasis module was enriched in neocortical lamina of Brodmann area 9 (a tauopathy-affected region implicated in dementia, [5]) at stringencies correlating inversely with pathological involvement suggests that its function in resistance is not limited to cerebellar neurons.

Although interaction network analysis identified certain differentially expressed molecular chaperones as having hub character on grounds of degree and betweenness centrality, we found other high-ranking nodes to consist of transcriptional regulators, including *IER5* and *NEAT1*. These gene products are predicted to reside near the top of regulatory networks governing expression of molecular chaperones and other antagonists of tau aggregation and spread. *IER5* is an established regulator of molecular chaperone expression, and has been reported to mediate upregulation of chaperone proteins upon sleep deprivation [89], suggesting a link between proteostasis and sleep, both of which are disrupted in AD, and both of which promote tauopathy [90–93]. Our data show *IER5* is downregulated in PFC and upregulated in CB, in accord with the upregulation of chaperone targets and with tauopathy resistance of the CB. *NEAT1* is an architectural lncRNA which acts as structural scaffold for construction of paraspeckles that exhibit the properties of phase-separated condensates [94]. Upregulation of *NEAT1* has been reported to increase paraspeckle size and number [94], resulting in sequestration of specific ribonucleic acid particles and/or RNAs with subsequent changes in gene expression. In model systems, the consequences of *NEAT1* upregulation are diverse, with polarity of effects on neurodegeneration dependent on downstream events (reviewed in [95]). Here, in the context of resistant excitatory neurons of the CB, we predict that the decreased expression observed in LOAD CB region is neuroprotective.

In addition to identifying contrasting regional gene expression with LOAD, the RoR approach recognizes gene expression changes that are shared between resistant and vulnerable brain regions. These transcripts were categorized as Set 2 and they reflect adaptive responses to disease processes occurring in parallel in both regions. Strong enrichment of Set 2 with Braak stage correlated transcripts indicates these processes include increasing pathological tau burden. Set 2 transcripts associated with neuroinflammation derived primarily from astrocytes, microglia and endothelial cells. These data recapitulate findings of previous transcriptomic analyses that immune activation is a prominent and reproducible feature of LOAD gene expression [39]. However, RoR analysis places immune activation in the context of reactive gliosis, which appears in both CB and PFC regions owing to axonal degeneration occurring in both regions throughout disease and amyloid plaque deposition occurring in both regions late in disease. This positioning is consistent with findings in animal models that place immune activation as a downstream mediator of neurodegeneration rather than an upstream trigger of tau aggregation and spread [42,96]. It also parallels results from injury models, where inflammation influences neuronal survival but is a secondary response to neuronal damage [97]. An executioner role for immune response also aligns with the reported enrichment of LOAD risk alleles in immune gene modules [36,42,96]. Thus, while these changes are categorized as parallel, they nonetheless provide information on disease biology much like the contrasting component.

Despite most immune response gene expression being shared between CB and PFC regions, we found a minority associated with contrasting Set 1, in harmony with the modest enrichment of astrocyte- and Bergmann glia- specific transcripts detected by CSEA. For example, RoR analysis unmasked complement protein C5, a precursor component of the membrane attack complex associated with cytotoxicity (reviewed in [98]), as correlating positively with vulnerability owing to region-specific depression in CB. C5 has been reported to colocalize with amyloid plaques in LOAD [99], whereas its receptors increase in tangle bearing neurons, indicating that tangle-vulnerable neurons are competent to receive C5 as a signal [100]. RoR analysis also unmasked *CD47*, a “don’t eat me” mediator of protection against phagocytosis (reviewed in [101]) as correlating inversely with vulnerability owing to region-specific upregulation in CB (**Table C in S1 Data**). Further study will be needed to assess whether modulation of specific immune factors such as *C5* and *CD47* contribute to the ability of CB region to resist the neurophagic environment engendered by reactive gliosis and inflammation.

The ability of RoR to establish polarity also can be useful for characterizing candidate LOAD risk genes identified through genetic analysis [24–26]. Although risk loci continue to be identified, the identity of their associated genes that mediate LOAD risk is not fully characterized. Here, *PTK2B*, a risk factor for LOAD established through both GWAS and transcriptomic studies [23,26,102], was strongly prioritized and directly correlated with vulnerability to tauopathy. Consistent with this deduced polarity, increased *PTK2B* expression has been reported to associate with increased AD risk [81]. In our analysis, however, the polarity arose because *PTK2B* expression was strongly depressed in resistant CB region. We found similar results for *PILRA*, another established LOAD risk gene. These data suggest that transcriptomic studies such as ours can complement GWAS approaches by categorizing the polarity of candidate genes.

In summary, RoR analysis is a simple and transparent approach for disaggregating gene expression data based on regions of interest. As implemented here, it complements existing practice by capturing changes in gene expression selectively associated with LOAD resistance as opposed to changes associated with active tauopathy in affected regions. The approach has the potential to identify new targets for drug discovery predicated on their ability to promote resistance to disease in otherwise vulnerable neuron populations.

## Materials and methods

### Data preprocessing

Microarray data set GSE44772 [36] from the Harvard Brain Tissue Resource Center was used for all calculations (demographics summarized in **Tables A and B in S1 Data**). After data were accessed from the Gene Expression Omnibus (GEO) using the “GEOquery” package in R, gene names were updated to Human Genome Naming Consortium (HGNC) identifiers by cross-referencing the GEO Accession Rosetta Map with National Center for Biotechnology Information (NCBI) Entrez, HGNC, Genbank, and Unigene databases, using the “mygene” package in R as described previously [103]. Gene expression values were then corrected for demographic (age, sex) and sample quality (post-mortem interval, pH, RNA integrity number, preservation method and batch) covariates using the generalized least squares method [104] implemented within the R package LIMMA (Linear Models for Microarray and RNA-seq Data) [105,106]. The final preprocessed data set consisted of log2 expression intensity values for 39,280 probes (corresponding to 25,852 named genes) derived from 129 LOAD and 101 control cases. Annotated R code for these and subsequent analytical steps is provided as Supplementary information (**S1 Code**).

### Principal variation component analysis (PVCA)

PVCA was performed before and after mixed linear modeling using the “pvca” package in R (**S1 Code**). A threshold of 60% variation was used for the initial principal component analysis step [47,48]. In a second step, variance component analysis was performed to yield an estimate of the overall variation explained by each demographic or sample quality covariate. Input expression data consisted of PFC alone (pooled PFC^LOAD^ and PFC^Control^), CB alone (pooled CB^LOAD^ and CB^Control^), and the intra-individual ratios used in RoR analysis (PFC^LOAD^/CB^LOAD^ and PFC^Control^/CB^Control^). Because the ratio groups had pH and RNA-integrity numbers from each tissue, these PVCA calculations included nine covariates whereas PFC-only and CB-only analyses included seven covariates.

### Differential expression analysis

Fold change in gene expression between brain regions was calculated and compared between LOAD and control populations for each microarray probe using the topTable function in the limma package (**S1 Code**). The topTable function uses a two-sample *t*-test with pooled variances moderated by an empirical Bayes method to test for significance [106]. The *p*-value was adjusted using the Benjamini-Hochberg (BH) method to reduce false discovery rate [107,108].

### Cell-type Specific Enrichment Analysis (CSEA)

Gene lists were interrogated using the Cell-type Specific Enrichment Analysis tool from the Dougherty lab (http://genetics.wustl.edu/jdlab/csea-tool-2/, Version 1.1: updated 7/31/17). Overlap with lists of transcripts enriched in a particular cell type or region were identified by Fisher’s Exact Test with Benjamini-Hochberg correction as described previously [109].

### Gene Ontology analysis

Enriched Gene Ontology (GO) Biological Processes were identified using the online g:Profiler tool [110,111]. Gene lists were queried using the following settings: *Homo sapiens*, annotated genes only, g:SCS threshold of 0.05, numeric genes treated as Entrezgene_ACC, GO biological process. Results were downloaded and visualized using the Enrichment Map Pipeline Collection within Cytoscape version 3.7.1 [112]. Using advanced options, the node cutoffs were set to a false discovery rate *q*-value of 0.1 and a *p*-value of 0.05, the edge cutoff was set to 0.25, and the edge metric was set to Jaccard as in Johnson *et al*. [113]. Annotations for clustered gene sets were determined with the AutoAnnotate app.

### Interaction networks

Interaction networks were generated using the online NetworkAnalyst 3.0 tool (https://www.networkanalyst.ca/) [76]. The gene lists of interest were queried against the Encode Database to identify a subnetwork using the input list as seed nodes. The Simple Interaction Format files were then downloaded for visualization in Cytoscape 3.7.1.

### Gene Set Overlap analysis

This analysis was conducted using the “GeneOverlap” package in R (**S1 Code**), which compares multiple gene lists to define an intersection gene list and a union gene list for each pairwise comparison. Statistical significance was determined using a Fisher’s Exact Test on the proportion of intersection genes relative to the union gene list assuming a human genome size of 25,000 genes. A Benjamini-Hochberg adjusted *p*-value was used to address multiple hypothesis testing.

### Recombinant protein expression and purification

All recombinant human proteins were prepared through heterologous expression in *E*. *coli*. Recombinant 2N4R human tau was expressed from plasmid pT7II-2N4Rtau (Addgene #177653) in BL21CodonPlus (DE3)-RP cells (Agilent) and purified as described previously [114]. In contrast, molecular chaperones were prepared in a four-step process. First, the principal human isoform of each chaperone protein was identified using the Annotating principal splice isoforms (APPRIS) Database [115]. Once identified (**S1 Fig**), cDNAs for each isoform were isolated from a human cerebellum cDNA library (Stratagene, CytoTrap #975201) using polymerase chain reaction as described previously [116]. Third, each cDNA was ligated into the pT7C expression plasmid [114] to fuse each sequence in frame with an N-terminal polyhistidine tag. Finally, each construct was transformed into BL21CodonPlus (DE3)-RIL cells (Agilent), grown at 37°C to A_600_ = 0.8 and induced with 1 mM IPTG for 16 h at 16°C. Cells were harvested, resuspended in immobilized metal affinity chromatography (IMAC) Binding Buffer (500 mM NaCl, 20 mM Tris pH 8.0) containing 0.1% BME, 500 μM phenylmethylsulfonyl fluoride, and 10 mM Imidazole, and then lysed three times using a French Press operated at 10,500 psi. Homogenates were centrifuged (1 h at 200,000 x *g* at 4°C), after which supernatant fractions were filtered sequentially through 5 μm nylon and 0.20 μm PES syringe filters, then fractionated by IMAC as described for tau protein [114]. The resulting peak fractions for each chaperone (except DNAJB6) were then pooled and fractionated by size exclusion chromatography (**S1 Fig**). After dialysis in storage buffer, preparations were stored at -20°C until used. To avoid cold-sensitive precipitation, DNAJB6 was stored at room temperature after IMAC in the presence of 0.02% sodium azide until used. Chaperone molecular weight and purity was assessed by SDS-PAGE and Coomassie blue staining (**S1 Fig**).

### Tau aggregation assay

2N4R tau protein (1.5 μM) was aggregated (37°C for 16 h) in Assembly Buffer (100 mM NaCl, 10 mM HEPES pH 7.4, 5 mM DTT) containing protease inhibitor cocktail (Sigma, P8340) at 2% (v/v) final concentration. Aggregation was induced with octadecyl sulfate (ODS) at 50 μM final concentration. When present, chaperones or control protein ovalbumin ([117]; Sigma, A-5503) were tested at 0.5, 1.5, and 4.5 μM final concentrations, corresponding to 1:3, 1:1 and 3:1 molar ratios, respectively. To minimize carryover of imidazole and NaCl, DNAJB6 was diluted >35-fold into the aggregation assay.

After 16 h incubation, all assays were centrifuged (100,000 x *g* for 1 h at 4°C). The resulting supernatant fraction was carefully decanted whereas the pellet was resuspended in an equal volume of Assembly Buffer. All samples were then boiled for 5 min in SDS sample buffer, and then equal volumes of supernatant and pellet samples were run on SDS-PAGE (corresponding to ≤ 1 μg tau protein). The resulting proportion of soluble tau in the supernatant versus insoluble tau in the pellet was analyzed by SDS-PAGE with Coomassie Blue staining and quantified by densitometry using the ImageJ gel analyzer tool [118]. Band densities were normalized as a fraction of the sum of the supernatant and pellet for each reaction. An ordinary one-way ANOVA with Tukey’s multiple comparison correction was used to test all pairwise comparisons between aggregation conditions.

## Supporting information

S1 Code R code. Annotated R code for ratio of ratios analysis.
(PDF)

S1 Data Supporting Tables A-K. Table A: Patient Age, PMI, Sex, and Braak Stage Distribution. Table B: Sample pH, RIN, Preservation, and Batch Distribution. Table C: Differential Gene Expression Analysis of All Microarray Probes. Table D: Differential Gene Expression Analysis Filtered for Set 1 Genes. Table E: Differential Gene Expression Analysis Filtered for Set 2 Genes. Table F: Set 1 Genes in Enriched GO Categories. Table G: Set 2 Genes in Enriched GO Categories. Table H: Set 1 TF Interaction Network Nodes. Table I: Set 2 TF Interaction Network Nodes. Table J: Set 1 Genes that intersect with Reported AD GWAS Candidates. Table K: Set 2 Genes that intersect with Reported AD GWAS Candidates.
(XLSB)

S1 Fig Chaperone protein isolation. **(A)** Nine molecular chaperones identified in contrasting Gene Set 1 cloned from a CB cDNA library, expressed in *E*. *coli*, and purified by column chromatography. (**B**) SDS-polyacrylamide gel electrophoresis (6–12% acrylamide gradient) of purified chaperones listed in Panel A. Purified proteins were used in tau aggregation assays.
(TIF)

## References

[1] Jack CR Jr., Bennett DA, Blennow K, Carrillo MC, Feldman HH, Frisoni GB, et al. A/T/N: An unbiased descriptive classification scheme for Alzheimer disease biomarkers. Neurology. 2016; 87:539–47. doi: 10.1212/WNL.0000000000002923 27371494 PMC4970664

[2] West MJ, Coleman PD, Flood DG, Troncoso JC. Differences in the pattern of hippocampal neuronal loss in normal ageing and Alzheimer’s disease. Lancet. 1994; 344:769–72. doi: 10.1016/s0140-6736(94)92338-8 7916070

[3] Gomez-Isla T, Price JL, McKeel DW Jr., Morris JC, Growdon JH, Hyman BT. Profound loss of layer II entorhinal cortex neurons occurs in very mild Alzheimer’s disease. J Neurosci. 1996; 16:4491–500. doi: 10.1523/JNEUROSCI.16-14-04491.1996 8699259 PMC6578866

[4] Simic G, Babic Leko M, Wray S, Harrington C, Delalle I, Jovanov-Milosevic N, et al. Tau Protein Hyperphosphorylation and Aggregation in Alzheimer’s Disease and Other Tauopathies, and Possible Neuroprotective Strategies. Biomolecules. 2016; 6:6. doi: 10.3390/biom6010006 26751493 PMC4808800

[5] Royall DR, Palmer R, Mulroy AR, Polk MJ, Roman GC, David JP, et al. Pathological determinants of the transition to clinical dementia in Alzheimer’s disease. Exp Aging Res. 2002; 28:143–62. doi: 10.1080/03610730252800166 11928525

[6] Braak H, Thal DR, Ghebremedhin E, Del Tredici K. Stages of the pathologic process in Alzheimer disease: age categories from 1 to 100 years. J Neuropathol Exp Neurol. 2011; 70:960–9. doi: 10.1097/NEN.0b013e318232a379 22002422

[7] Braak H, Del Tredici K. The pathological process underlying Alzheimer’s disease in individuals under thirty. Acta Neuropathol. 2011; 121:171–81. doi: 10.1007/s00401-010-0789-4 21170538

[8] Prusiner SB. Biology and genetics of prions causing neurodegeneration. Annu Rev Genet. 2013; 47:601–23. doi: 10.1146/annurev-genet-110711-155524 24274755 PMC4010318

[9] Hyman BT. Tau propagation, different tau phenotypes, and prion-like properties of tau. Neuron. 2014; 82:1189–90. doi: 10.1016/j.neuron.2014.06.004 24945760

[10] Braak H, Del Tredici K. Alzheimer’s pathogenesis: is there neuron-to-neuron propagation? Acta Neuropathol. 2011; 121:589–95. doi: 10.1007/s00401-011-0825-z 21516512

[11] Larner AJ. The cerebellum in Alzheimer’s disease. Dement Geriatr Cogn Disord. 1997; 8:203–9. doi: 10.1159/000106632 9213064

[12] Braak H, Braak E, Bohl J, Lang W. Alzheimer’s disease: amyloid plaques in the cerebellum. J Neurol Sci. 1989; 93:277–87. doi: 10.1016/0022-510x(89)90197-4 2556503

[13] Andersen K, Andersen BB, Pakkenberg B. Stereological quantification of the cerebellum in patients with Alzheimer’s disease. Neurobiol Aging. 2012; 33:197 e11–20. doi: 10.1016/j.neurobiolaging.2010.06.013 20728248

[14] Benarroch EE. Locus coeruleus. Cell Tissue Res. 2018; 373:221–32. doi: 10.1007/s00441-017-2649-1 28687925

[15] Guo CC, Tan R, Hodges JR, Hu X, Sami S, Hornberger M. Network-selective vulnerability of the human cerebellum to Alzheimer’s disease and frontotemporal dementia. Brain. 2016; 139:1527–38. doi: 10.1093/brain/aww003 26912642 PMC5839595

[16] Furman JL, Vaquer-Alicea J, White CL 3rd, Cairns NJ, Nelson PT, Diamond MI. Widespread tau seeding activity at early Braak stages. Acta Neuropathol. 2017; 133:91–100. doi: 10.1007/s00401-016-1644-z 27878366 PMC5833300

[17] Thal DR, Rub U, Orantes M, Braak H. Phases of Aβ-deposition in the human brain and its relevance for the development of AD. Neurology. 2002; 58:1791–800. doi: 10.1212/wnl.58.12.1791 12084879

[18] Schmahmann JD. Cerebellum in Alzheimer’s disease and frontotemporal dementia: not a silent bystander. Brain. 2016; 139:1314–8. doi: 10.1093/brain/aww064 27189578

[19] Sepulveda-Falla D, Matschke J, Bernreuther C, Hagel C, Puig B, Villegas A, et al. Deposition of Hyperphosphorylated Tau in Cerebellum of PS1 E280A Alzheimer’s Disease. Brain Pathol. 2011; 21:452–63. doi: 10.1111/j.1750-3639.2010.00469.x 21159009 PMC8094246

[20] Arenaza-Urquijo EM, Vemuri P. Resistance vs resilience to Alzheimer disease: Clarifying terminology for preclinical studies. Neurology. 2018; 90:695–703. doi: 10.1212/WNL.0000000000005303 29592885 PMC5894932

[21] Chappell S, Patel T, Guetta-Baranes T, Sang F, Francis PT, Morgan K, et al. Observations of extensive gene expression differences in the cerebellum and potential relevance to Alzheimer’s disease. BMC Res Notes. 2018; 11:646. doi: 10.1186/s13104-018-3732-8 30180886 PMC6123947

[22] Xu J, Patassini S, Rustogi N, Riba-Garcia I, Hale BD, Phillips AM, et al. Regional protein expression in human Alzheimer’s brain correlates with disease severity. Commun Biol. 2019; 2:43. doi: 10.1038/s42003-018-0254-9 30729181 PMC6361956

[23] Lambert JC, Ibrahim-Verbaas CA, Harold D, Naj AC, Sims R, Bellenguez C, et al. Meta-analysis of 74,046 individuals identifies 11 new susceptibility loci for Alzheimer’s disease. Nat Genet. 2013; 45:1452–8. doi: 10.1038/ng.2802 24162737 PMC3896259

[24] Bertram L, Tanzi RE. Genomic mechanisms in Alzheimer’s disease. Brain Pathol. 2020; 30:966–77. doi: 10.1111/bpa.12882 32657454 PMC8018017

[25] Jansen IE, Savage JE, Watanabe K, Bryois J, Williams DM, Steinberg S, et al. Genome-wide meta-analysis identifies new loci and functional pathways influencing Alzheimer’s disease risk. Nat Genet. 2019; 51:404–13. doi: 10.1038/s41588-018-0311-9 30617256 PMC6836675

[26] Kunkle BW, Grenier-Boley B, Sims R, Bis JC, Damotte V, Naj AC, et al. Genetic meta-analysis of diagnosed Alzheimer’s disease identifies new risk loci and implicates Abeta, tau, immunity and lipid processing. Nat Genet. 2019; 51:414–30. doi: 10.1038/s41588-019-0358-2 30820047 PMC6463297

[27] Blalock EM, Geddes JW, Chen KC, Porter NM, Markesbery WR, Landfield PW. Incipient Alzheimer’s disease: microarray correlation analyses reveal major transcriptional and tumor suppressor responses. Proc Natl Acad Sci U S A. 2004; 101:2173–8. doi: 10.1073/pnas.0308512100 14769913 PMC357071

[28] Colangelo V, Schurr J, Ball MJ, Pelaez RP, Bazan NG, Lukiw WJ. Gene expression profiling of 12633 genes in Alzheimer hippocampal CA1: transcription and neurotrophic factor down-regulation and up-regulation of apoptotic and pro-inflammatory signaling. J Neurosci Res. 2002; 70:462–73. doi: 10.1002/jnr.10351 12391607

[29] Emilsson L, Saetre P, Jazin E. Alzheimer’s disease: mRNA expression profiles of multiple patients show alterations of genes involved with calcium signaling. Neurobiol Dis. 2006; 21:618–25. doi: 10.1016/j.nbd.2005.09.004 16257224

[30] Fowler KD, Funt JM, Artyomov MN, Zeskind B, Kolitz SE, Towfic F. Leveraging existing data sets to generate new insights into Alzheimer’s disease biology in specific patient subsets. Sci Rep. 2015; 5:14324. doi: 10.1038/srep14324 26395074 PMC4585817

[31] Gleichmann M, Zhang Y, Wood WH 3rd, Becker KG, Mughal MR, Pazin MJ, et al. Molecular changes in brain aging and Alzheimer’s disease are mirrored in experimentally silenced cortical neuron networks. Neurobiol Aging. 2012; 33:205 e1–18. doi: 10.1016/j.neurobiolaging.2010.08.012 20947216 PMC3027841

[32] Ho L, Guo Y, Spielman L, Petrescu O, Haroutunian V, Purohit D, et al. Altered expression of a-type but not b-type synapsin isoform in the brain of patients at high risk for Alzheimer’s disease assessed by DNA microarray technique. Neurosci Lett. 2001; 298:191–4. doi: 10.1016/s0304-3940(00)01753-5 11165439

[33] Tan MG, Chua WT, Esiri MM, Smith AD, Vinters HV, Lai MK. Genome wide profiling of altered gene expression in the neocortex of Alzheimer’s disease. J Neurosci Res. 2010; 88:1157–69. doi: 10.1002/jnr.22290 19937809

[34] Webster JA, Gibbs JR, Clarke J, Ray M, Zhang W, Holmans P, et al. Genetic control of human brain transcript expression in Alzheimer disease. Am J Hum Genet. 2009; 84:445–58. doi: 10.1016/j.ajhg.2009.03.011 19361613 PMC2667989

[35] Weeraratna AT, Kalehua A, Deleon I, Bertak D, Maher G, Wade MS, et al. Alterations in immunological and neurological gene expression patterns in Alzheimer’s disease tissues. Exp Cell Res. 2007; 313:450–61. doi: 10.1016/j.yexcr.2006.10.028 17188679 PMC2565515

[36] Zhang B, Gaiteri C, Bodea LG, Wang Z, McElwee J, Podtelezhnikov AA, et al. Integrated systems approach identifies genetic nodes and networks in late-onset Alzheimer’s disease. Cell. 2013; 153:707–20. doi: 10.1016/j.cell.2013.03.030 23622250 PMC3677161

[37] Morabito S, Miyoshi E, Michael N, Swarup V. Integrative genomics approach identifies conserved transcriptomic networks in Alzheimer’s disease. Hum Mol Genet. 2020; 29:2899–919. doi: 10.1093/hmg/ddaa182 32803238 PMC7566321

[38] Rexach JE, Polioudakis D, Yin A, Swarup V, Chang TS, Nguyen T, et al. Tau Pathology Drives Dementia Risk-Associated Gene Networks toward Chronic Inflammatory States and Immunosuppression. Cell Rep. 2020; 33:108398. doi: 10.1016/j.celrep.2020.108398 33207193 PMC7842189

[39] Wang M, Roussos P, McKenzie A, Zhou X, Kajiwara Y, Brennand KJ, et al. Integrative network analysis of nineteen brain regions identifies molecular signatures and networks underlying selective regional vulnerability to Alzheimer’s disease. Genome Med. 2016; 8:104. doi: 10.1186/s13073-016-0355-3 27799057 PMC5088659

[40] Wang M, Li A, Sekiya M, Beckmann ND, Quan X, Schrode N, et al. Transformative Network Modeling of Multi-omics Data Reveals Detailed Circuits, Key Regulators, and Potential Therapeutics for Alzheimer’s Disease. Neuron. 2021; 109:257–72 e14. doi: 10.1016/j.neuron.2020.11.002 33238137 PMC7855384

[41] Piras IS, Krate J, Delvaux E, Nolz J, Mastroeni DF, Persico AM, et al. Transcriptome Changes in the Alzheimer’s Disease Middle Temporal Gyrus: Importance of RNA Metabolism and Mitochondria-Associated Membrane Genes. J Alzheimers Dis. 2019; 70:691–713. doi: 10.3233/JAD-181113 31256118

[42] Wan YW, Al-Ouran R, Mangleburg CG, Perumal TM, Lee TV, Allison K, et al. Meta-Analysis of the Alzheimer’s Disease Human Brain Transcriptome and Functional Dissection in Mouse Models. Cell Rep. 2020; 32:107908. doi: 10.1016/j.celrep.2020.107908 32668255 PMC7428328

[43] Livak KJ, Schmittgen TD. Analysis of relative gene expression data using real-time quantitative PCR and the 2(-Delta Delta C(T)) Method. Methods. 2001; 25:402–8. doi: 10.1006/meth.2001.1262 11846609

[44] Schmittgen TD, Livak KJ. Analyzing real-time PCR data by the comparative C(T) method. Nat Protoc. 2008; 3:1101–8. doi: 10.1038/nprot.2008.73 18546601

[45] Aoyagi T. Pulse oximetry: its invention, theory, and future. J Anesth. 2003; 17:259–66. doi: 10.1007/s00540-003-0192-6 14625714

[46] Lazic SE. Ranking, selecting, and prioritising genes with desirability functions. PeerJ. 2015; 3:e1444. doi: 10.7717/peerj.1444 26644980 PMC4671156

[47] Li J, Bushel PR, Chu T-M, Wolfinger RD. Principal variance components analysis: Estimating batch effects in microarray gene expression data. In: Scherer A, editor. Batch effects and noise in microarray experiments Sources and solutions. Wiley Series in probability and statistics. West Sussex, U.K.: John Wiley & Sons; 2009. p. 141–54.

[48] Chen C, Grennan K, Badner J, Zhang D, Gershon E, Jin L, et al. Removing batch effects in analysis of expression microarray data: an evaluation of six batch adjustment methods. PLoS One. 2011; 6:e17238. doi: 10.1371/journal.pone.0017238 21386892 PMC3046121

[49] McKenzie AT, Wang M, Hauberg ME, Fullard JF, Kozlenkov A, Keenan A, et al. Brain Cell Type Specific Gene Expression and Co-expression Network Architectures. Sci Rep. 2018; 8:8868. doi: 10.1038/s41598-018-27293-5 29892006 PMC5995803

[50] Dougherty JD, Schmidt EF, Nakajima M, Heintz N. Analytical approaches to RNA profiling data for the identification of genes enriched in specific cells. Nucleic Acids Res. 2010; 38:4218–30. doi: 10.1093/nar/gkq130 20308160 PMC2910036

[51] Xu X, Wells AB, O’Brien DR, Nehorai A, Dougherty JD. Cell type-specific expression analysis to identify putative cellular mechanisms for neurogenetic disorders. J Neurosci. 2014; 34:1420–31. doi: 10.1523/JNEUROSCI.4488-13.2014 24453331 PMC3898298

[52] Doyle JP, Dougherty JD, Heiman M, Schmidt EF, Stevens TR, Ma G, et al. Application of a translational profiling approach for the comparative analysis of CNS cell types. Cell. 2008; 135:749–62. doi: 10.1016/j.cell.2008.10.029 19013282 PMC2763427

[53] DeTure MA, Dickson DW. The neuropathological diagnosis of Alzheimer’s disease. Mol Neurodegener. 2019; 14:32. doi: 10.1186/s13024-019-0333-5 31375134 PMC6679484

[54] Bussiere T, Gold G, Kovari E, Giannakopoulos P, Bouras C, Perl DP, et al. Stereologic analysis of neurofibrillary tangle formation in prefrontal cortex area 9 in aging and Alzheimer’s disease. Neuroscience. 2003; 117:577–92. doi: 10.1016/s0306-4522(02)00942-9 12617964

[55] De Zeeuw CI, Hoogland TM. Reappraisal of Bergmann glial cells as modulators of cerebellar circuit function. Front Cell Neurosci. 2015; 9:246. doi: 10.3389/fncel.2015.00246 26190972 PMC4488625

[56] Subramanian A, Tamayo P, Mootha VK, Mukherjee S, Ebert BL, Gillette MA, et al. Gene set enrichment analysis: a knowledge-based approach for interpreting genome-wide expression profiles. Proc Natl Acad Sci U S A. 2005; 102:15545–50. doi: 10.1073/pnas.0506580102 16199517 PMC1239896

[57] The Gene Ontology C. The Gene Ontology Resource: 20 years and still GOing strong. Nucleic Acids Res. 2019; 47:D330–D8. doi: 10.1093/nar/gky1055 30395331 PMC6323945

[58] Ashburner M, Ball CA, Blake JA, Botstein D, Butler H, Cherry JM, et al. Gene ontology: tool for the unification of biology. The Gene Ontology Consortium. Nat Genet. 2000; 25:25–9. doi: 10.1038/75556 10802651 PMC3037419

[59] Kinney JW, Bemiller SM, Murtishaw AS, Leisgang AM, Salazar AM, Lamb BT. Inflammation as a central mechanism in Alzheimer’s disease. Alzheimers Dement (N Y). 2018; 4:575–90. doi: 10.1016/j.trci.2018.06.014 30406177 PMC6214864

[60] Irwin R, Faust O, Petrovic I, Wolf SG, Hofmann H, Rosenzweig R. Hsp40s play complementary roles in the prevention of tau amyloid formation. Elife. 2021; 10. doi: 10.7554/eLife.69601 34369377 PMC8437434

[61] Nachman E, Wentink AS, Madiona K, Bousset L, Katsinelos T, Allinson K, et al. Disassembly of Tau fibrils by the human Hsp70 disaggregation machinery generates small seeding-competent species. J Biol Chem. 2020; 295:9676–90. doi: 10.1074/jbc.RA120.013478 32467226 PMC7363153

[62] Kundel F, De S, Flagmeier P, Horrocks MH, Kjaergaard M, Shammas SL, et al. Hsp70 Inhibits the Nucleation and Elongation of Tau and Sequesters Tau Aggregates with High Affinity. Acs Chem Biol. 2018; 13:636–46. doi: 10.1021/acschembio.7b01039 29300447 PMC6374916

[63] Jinwal UK, O’Leary JC 3rd, Borysov SI, Jones JR, Li Q, Koren J 3rd, et al. Hsc70 rapidly engages tau after microtubule destabilization. J Biol Chem. 2010; 285:16798–805. doi: 10.1074/jbc.M110.113753 20308058 PMC2878041

[64] Karagoz GE, Duarte AM, Akoury E, Ippel H, Biernat J, Moran Luengo T, et al. Hsp90-Tau complex reveals molecular basis for specificity in chaperone action. Cell. 2014; 156:963–74. doi: 10.1016/j.cell.2014.01.037 24581495 PMC4263503

[65] Jinwal UK, Akoury E, Abisambra JF, O’Leary JC 3rd, Thompson AD, Blair LJ, et al. Imbalance of Hsp70 family variants fosters tau accumulation. FASEB J. 2013; 27:1450–9. doi: 10.1096/fj.12-220889 23271055 PMC3606536

[66] Voss K, Combs B, Patterson KR, Binder LI, Gamblin TC. Hsp70 alters tau function and aggregation in an isoform specific manner. Biochemistry. 2012; 51:888–98. doi: 10.1021/bi2018078 22236337 PMC3278803

[67] Gao X, Carroni M, Nussbaum-Krammer C, Mogk A, Nillegoda NB, Szlachcic A, et al. Human Hsp70 Disaggregase Reverses Parkinson’s-Linked alpha-Synuclein Amyloid Fibrils. Mol Cell. 2015; 59:781–93. doi: 10.1016/j.molcel.2015.07.012 26300264 PMC5072489

[68] Hageman J, Kampinga HH. Computational analysis of the human HSPH/HSPA/DNAJ family and cloning of a human HSPH/HSPA/DNAJ expression library. Cell Stress Chaperones. 2009; 14:1–21. doi: 10.1007/s12192-008-0060-2 18686016 PMC2673897

[69] Eroglu B, Moskophidis D, Mivechi NF. Loss of Hsp110 leads to age-dependent tau hyperphosphorylation and early accumulation of insoluble amyloid beta. Mol Cell Biol. 2010; 30:4626–43. doi: 10.1128/MCB.01493-09 20679486 PMC2950521

[70] Mok SA, Condello C, Freilich R, Gillies A, Arhar T, Oroz J, et al. Mapping interactions with the chaperone network reveals factors that protect against tau aggregation. Nat Struct Mol Biol. 2018; 25:384–93. doi: 10.1038/s41594-018-0057-1 29728653 PMC5942583

[71] Thompson AD, Scaglione KM, Prensner J, Gillies AT, Chinnaiyan A, Paulson HL, et al. Analysis of the Tau-Associated Proteome Reveals That Exchange of Hsp70 for Hsp90 Is Involved in Tau Degradation. Acs Chem Biol. 2012; 7:1677–86. doi: 10.1021/cb3002599 22769591 PMC3477299

[72] Abisambra JF, Jinwal UK, Suntharalingam A, Arulselvam K, Brady S, Cockman M, et al. DnaJA1 antagonizes constitutive Hsp70-mediated stabilization of tau. J Mol Biol. 2012; 421:653–61. doi: 10.1016/j.jmb.2012.02.003 22343013 PMC3371317

[73] Petrucelli L, Dickson D, Kehoe K, Taylor J, Snyder H, Grover A, et al. CHIP and Hsp70 regulate tau ubiquitination, degradation and aggregation. Hum Mol Genet. 2004; 13:703–14. doi: 10.1093/hmg/ddh083 14962978

[74] Chirita CN, Necula M, Kuret J. Anionic micelles and vesicles induce tau fibrillization in vitro. J Biol Chem. 2003; 278:25644–50. doi: 10.1074/jbc.M301663200 12730214

[75] Goedert M, Spillantini MG, Jakes R, Rutherford D, Crowther RA. Multiple isoforms of human microtubule-associated protein tau: sequences and localization in neurofibrillary tangles of Alzheimer’s disease. Neuron. 1989; 3:519–26. doi: 10.1016/0896-6273(89)90210-9 2484340

[76] Zhou G, Soufan O, Ewald J, Hancock REW, Basu N, Xia J. NetworkAnalyst 3.0: a visual analytics platform for comprehensive gene expression profiling and meta-analysis. Nucleic Acids Res. 2019; 47:W234–W41. doi: 10.1093/nar/gkz240 30931480 PMC6602507

[77] Zhang Y, Sun Z, Jia J, Du T, Zhang N, Tang Y, et al. Overview of Histone Modification. Adv Exp Med Biol. 2021; 1283:1–16. doi: 10.1007/978-981-15-8104-5_1 33155134

[78] Kim JR, Lee SR, Chung HJ, Kim S, Baek SH, Kim JH, et al. Identification of amyloid beta-peptide responsive genes by cDNA microarray technology: involvement of RTP801 in amyloid beta-peptide toxicity. Exp Mol Med. 2003; 35:403–11. doi: 10.1038/emm.2003.53 14646594

[79] Morel M, Couturier J, Pontcharraud R, Gil R, Fauconneau B, Paccalin M, et al. Evidence of molecular links between PKR and mTOR signalling pathways in Abeta neurotoxicity: role of p53, Redd1 and TSC2. Neurobiol Dis. 2009; 36:151–61. doi: 10.1016/j.nbd.2009.07.004 19631745

[80] Wightman DP, Jansen IE, Savage JE, Shadrin AA, Bahrami S, Holland D, et al. A genome-wide association study with 1,126,563 individuals identifies new risk loci for Alzheimer’s disease. Nat Genet. 2021; 53:1276–82. doi: 10.1038/s41588-021-00921-z 34493870 PMC10243600

[81] Novikova G, Kapoor M, Tcw J, Abud EM, Efthymiou AG, Chen SX, et al. Integration of Alzheimer’s disease genetics and myeloid genomics identifies disease risk regulatory elements and genes. Nature communications. 2021; 12:1610. doi: 10.1038/s41467-021-21823-y 33712570 PMC7955030

[82] Freer R, Sormanni P, Vecchi G, Ciryam P, Dobson CM, Vendruscolo M. A protein homeostasis signature in healthy brains recapitulates tissue vulnerability to Alzheimer’s disease. Sci Adv. 2016; 2:e1600947. doi: 10.1126/sciadv.1600947 27532054 PMC4980108

[83] Carrettiero DC, Hernandez I, Neveu P, Papagiannakopoulos T, Kosik KS. The cochaperone BAG2 sweeps paired helical filament- insoluble tau from the microtubule. J Neurosci. 2009; 29:2151–61. doi: 10.1523/JNEUROSCI.4660-08.2009 19228967 PMC2768429

[84] de Paula CA, Santiago FE, de Oliveira AS, Oliveira FA, Almeida MC, Carrettiero DC. The Co-chaperone BAG2 Mediates Cold-Induced Accumulation of Phosphorylated Tau in SH-SY5Y Cells. Cell Mol Neurobiol. 2016; 36:593–602. doi: 10.1007/s10571-015-0239-x 26208804 PMC11482429

[85] Blair LJ, Nordhues BA, Hil SE, Scaglione M, O’Leary JC, Fontaine SN, et al. Accelerated neurodegeneration through chaperone-mediated oligomerization of tau. J Clin Invest. 2013; 123:4158–69. doi: 10.1172/JCI69003 23999428 PMC3784538

[86] Tortosa E, Santa-Maria I, Moreno F, Lim F, Perez M, Avila J. Binding of Hsp90 to tau promotes a conformational change and aggregation of tau protein. J Alzheimers Dis. 2009; 17:319–25. doi: 10.3233/JAD-2009-1049 19363271

[87] Shelton LB, Baker JD, Zheng DL, Sullivan LE, Solanki PK, Webster JM, et al. Hsp90 activator Aha1 drives production of pathological tau aggregates. P Natl Acad Sci USA. 2017; 114:9707–12. doi: 10.1073/pnas.1707039114 28827321 PMC5594679

[88] Fu H, Possenti A, Freer R, Nakano Y, Hernandez Villegas NC, Tang M, et al. A tau homeostasis signature is linked with the cellular and regional vulnerability of excitatory neurons to tau pathology. Nat Neurosci. 2019; 22:47–56. doi: 10.1038/s41593-018-0298-7 30559469 PMC6330709

[89] Cirelli C, Tononi G. Gene expression in the brain across the sleep-waking cycle. Brain Res. 2000; 885:303–21. doi: 10.1016/s0006-8993(00)03008-0 11102586

[90] Brzecka A, Leszek J, Ashraf GM, Ejma M, Avila-Rodriguez MF, Yarla NS, et al. Sleep Disorders Associated With Alzheimer’s Disease: A Perspective. Front Neurosci. 2018; 12:330. doi: 10.3389/fnins.2018.00330 29904334 PMC5990625

[91] Holth JK, Fritschi SK, Wang C, Pedersen NP, Cirrito JR, Mahan TE, et al. The sleep-wake cycle regulates brain interstitial fluid tau in mice and CSF tau in humans. Science. 2019; 363:880–4. doi: 10.1126/science.aav2546 30679382 PMC6410369

[92] Ju YE, Lucey BP, Holtzman DM. Sleep and Alzheimer disease pathology—a bidirectional relationship. Nat Rev Neurol. 2014; 10:115–9. doi: 10.1038/nrneurol.2013.269 24366271 PMC3979317

[93] Xie L, Kang H, Xu Q, Chen MJ, Liao Y, Thiyagarajan M, et al. Sleep drives metabolite clearance from the adult brain. Science. 2013; 342:373–7. doi: 10.1126/science.1241224 24136970 PMC3880190

[94] Yamazaki T, Souquere S, Chujo T, Kobelke S, Chong YS, Fox AH, et al. Functional Domains of NEAT1 Architectural lncRNA Induce Paraspeckle Assembly through Phase Separation. Mol Cell. 2018; 70:1038–53 e7. doi: 10.1016/j.molcel.2018.05.019 29932899

[95] Prinz F, Kapeller A, Pichler M, Klec C. The Implications of the Long Non-Coding RNA NEAT1 in Non-Cancerous Diseases. International journal of molecular sciences. 2019; 20. doi: 10.3390/ijms20030627 30717168 PMC6387324

[96] Swarup V, Hinz FI, Rexach JE, Noguchi KI, Toyoshiba H, Oda A, et al. Identification of evolutionarily conserved gene networks mediating neurodegenerative dementia. Nat Med. 2019; 25:152–64. doi: 10.1038/s41591-018-0223-3 30510257 PMC6602064

[97] Donnelly DJ, Popovich PG. Inflammation and its role in neuroprotection, axonal regeneration and functional recovery after spinal cord injury. Exp Neurol. 2008; 209:378–88. doi: 10.1016/j.expneurol.2007.06.009 17662717 PMC2692462

[98] Krance SH, Wu CY, Zou Y, Mao H, Toufighi S, He X, et al. The complement cascade in Alzheimer’s disease: a systematic review and meta-analysis. Mol Psychiatry. 2021; 26:5532–41. doi: 10.1038/s41380-019-0536-8 31628417

[99] Xiong F, Ge W, Ma C. Quantitative proteomics reveals distinct composition of amyloid plaques in Alzheimer’s disease. Alzheimers Dement. 2019; 15:429–40. doi: 10.1016/j.jalz.2018.10.006 30502339

[100] Fonseca MI, McGuire SO, Counts SE, Tenner AJ. Complement activation fragment C5a receptors, CD88 and C5L2, are associated with neurofibrillary pathology. J Neuroinflammation. 2013; 10:25. doi: 10.1186/1742-2094-10-25 23394121 PMC3605123

[101] Kelley SM, Ravichandran KS. Putting the brakes on phagocytosis: "don’t-eat-me" signaling in physiology and disease. EMBO reports. 2021; 22:e52564. doi: 10.15252/embr.202152564 34041845 PMC8183410

[102] Verheijen J, Sleegers K. Understanding Alzheimer Disease at the Interface between Genetics and Transcriptomics. Trends Genet. 2018; 34:434–47. doi: 10.1016/j.tig.2018.02.007 29573818

[103] Xin J, Mark A, Afrasiabi C, Tsueng G, Juchler M, Gopal N, et al. High-performance web services for querying gene and variant annotation. Genome Biol. 2016; 17:91. doi: 10.1186/s13059-016-0953-9 27154141 PMC4858870

[104] Aitken AC. IV.—On Least Squares and Linear Combination of Observations. Proceedings of the Royal Society of Edinburgh. 2014; 55:42–8. 10.1017/s0370164600014346

[105] Ritchie ME, Phipson B, Wu D, Hu Y, Law CW, Shi W, et al. limma powers differential expression analyses for RNA-sequencing and microarray studies. Nucleic Acids Res. 2015; 43:e47. doi: 10.1093/nar/gkv007 25605792 PMC4402510

[106] Smyth GK. Linear models and empirical bayes methods for assessing differential expression in microarray experiments. Stat Appl Genet Mol Biol. 2004; 3:Article3. doi: 10.2202/1544-6115.1027 16646809

[107] Benjamini Y, Hochberg Y. Controlling the false discovery rate: A practical and powerful approach to multiple testing. J Royal Statistical Society, Ser B. 1995; 57:289–300. WOS:A1995QE45300017

[108] Benjamini Y, Yekutieli D. The control of the false discovery rate in multiple testing under dependency. Ann Stat. 2001; 29:1165–88. WOS:000172838100012

[109] Xu X, Nehorai A, Dougherty J. Cell Type Specific Analysis of Human Brain Transcriptome Data to Predict Alterations in Cellular Composition. Syst Biomed (Austin). 2013; 1:151–60. doi: 10.4161/sysb.25630 25340014 PMC4203443

[110] Raudvere U, Kolberg L, Kuzmin I, Arak T, Adler P, Peterson H, et al. g:Profiler: a web server for functional enrichment analysis and conversions of gene lists (2019 update). Nucleic Acids Res. 2019; 47:W191–W8. doi: 10.1093/nar/gkz369 31066453 PMC6602461

[111] Reimand J, Arak T, Vilo J. g:Profiler—a web server for functional interpretation of gene lists (2011 update). Nucleic Acids Res. 2011; 39:W307–15. doi: 10.1093/nar/gkr378 21646343 PMC3125778

[112] Merico D, Isserlin R, Stueker O, Emili A, Bader GD. Enrichment map: a network-based method for gene-set enrichment visualization and interpretation. Plos One. 2010; 5:e13984. doi: 10.1371/journal.pone.0013984 21085593 PMC2981572

[113] Johnson EK, Matkovich SJ, Nerbonne JM. Regional Differences in mRNA and lncRNA Expression Profiles in Non-Failing Human Atria and Ventricles. Sci Rep. 2018; 8:13919. doi: 10.1038/s41598-018-32154-2 30224797 PMC6141608

[114] Carmel G, Mager EM, Binder LI, Kuret J. The structural basis of monoclonal antibody Alz50’s selectivity for Alzheimer’s disease pathology. J Biol Chem. 1996; 271:32789–95. doi: 10.1074/jbc.271.51.32789 8955115

[115] Rodriguez JM, Rodriguez-Rivas J, Di Domenico T, Vazquez J, Valencia A, Tress ML. APPRIS 2017: principal isoforms for multiple gene sets. Nucleic Acids Res. 2018; 46:D213–D7. doi: 10.1093/nar/gkx997 29069475 PMC5753224

[116] Yin H, Laguna KA, Li G, Kuret J. Dysbindin structural homologue CK1BP is an isoform-selective binding partner of human casein kinase-1. Biochemistry. 2006; 45:5297–308. doi: 10.1021/bi052354e 16618118

[117] Shimizu T, Korehisa T, Imanaka H, Ishida N, Imamura K. Characteristics of proteinaceous additives in stabilizing enzymes during freeze-thawing and -drying. Biosci Biotechnol Biochem. 2017; 81:687–97. doi: 10.1080/09168451.2016.1274637 28067593

[118] Schneider CA, Rasband WS, Eliceiri KW. NIH Image to ImageJ: 25 years of image analysis. Nat Methods. 2012; 9:671–5. doi: 10.1038/nmeth.2089 22930834 PMC5554542

