# Supplementary material for: Identification of gene networks mediating regional resistance to tauopathy in late-onset Alzheimer’s disease
Source: PLoS Genet. 2023 Mar 27;19(3):e1010681. doi: 10.1371/journal.pgen.1010681 (PMC10079065; doi:10.1371/journal.pgen.1010681)
Supplement: S1 Code — Annotated R code for ratio of ratios analysis. (PDF) [file pgen.1010681.s001.pdf]

S1 Code

Christopher Ayoub

12/09/2022

Contents

Setting up workspace **2**

Data preparation **2**
Downloading the data . . . 2
Cleaning the demographic and sample QC data . . . 2
Reading in pathology data . . . 4
Merging pathology data with other patient-wise covariates . . . 4
Merging sample-wise and patient-wise covariates . . . 6
Summary tables of sample-wise and patient-wise covariates (S1 and S2 Tables) . . . 7
Assessing expression distribution across brain regions . . . 8
Transforming expression data from log10 to log2 . . . 11
Building new expression objects . . . 11

Covariate correction by linear modeling **12**
Limma without contrast for PVCA . . . 12
Single plot in ggplot to summarize PVCA results (Fig 2) . . . 18

Differential expression **20**
Limma with contrast matrix for TopTable . . . 20

Prioritization Scheme (Fig 3A, Fig 3B) **22**

Overlap with previous publications **25**
Overlap with Zhang et. al Braak-stage correlated genes (Fig 3C) . . . 26
Overlap with McKenzie et al. cell-type specific marker genes (Fig 4) . . . 27

Plotting specific gene sets **29**
Heat Shock Set 1 Genes (Fig 7A) . . . 29
Immune Set 2 Genes (Fig 7B) . . . 30

Responding to Reviewers **33**
Comparing logRoR to "logFC alternative" . . . 33
Distribution of Braak-correlated genes on Prioritization Curve . . . 35
How does desirability sort? . . . 42
Comparison to MayoSeq Dataset . . . 42

Session information for reproducibility **49**

Setting up workspace

```
library(Biobase)
library(GEOquery)
library(dplyr)
library(tibble)
library(genefilter)
library(edgeR)
library(R.utils)
library(report)
library(ExpressionNormalizationWorkflow)
library(tidyr)
library(mygene)
library(ggplot2)
library(GeneOverlap)
library(readxl)
library(writexl)
library(knitr)
```

Data preparation

Downloading the data

```
# Downloading GSE44772, which includes PFC, CB, and VC
Sys.setenv("VROOM_CONNECTION_SIZE" = 131072*20)
gse44772 <- getGEO("GSE44772", destdir = getwd(), GSEMatrix = TRUE, AnnotGPL = TRUE)
```

```
## Found 1 file(s)

## GSE44772_series_matrix.txt.gz

## Using locally cached version:

S:/CAA/Gene Expression/GSE44772 Manuscript/R/GSE44772_series_matrix.txt.gz

## Rows: 39280 Columns: 691
## -- Column specification --------------------------------------------------------
## Delimiter: "\t"
## dbl (691): ID_REF, GSM1090267, GSM1090268, GSM1090269, GSM1090270, GSM109027...
##
## i Use `spec()` to retrieve the full column specification for this data.
## i Specify the column types or set `show_col_types = FALSE` to quiet this message.
## Using locally cached version of GPL4372 found here:
## S:/CAA/Gene Expression/GSE44772 Manuscript/R/GPL4372.annot.gz

## Warning: One or more parsing issues, call `problems()` on your data frame for details,
## e.g.:
##   dat <- vroom(...)
##   problems(dat)
```

```
gse44772 <- gse44772[[1]]
```

Cleaning the demographic and sample QC data

```
# Renaming columns and dropping excess columns
samples <- pData(gse44772) %>%
```

```
  dplyr::select(
    c(
      Diagnosis = 'disease:ch2',
      PMI = 'pmi:ch2',
      Age = 'age:ch2',
      RIN = 'rin:ch2',
      Preservation = 'pres:ch2',
      Batch = 'batch:ch2',
      Sex = 'gender:ch2',
      pH = 'ph:ch2',
      title,
      geo_accession
    )
  ) %>%
  separate(
    col = title,
    into = c('BrainID', 'BrainRegion'),
    sep = "_"
  ) %>% ## Split 'title' into 'BrainID' and 'BrainRegion'
  mutate(BrainRegion = recode(BrainRegion, "CR" = "CB")) %>% ## Rename CR to CB for
  ↪  consistency
  mutate(Sex = recode(Sex, "M;M" = "M", "F;F" = "F")) %>% ## Collapse sex to 'M' or 'F'
  mutate(across(c(Age, PMI, RIN, pH), as.numeric)) %>% ## Convert numeric variables to
  ↪  numeric
  mutate(across(
    c(Diagnosis, BrainID, BrainRegion, Sex, Preservation, Batch),
    as.factor
  )) ## Convert categorical variables to factor

# Replace missing pH values with mean(pH)
pH.avg <- mean(na.omit(samples$pH))
samples <- samples %>%
  mutate(pH = ifelse(is.nan(pH), yes = pH.avg, no = pH))

head(samples)
```

```
##            Diagnosis   PMI  Age RIN Preservation Batch Sex    pH BrainID
## GSM1090267         A  8.80  88 6.6          LNV     1   M 6.170       1
## GSM1090268         A 12.60  90 6.3          LNV     1   F 6.164       2
## GSM1090269         N 25.80  64 6.1          LNV     1   M 6.831       3
## GSM1090270         N  7.05  95 7.2          LNV     1   F 6.454       4
## GSM1090271         A  9.25  90 6.7          LNV     1   F 6.057       5
## GSM1090272         A  6.86  77 7.2          LNV     1   F 6.793       6
##            BrainRegion geo_accession
## GSM1090267          CB    GSM1090267
## GSM1090268          CB    GSM1090268
## GSM1090269          CB    GSM1090269
## GSM1090270          CB    GSM1090270
## GSM1090271          CB    GSM1090271
## GSM1090272          CB    GSM1090272
```

Reading in pathology data

```
# Reading in pathology trait data from Yasser Iturria Medina (used in PMID 34021244)
pathology <- read_xlsx("HBTRC_Covariates.xlsx", sheet = "transpose")

# Selecting matching variables and Braak, renaming and recoding to match Sample names and
↪  levels
pathology <- pathology %>%
  dplyr::select(
    c(
      Diagnosis = 'alz_status',
      Sex = 'gender',
      PMI = 'post_mortem_interval',
      Age = 'age_death',
      RIN = 'rin',
      Preservation = 'preservation_method',
      APOEgeno = '% APOE',
      Braak
    )
  ) %>%
  ## Recode Sex to match sample factor levels
  mutate(Sex = recode(Sex, "-1" = 'F', "1" = "M")) %>%
  ## Recode Preservation to match sample factor levels
  mutate(Preservation = recode(Preservation, "-1" = 'LNV', "1" = "Dry-Ice")) %>%
  ## Recode Diagnosis to match sample factor levels
  mutate(Diagnosis = recode(Diagnosis, "0" = 'N', "1" = "A")) %>%
  ## Convert numeric variables to numeric
  mutate(across(c(PMI, Age, RIN), as.numeric)) %>%
  ## Convert categorical variables to factor
  mutate(across(c(
    Diagnosis, Sex, Preservation, APOEgeno, Braak
  ), as.factor))

head(pathology)
```

```
## # A tibble: 6 x 8
##   Diagnosis Sex     PMI   Age   RIN Preservation APOEgeno Braak
##   <fct>     <fct> <dbl> <dbl> <dbl> <fct>        <fct>    <fct>
## 1 N         F      16      63   6.1 LNV          E3/E3    nan
## 2 N         F      17.2    60   0   LNV          E2/E4    nan
## 3 N         F       9.6    31   8.2 LNV          E3/E3    nan
## 4 N         M      26.8    63   6   LNV          nan      nan
## 5 N         F      21.2    81   7.1 LNV          E3/E3    nan
## 6 N         M     NaN      44   6.2 LNV          E3/E3    nan
```

Merging pathology data with other patient-wise covariates

```
# Subset samples by brain region
PFC <- samples %>%
  filter(BrainRegion == "PFC") %>%
  rename(
    "Batch" = "Batch.PFC",
    "pH" = "pH.PFC",
```

```r
    "RIN" = "RIN.PFC",
    "geo_accession" = "geo_accession.PFC"
  ) %>%
  dplyr::select(!BrainRegion)

CB <- samples %>%
  filter(BrainRegion == "CB") %>%
  rename(
    "Batch" = "Batch.CB",
    "pH" = "pH.CB",
    "RIN" = "RIN.CB",
    "geo_accession" = "geo_accession.CB"
  ) %>%
  dplyr::select(!BrainRegion)

VC <- samples %>%
  filter(BrainRegion == "VC") %>%
  rename(
    "Batch" = "Batch.VC",
    "pH" = "pH.VC",
    "RIN" = "RIN.VC",
    "geo_accession" = "geo_accession.VC"
  ) %>%
  dplyr::select(!BrainRegion)

# Merge samples by matching variables
patients <-
  merge(PFC,
        CB,
        by = c("BrainID", "Age", "Sex", "PMI", "Diagnosis", "Preservation"))
patients <-
  merge(patients,
        VC,
        by = c("BrainID", "Age", "Sex", "PMI", "Diagnosis", "Preservation"))

# Reorder columns alphabetically
patients <- patients[, order(colnames(patients))]

head(patients)
```

```
##   Age Batch.CB Batch.PFC Batch.VC BrainID Diagnosis geo_accession.CB
## 1  88        1         1        1       1         A       GSM1090267
## 2  89        1         1        1      10         A       GSM1090276
## 3  52        1         1        1     100         N       GSM1090366
## 4  73        1         1        1     101         A       GSM1090367
## 5  73        2         2        2     102         N       GSM1090368
## 6  74        1         1        1     103         N       GSM1090369
##   geo_accession.PFC geo_accession.VC pH.CB pH.PFC pH.VC   PMI Preservation
## 1        GSM1090501       GSM1090731 6.170  6.409 6.244  8.80          LNV
## 2        GSM1090510       GSM1090740 6.145  6.353 6.283 13.80          LNV
## 3        GSM1090600       GSM1090830 6.076  6.493 6.411 13.12          LNV
## 4        GSM1090601       GSM1090831 6.560  6.302 6.381 28.58          LNV
## 5        GSM1090602       GSM1090832 6.451  6.591 6.293 20.83      Dry-Ice
```

```
## 6        GSM1090603       GSM1090833 6.811  6.956 6.883 14.33          LNV
##   RIN.CB RIN.PFC RIN.VC Sex
## 1    6.6     7.7    7.1   M
## 2    6.4     6.9    6.7   F
## 3    6.7     6.9    7.3   M
## 4    6.5     7.8    7.4   M
## 5    6.9     7.1    7.4   F
## 6    7.2     7.2    7.1   M
```

Merging sample-wise and patient-wise covariates

```
# Merge by shared patient-level variables
meta <-
  merge(
    x = patients,
    y = pathology,
    by = c("Diagnosis", "Age", "Sex", "PMI", "Preservation")
  )
meta <- meta[, order(colnames(meta))]

# Require pathology data RIN to match RIN of sample RIN from at least one brain region
meta <- meta %>%
  group_by(BrainID) %>%
  filter(RIN %in% c(RIN.CB, RIN.PFC, RIN.VC))

# Which sample IDs are missing pathology data?
setdiff(patients$BrainID, meta$BrainID)
```

```
## [1] "154"
```

```
# Add patient 154 back to meta
patient154 <- patients[which(patients$BrainID == 154), ]
pathology154 <- data.frame(matrix(ncol = 3, nrow = 1))
colnames(pathology154) <-
  setdiff(colnames(meta), colnames(patient154))
p154 <- cbind(patient154, pathology154)
meta <- rbind(meta, p154)
rm(p154, pathology154, patient154)

# Set meta rownames to geo_accession.PFC
meta <- as.data.frame(meta[order(meta$geo_accession.PFC), ])
rownames(meta) <- meta$geo_accession.PFC

head(meta)
```

```
##            Age APOEgeno Batch.CB Batch.PFC Batch.VC Braak BrainID Diagnosis
## GSM1090501  88    E3/E3        1         1        1   nan       1         A
## GSM1090502  90      nan        1         1        1   nan       2         A
## GSM1090503  64    E3/E3        1         1        1     0       3         N
## GSM1090504  95    E3/E3        1         1        1   nan       4         N
## GSM1090505  90    E3/E4        1         1        1   nan       5         A
## GSM1090506  77    E3/E4        1         1        1   nan       6         A
##            geo_accession.CB geo_accession.PFC geo_accession.VC pH.CB pH.PFC
## GSM1090501       GSM1090267        GSM1090501       GSM1090731 6.170  6.409
```

```
## GSM1090502       GSM1090268        GSM1090502       GSM1090732 6.164  5.644
## GSM1090503       GSM1090269        GSM1090503       GSM1090733 6.831  6.652
## GSM1090504       GSM1090270        GSM1090504       GSM1090734 6.454  6.378
## GSM1090505       GSM1090271        GSM1090505       GSM1090735 6.057  6.050
## GSM1090506       GSM1090272        GSM1090506       GSM1090736 6.793  6.002
##            pH.VC   PMI Preservation RIN RIN.CB RIN.PFC RIN.VC Sex
## GSM1090501 6.244  8.80          LNV 6.6    6.6     7.7    7.1   M
## GSM1090502 5.832 12.60          LNV 6.1    6.3     7.2    6.1   F
## GSM1090503 6.621 25.80          LNV 7.3    6.1     7.0    7.3   M
## GSM1090504 6.108  7.05          LNV 6.1    7.2     6.9    6.1   F
## GSM1090505 6.051  9.25          LNV 6.0    6.7     7.0    6.0   F
## GSM1090506 6.247  6.86          LNV 7.2    7.2     6.9    7.2   F
```

Summary tables of sample-wise and patient-wise covariates (S1 and S2 Tables)

```
sampleQC <- samples %>%
  filter(BrainRegion != 'VC') %>%
  group_by(BrainRegion, Diagnosis) %>%
  summarise(
    n = n(),
    pH.median = median(pH, na.rm = TRUE),
    pH.min = min(pH, na.rm = TRUE),
    pH.max = max(pH, na.rm = TRUE),
    RIN.median = median(RIN, na.rm = TRUE),
    RIN.min = min(RIN, na.rm = TRUE),
    RIN.max = max(RIN, na.rm = TRUE),
    LNV = length(which(Preservation == "LNV")),
    DryIce = length(which(Preservation == "Dry-Ice")),
    Batch1 = length(which(Batch == 1)),
    Batch2 = length(which(Batch == 2)),
    Batch3 = length(which(Batch == 3))
  )
```

```
## `summarise()` has grouped output by 'BrainRegion'. You can override using the
## `.groups` argument.
```

```
individualQC <- meta %>%
  group_by(Diagnosis) %>%
  summarise(
    n = n(),
    Age.median = median(Age, na.rm = TRUE),
    Age.min = min(Age, na.rm = TRUE),
    Age.max = max(Age, na.rm = TRUE),
    PMI.median = median(PMI, na.rm = TRUE),
    PMI.min = min(PMI, na.rm = TRUE),
    PMI.max = max(PMI, na.rm = TRUE),
    pctMale = round(100 * length(which(Sex == "M")) / length(Sex), 1),
    pctFemale = round(100 * length(which(Sex == "F")) / length(Sex), 1),
    Braak0 = length(which(Braak == "0")),
    Braak1 = length(which(Braak == "1")),
    Braak2 = length(which(Braak == "2")),
    Braak3 = length(which(Braak == "3")),
    Braak4 = length(which(Braak == "4")),
    Braak5 = length(which(Braak == "5")),
```

```r
    Braak6 = length(which(Braak == "6")),
    BraakNA = length(which(Braak == "nan" | is.na(Braak)))
  )

write_xlsx(sampleQC, path = "sampleQC.xlsx")
write_xlsx(individualQC, path = "individualQC.xlsx")

head(sampleQC)
```

```
## # A tibble: 4 x 14
## # Groups:   BrainRegion [2]
##   BrainRegion Diagno~1     n pH.me~2 pH.min pH.max RIN.m~3 RIN.min RIN.max   LNV
##   <fct>       <fct>    <int>   <dbl>  <dbl>  <dbl>   <dbl>   <dbl>   <dbl> <int>
## 1 CB          A          129    6.45   5.42   7.18     6.7     5.1     7.7   101
## 2 CB          N          101    6.61   5.83   7.69     6.7     5.3     7.4    94
## 3 PFC         A          129    6.39   5.41   7.34     7.1     5.7     8.7   101
## 4 PFC         N          101    6.62   5.54   7.42     7.3     5.9     8.2    94
## # ... with 4 more variables: DryIce <int>, Batch1 <int>, Batch2 <int>,
## #   Batch3 <int>, and abbreviated variable names 1: Diagnosis, 2: pH.median,
## #   3: RIN.median
```

```r
head(individualQC)
```

```
## # A tibble: 2 x 18
##   Diagno~1     n Age.m~2 Age.min Age.max PMI.m~3 PMI.min PMI.max pctMale pctFe~4
##   <fct>    <int>   <dbl>   <dbl>   <dbl>   <dbl>   <dbl>   <dbl>   <dbl>   <dbl>
## 1 A          129      82      55     100    12.6    1.3     34.8    48.1    51.9
## 2 N          101      61      22     106    21.9    7.05    41.7    81.2    18.8
## # ... with 8 more variables: Braak0 <int>, Braak1 <int>, Braak2 <int>,
## #   Braak3 <int>, Braak4 <int>, Braak5 <int>, Braak6 <int>, BraakNA <int>, and
## #   abbreviated variable names 1: Diagnosis, 2: Age.median, 3: PMI.median,
## #   4: pctFemale
```

Assessing expression distribution across brain regions

```r
# Set sample row names to geoaccession
samples <- as.data.frame(samples[order(samples$geo_accession), ])
rownames(samples) <- samples$geo_accession

# Replacing pData with cleaned sample level pData
pData(gse44772) <- samples

# Subsetting expression data by Brain Region
gse44772.PFC <- gse44772[, gse44772$BrainRegion == "PFC"]
gse44772.CB <- gse44772[, gse44772$BrainRegion == "CB"]

# Combine CB and PFC, then convert to long form for ggplot
eset <-
  as.data.frame(cbind(exprs(gse44772.PFC), exprs(gse44772.CB)))
eset <- eset %>%
  rownames_to_column("Probe") %>%
  pivot_longer(cols = colnames(eset),
               names_to = "GEOid",
```

```
               values_to = "Expression") %>%
  mutate(Group = as.factor(
    case_when(
      GEOid %in% meta$geo_accession.CB[meta$Diagnosis == "A"] ~ "CB.A",
      GEOid %in% meta$geo_accession.CB[meta$Diagnosis == "N"] ~ "CB.N",
      GEOid %in% meta$geo_accession.PFC[meta$Diagnosis == "A"] ~ "PFC.A",
      GEOid %in% meta$geo_accession.PFC[meta$Diagnosis == "N"] ~ "PFC.N"
    )
  )) %>%
  mutate(BrainRegion = as.factor(
    case_when(
      GEOid %in% meta$geo_accession.CB ~ "CB",
      GEOid %in% meta$geo_accession.PFC ~ "PFC"
    )
  ))
```

```
# Histograms of Expression Value Distribution by Group or Brain Region
ggplot(eset, aes(x = Expression, fill = Group)) +
  geom_histogram(bins = 50) +
  theme_classic() +
  facet_wrap(vars(Group)) +
  xlim(-1, 1) +
  geom_vline(xintercept = rep(c(
    2 * sd(eset$Expression, na.rm = TRUE),
    -2 * sd(eset$Expression, na.rm = TRUE)
  ), 2), linetype = "dashed")
```

```
## Warning: Removed 12330 rows containing non-finite values (`stat_bin()`).

## Warning: Removed 8 rows containing missing values (`geom_bar()`).
```

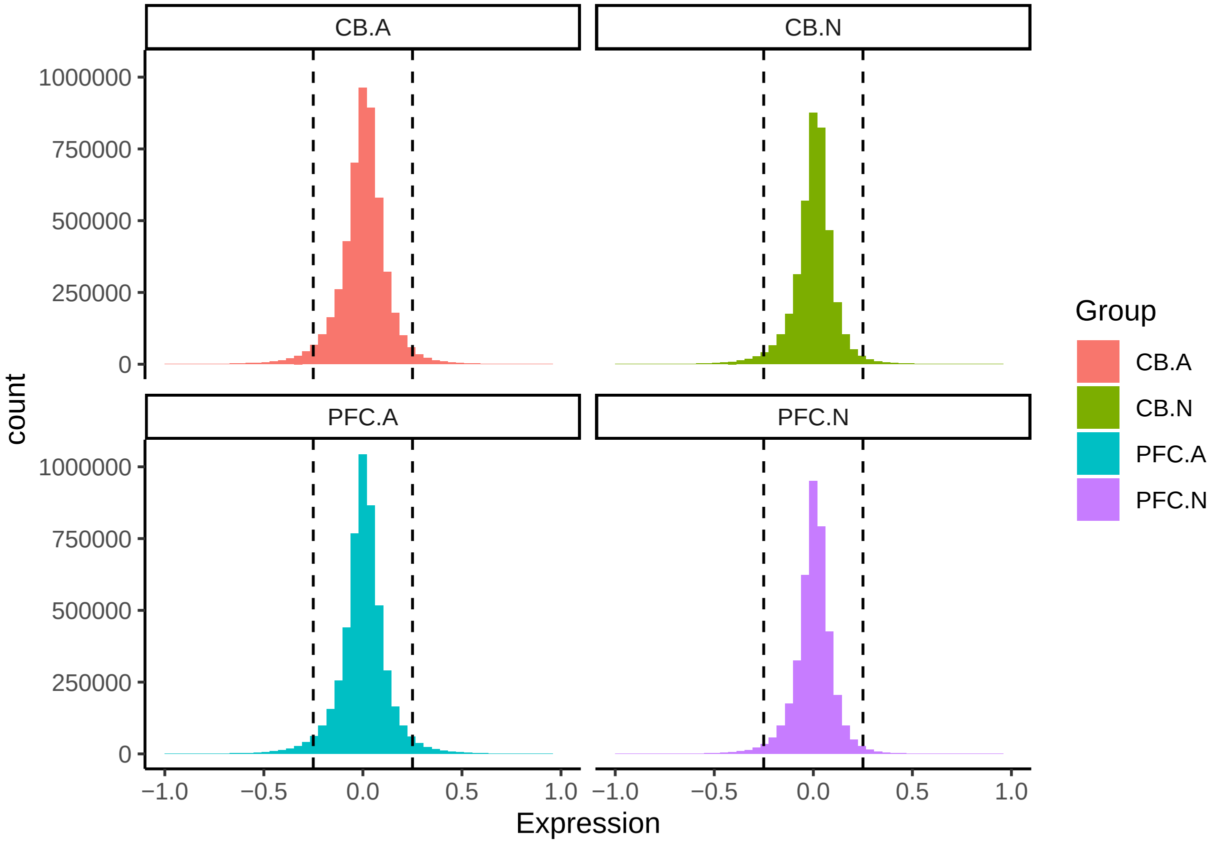


```
ggplot(eset, aes(x = Expression, fill = BrainRegion)) +
  geom_histogram(bins = 50) +
  theme_classic() +
  facet_wrap(vars(BrainRegion)) +
  xlim(-1, 1) +
  geom_vline(xintercept = rep(c(
    2 * sd(eset$Expression, na.rm = TRUE),
    -2 * sd(eset$Expression, na.rm = TRUE)
  ), 2), linetype = "dashed")
```

```
## Warning: Removed 12330 rows containing non-finite values (`stat_bin()`).
```

```
## Warning: Removed 4 rows containing missing values (`geom_bar()`).
```

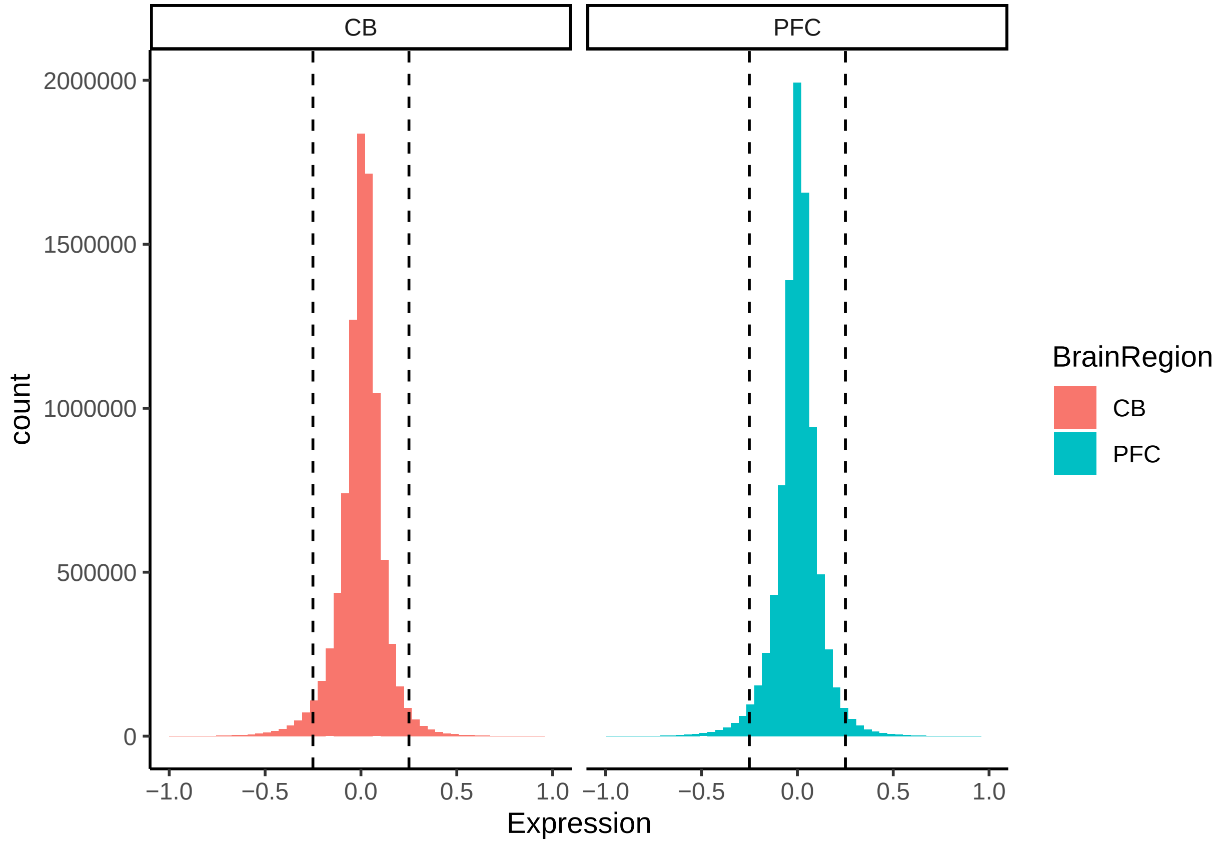


```
#rm(eset)
```

Transforming expression data from log10 to log2

```
exprs(gse44772) <-
  apply(exprs(gse44772), 1:2, function(x)
    log2(10 ^ x))
```

Building new expression objects

```
# Set sample row names to geoaccession
samples <- as.data.frame(samples[order(samples$geo_accession), ])
rownames(samples) <- samples$geo_accession

# Replacing pData with cleaned sample level pData
pData(gse44772) <- samples

# Subsetting expression data by Brain Region
gse44772.PFC <- gse44772[, gse44772$BrainRegion == "PFC"]
gse44772.CB <- gse44772[, gse44772$BrainRegion == "CB"]

# Creating RoR expression object
gse44772.RoR <-
  expSetobj(exprs(gse44772.PFC) - exprs(gse44772.CB), meta)
```

Covariate correction by linear modeling

Limma without contrast for PVCA

```r
# Limma Function with PVCA
regional_factors <-
  c('Age', 'Sex', 'Batch', 'Preservation', 'RIN', 'pH', 'PMI')
RoR_factors <-
  c(
    'Age',
    'Sex',
    'Batch.PFC',
    'Preservation',
    'PMI',
    'RIN.PFC',
    'pH.PFC',
    'RIN.CB',
    'pH.CB'
  )

limma.pvca <- function(data, batch_factors) {
  # Extract expression data before limma
  unfit <- exprs(data)

  # Calculate residuals using limma
  formula <- paste(c("~0", batch_factors), collapse = " + ")
  design <- model.matrix(as.formula(formula), data = pData(data))
  fit <- lmFit(object = data, design = design)
  fit <- eBayes(fit)
  fit <- residuals(fit, data)

  # Find and replace NAs with rowMeans
  na.id <- which(is.na(unfit), arr.ind = TRUE)
  unfit.means <- rowMeans(unfit, na.rm = TRUE)
  fit.means <- rowMeans(fit, na.rm = TRUE)

  for (i in 1:(length(rownames(na.id)))) {
    unfit[na.id[i, 1], na.id[i, 2]] <- unfit.means[na.id[i, 1]]
  }
  for (i in 1:(length(rownames(na.id)))) {
    fit[na.id[i, 1], na.id[i, 2]] <- fit.means[na.id[i, 1]]
  }

  # Create new expression objects
  unfit <- expSetobj(unfit, pData(data))
  fit <- expSetobj(fit, pData(data))

  # Run PVCA
  unfit <-
    pvcAnaly(
      exp_datObj = unfit,
      pct_threshold = 0.6,
      batch_factors = batch_factors
    )
```

```r
  fit <-
    pvcAnaly(
      exp_datObj = fit,
      pct_threshold = 0.6,
      batch_factors = batch_factors
    )

  # Output dataframe
  result <-
    data.frame(
      unfit = unfit$dat[1, ],
      fit = fit$dat[1, ],
      row.names = fit$label
    )
  return(result)
}
```

```r
PFC.pvca <- limma.pvca(gse44772.PFC, regional_factors)
```

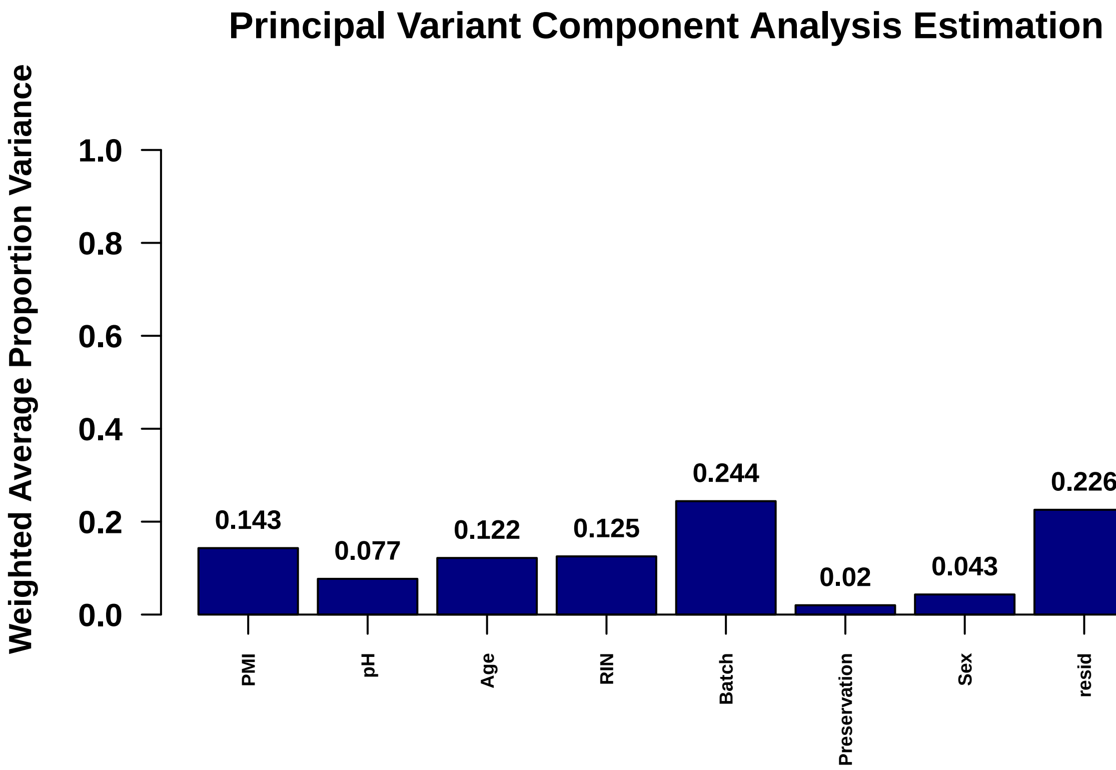

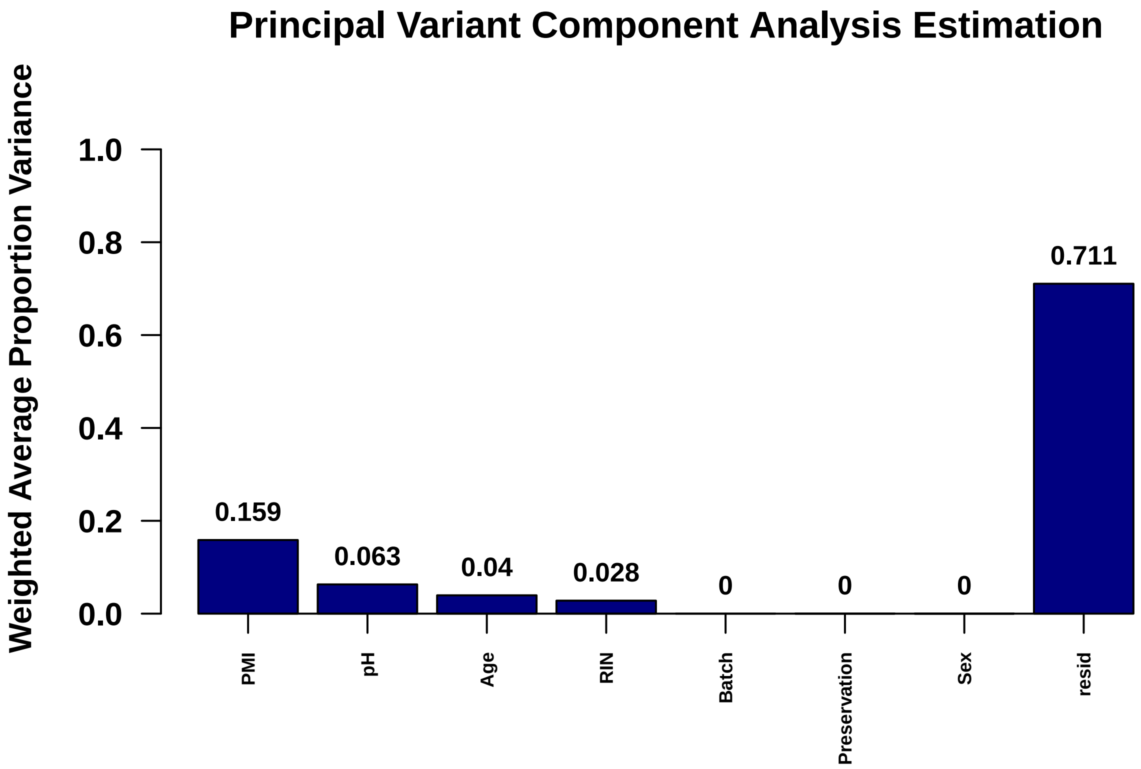


```
colnames(PFC.pvca) <- paste0(colnames(PFC.pvca), ".PFC")

PFC.pvca <- PFC.pvca %>%
  rownames_to_column('Covariate')

CB.pvca <- limma.pvca(gse44772.CB, regional_factors)
```

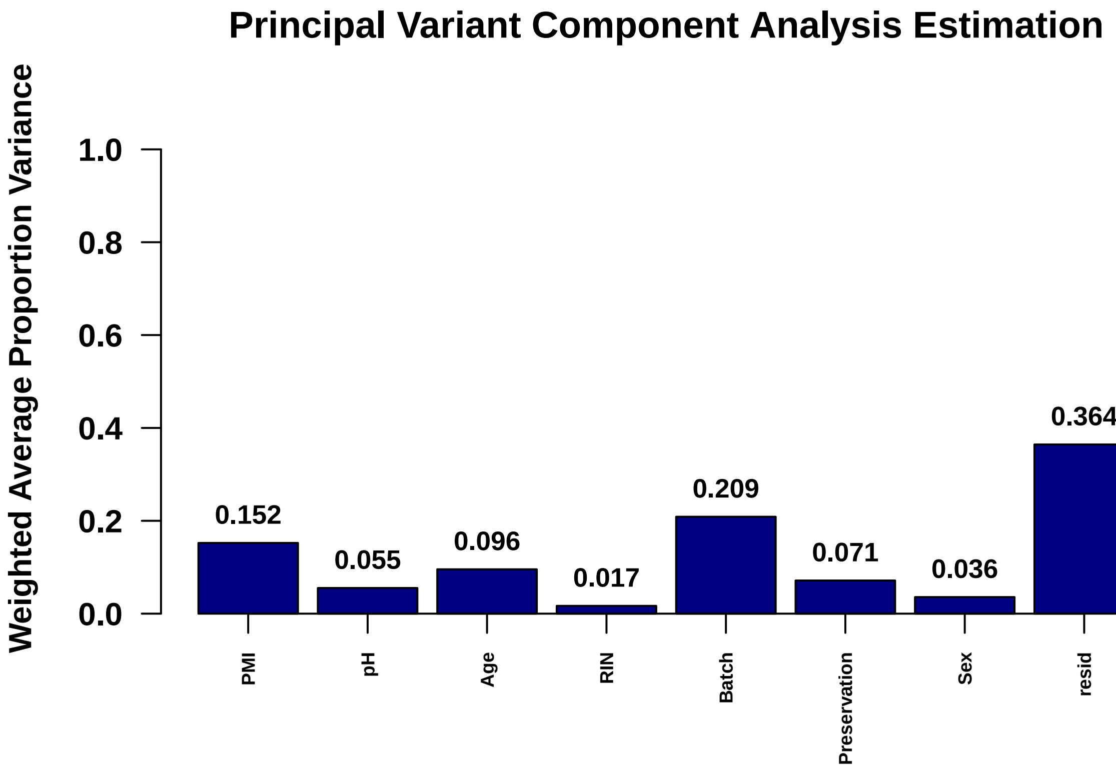
Principal Variant Component Analysis Estimation
Weighted Average Proportion Variance
1.0
0.8
0.6
0.4
0.2
0.0
0.152
0.055
0.096
0.017
0.209
0.071
0.036
0.364
PMI
pH
Age
RIN
Batch
Preservation
Sex
resid

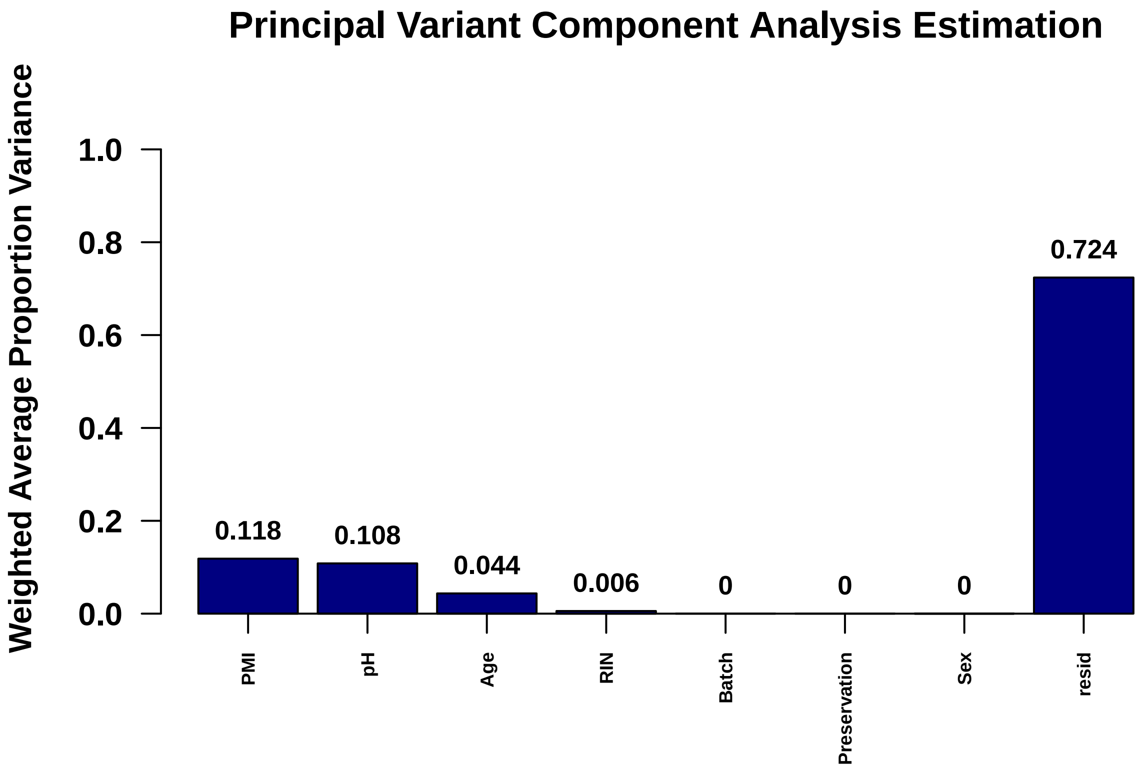


```
colnames(CB.pvca) <- paste0(colnames(CB.pvca), ".CB")

CB.pvca <- CB.pvca %>%
  rownames_to_column('Covariate')

RoR.pvca <- limma.pvca(gse44772.RoR, RoR_factors)
```

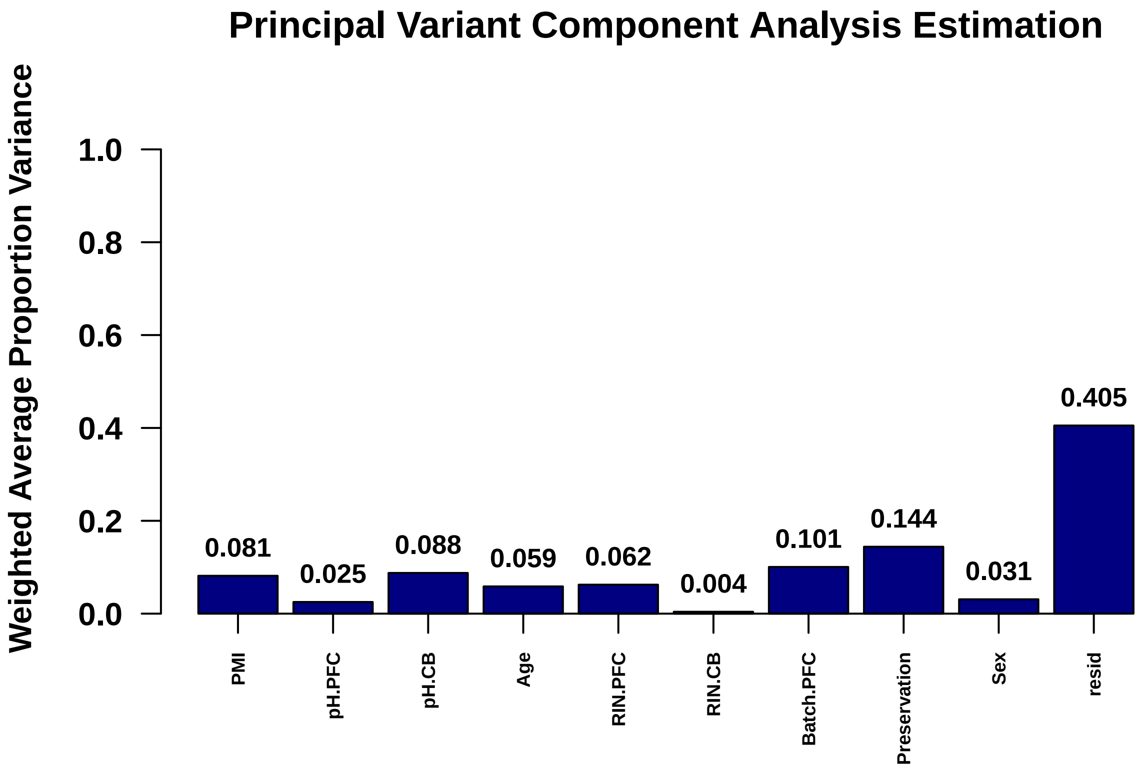

Principal Variant Component Analysis Estimation
Weighted Average Proportion Variance
1.0
0.8
0.6
0.4
0.2
0.0
0.081
0.025
0.088
0.059
0.062
0.004
0.101
0.144
0.031
0.405
PMI
pH.PFC
pH.CB
Age
RIN.PFC
RIN.CB
Batch.PFC
Preservation
Sex
resid

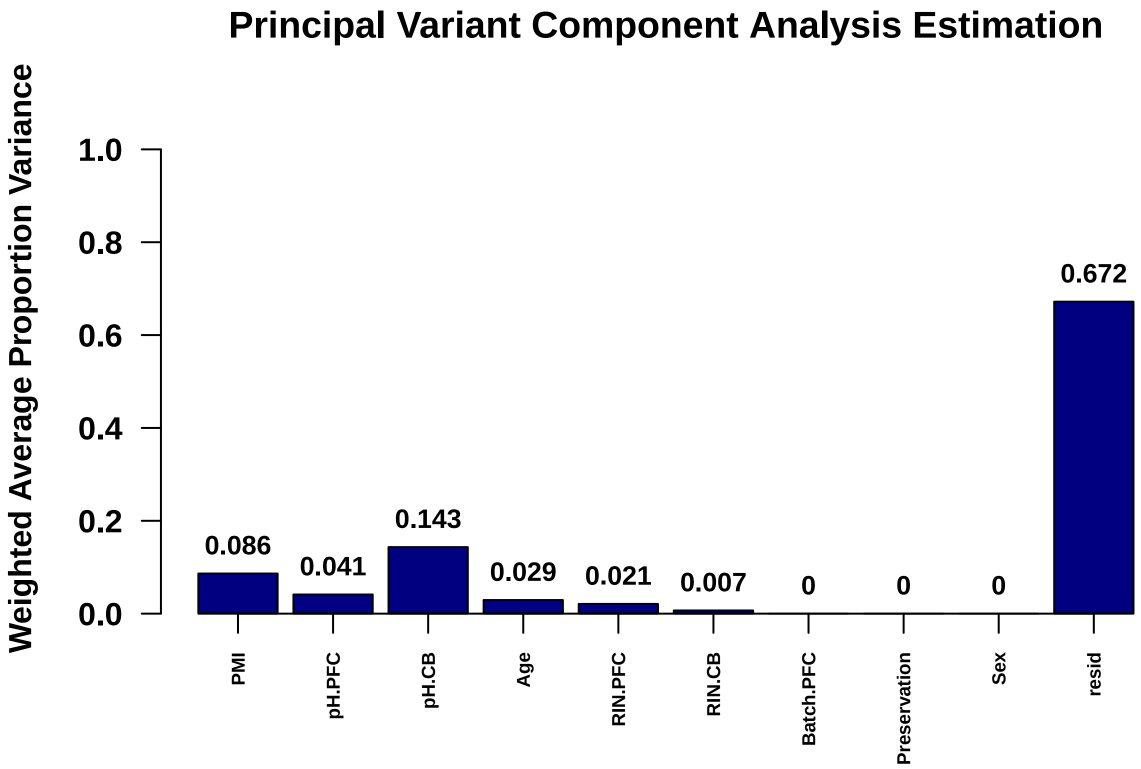


```
colnames(RoR.pvca) <- paste0(colnames(RoR.pvca), ".RoR")

# Pooling variance due to region-specific pH and RIN
RoR.pvca <- as.data.frame(t(RoR.pvca)) %>%
  mutate(pH = pH.PFC + pH.CB,
         RIN = RIN.PFC + RIN.CB,
         Batch = Batch.PFC) %>%
  dplyr::select(c(regional_factors, resid)) %>%
  t()
RoR.pvca <- as.data.frame(RoR.pvca) %>%
  rownames_to_column('Covariate')

# Combining results into single dataframe
pvca.results <- merge(PFC.pvca, CB.pvca, by = 'Covariate')
pvca.results <- merge(pvca.results, RoR.pvca, by = 'Covariate')

pvca.results <- pvca.results %>%
  gather(PVCA, Variance, 2:7) %>%
  separate(col = PVCA,
           into = c('Fit', 'BrainRegion'),
           sep = "[.]")
```

Single plot in ggplot to summarize PVCA results (Fig 2)

```r
# Reorder variables for plotting below
pvca.results$Fit <-
  factor(pvca.results$Fit, levels = c('unfit', 'fit'))
pvca.results$Covariate <-
  factor(
    pvca.results$Covariate,
    levels = c(
      "Age",
      "Batch",
      "pH",
      "PMI",
      "Preservation",
      "RIN",
      "Sex",
      "resid"
    )
  )
pvca.results$BrainRegion <-
  factor(pvca.results$BrainRegion, levels = c("PFC", "CB", "RoR"))

ggplot(data = pvca.results, aes(x = Covariate, y = Variance, fill = BrainRegion)) +
  theme_classic() +
  geom_bar(stat = 'identity', position = 'dodge') +
  ggtitle('PVCA Analysis of Covariate Contribution to Expression Values') +
  facet_grid(cols = vars(Fit)) +
  theme(
    plot.title = element_text(hjust = 0.5),
    axis.text.x = element_text(
      angle = 45,
      vjust = 1,
      hjust = 1
    )
  )
```

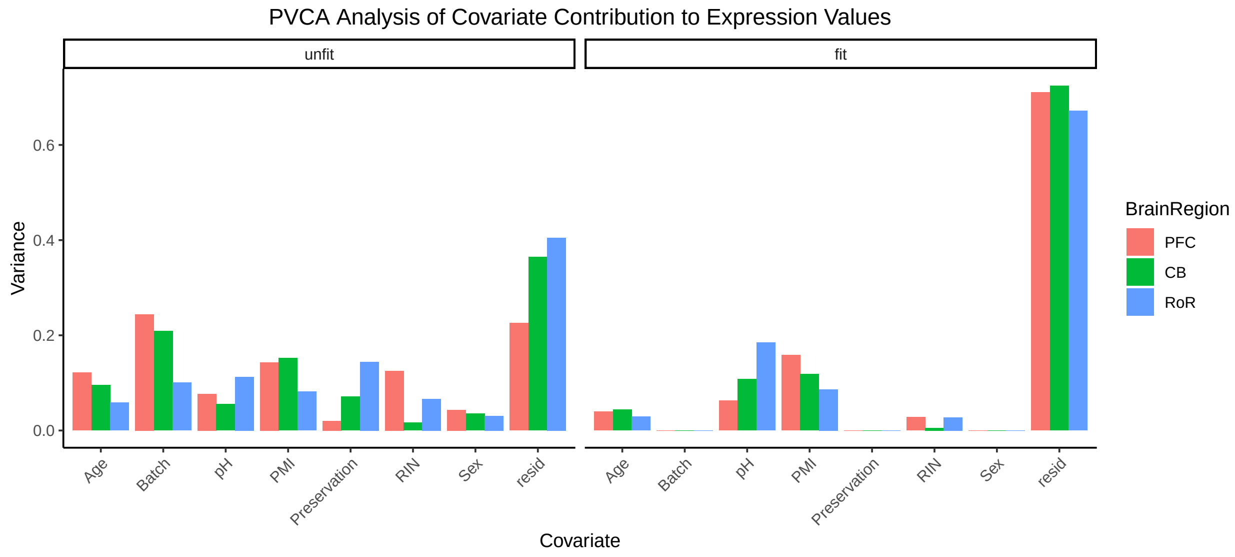

Differential expression

Limma with contrast matrix for TopTable

```
# Limma Function with TopTable
limma.TT <- function(data, batch_factors) {
  # Design and contrast matrices
  formula <-
    paste(c("~0", "Diagnosis", batch_factors), collapse = " + ")
  design <- model.matrix(as.formula(formula), data = pData(data))
  contrast <-  makeContrasts(DiagnosisA - DiagnosisN, levels = design)

  # Calculating linear model
  fit <- lmFit(object = data, design = design)
  fit <- contrasts.fit(fit, contrast)
  fit <- eBayes(fit)

  # Output TopTable
  result <- topTable(fit, number = 39280, confint = TRUE)
  result$rank <- rank(-abs(result$logFC))
  result$ID <- as.factor(rownames(result))
  return(result)
}
```

```
PFC.deg <- limma.TT(gse44772.PFC, regional_factors)
CB.deg <- limma.TT(gse44772.CB, regional_factors)
RoR.deg <- limma.TT(gse44772.RoR, RoR_factors)
```

```
# Read in identifiers key
ORF_map <- read_xlsx("identifiers.xlsx", sheet = "ORF_map")
ID_map <- read_xlsx("identifiers.xlsx", sheet = "ID_map")
Usymbol_map <- read_xlsx("identifiers.xlsx", sheet = "Usymbol_map")
GB_map <- read_xlsx("identifiers.xlsx", sheet = "GB_map")

# Find best identifier to update gene names
for (i in 1:39280) {
  if (PFC.deg$Gene.ID[i] != "") {
    PFC.deg$BestID[i] <-
      ID_map$symbol[which(ID_map$query == PFC.deg$Gene.ID[i])][1]
  }
  else if (PFC.deg$Platform_ORF[i] != "") {
    PFC.deg$BestID[i] <-
      ORF_map$symbol[which(ORF_map$query == PFC.deg$Platform_ORF[i])][1]
  }
  else if (PFC.deg$UniGene.symbol[i] != "") {
    PFC.deg$BestID[i] <-
      Usymbol_map$symbol[which(Usymbol_map$query == PFC.deg$UniGene.symbol[i])][1]
  }
  else if (PFC.deg$GenBank.Accession[i] != "") {
    PFC.deg$BestID[i] <-
      GB_map$symbol[which(GB_map$query == PFC.deg$GenBank.Accession[i])][1]
  }
  else if (PFC.deg$Gene.symbol[i] != "") {
    PFC.deg$BestID[i] <- PFC.deg$Gene.symbol[i]
```

```r
  }
  else {
    PFC.deg$BestID[i] <- NA
  }
}

rm(ID_map, ORF_map, Usymbol_map, GB_map)
```

```r
# Remove excess columns
cols <-
  c(
    "ID",
    "BestID",
    "logFC",
    "CI.L",
    "CI.R",
    "AveExpr",
    "t",
    "P.Value",
    "adj.P.Val",
    "B",
    "rank"
  )

PFC.deg <- PFC.deg %>%
  dplyr::select(which(colnames(PFC.deg) %in% cols)) %>%
  setNames(paste0(names(.), ".PFC"))

CB.deg <- CB.deg %>%
  dplyr::select(which(colnames(CB.deg) %in% cols)) %>%
  setNames(paste0(names(.), ".CB"))

RoR.deg <- RoR.deg %>%
  dplyr::select(which(colnames(RoR.deg) %in% cols)) %>%
  setNames(paste0(names(.), ".RoR"))

# Merge TopTables and rearrange columns
total.deg <-
  merge(RoR.deg,
        PFC.deg,
        by.x = "ID.RoR",
        by.y = "ID.PFC",
        all.y = FALSE)
total.deg <-
  merge(total.deg,
        CB.deg,
        by.x = "ID.RoR",
        by.y = "ID.CB",
        all.y = FALSE)
total.deg <- total.deg %>%
  relocate(c(ID = ID.RoR, BestID = BestID.PFC))

# Calculate prioritization score and sort
```

```r
total.deg <- total.deg %>%
  mutate(Prioritization = rank.PFC / rank.RoR) %>%
  arrange(Prioritization)

write_xlsx(total.deg, path = paste0(format(Sys.Date(), format = "%y%m%d_"), "toptable",
↪  ".xlsx"))

total.deg$BestID <- as.character(total.deg$BestID)
head(total.deg)
```

```
##            ID  BestID  logFC.RoR    CI.L.RoR  CI.R.RoR AveExpr.RoR      t.RoR
## 1 10025903807    MAFF -0.0337005 -0.28692293 0.2195219   0.2095410 -0.2622784
## 2 10023809033  S100A9  0.2110714 -0.07609639 0.4982391  -0.2923145  1.4485119
## 3 10023828117  S100A8  0.3451203  0.04196200 0.6482786  -0.5498267  2.2435176
## 4 10025910394 SLCO4A1  0.2519970  0.09903672 0.4049572  -0.1675272  3.2467224
## 5 10025913228    RBM3  0.0513022 -0.11121929 0.2138237   0.1034532  0.6220906
## 6 10033668510 SLCO4A1  0.2232438  0.07352168 0.3729658  -0.1542029  2.9384744
##   P.Value.RoR adj.P.Val.RoR     B.RoR rank.RoR logFC.PFC   CI.L.PFC   CI.R.PFC
## 1 0.793350644   0.889603577 -6.428993    30005  1.296777  0.9836928  1.6098620
## 2 0.148888579   0.315580799 -5.438994     6791  1.467985  0.9966933  1.9392757
## 3 0.025853245   0.089370364 -4.023293     2201  1.599760  1.0677973  2.1317231
## 4 0.001348570   0.009199589 -1.416771     4837  1.369909  1.0801858  1.6596312
## 5 0.534522343   0.711971435 -6.273150    25696 -1.117975 -1.4205593 -0.8153897
## 6 0.003647474   0.020091543 -2.311791     6144  1.346294  1.0597799  1.6328078
##   AveExpr.PFC     t.PFC  P.Value.PFC adj.P.Val.PFC     B.PFC rank.PFC  logFC.CB
## 1  -0.4387335  8.162306 2.400085e-14  2.973986e-12 22.085137       14  1.472168
## 2  -1.0298286  6.138204 3.770683e-09  5.685698e-08 10.501658        5  1.390586
## 3  -1.3369604  5.926288 1.164502e-08  1.499726e-07  9.416353        3  1.385937
## 4  -0.4268461  9.317904 1.183489e-17  9.684888e-15 29.490555        7  1.220278
## 5  -0.5018853 -7.281049 5.610534e-12  2.403291e-10 16.792996       41 -1.282303
## 6  -0.4191853  9.259837 1.753915e-17  1.230246e-14 29.107679       10  1.224730
##      CI.L.CB    CI.R.CB AveExpr.CB      t.CB   P.Value.CB adj.P.Val.CB
## 1  1.0742354  1.8701002 -0.6482745  7.290557 5.318935e-12 1.207675e-09
## 2  0.9112558  1.8699162 -0.7375142  5.717099 3.457164e-08 1.018735e-06
## 3  0.8593854  1.9124891 -0.7871337  5.186986 4.795189e-07 9.407062e-06
## 4  0.9795842  1.4609723 -0.2593189  9.990936 1.165584e-19 2.777449e-15
## 5 -1.5976854 -0.9669198 -0.6053385 -8.012447 6.243668e-14 5.137994e-11
## 6  0.9824845  1.4669748 -0.2649824  9.963176 1.414180e-19 2.777449e-15
##        B.CB rank.CB Prioritization
## 1 16.858498       1   0.0004665889
## 2  8.439451       2   0.0007362686
## 3  5.936422       3   0.0013630168
## 4 33.878204       9   0.0014471780
## 5 21.140747       6   0.0015955791
## 6 33.691404       8   0.0016276042
```

Prioritization Scheme (Fig 3A, Fig 3B)

- 'set1' genes are defined as the top 500 named probes by prioritization score, filtered for significant adjusted p-values < 0.05
- 'set2' genes are defined as the bottom 500 named probes by prioritization score, filtered for non-significant adjusted p-values > 0.05
- 'nonprioritized' are the remainder

```r
# Subset probes with gene names
named.deg <- total.deg[which(!is.na(total.deg$BestID)), ]
named.deg$Transcript <- c(1:length(rownames(named.deg)))

# Categorize genes as set1, set2, or nonprioritized
named.deg <- named.deg %>%
  mutate(Prioritized = as.factor(
    case_when(
      Transcript > nrow(named.deg) - 501 & adj.P.Val.RoR < 0.05 ~ "set1",
      Transcript < 501 &
        adj.P.Val.RoR > 0.05 ~ "set2",
      TRUE ~ "nonprioritized"
    )
  ))

set1 <- named.deg$BestID[which(named.deg$Prioritized == "set1")]
set2 <- named.deg$BestID[which(named.deg$Prioritized == "set2")]

write_xlsx(named.deg, path = paste0(
  format(Sys.Date(), format = "%y%m%d_"),
  "named_toptable",
  ".xlsx"
))
head(named.deg)
```

```
##            ID  BestID  logFC.RoR    CI.L.RoR  CI.R.RoR AveExpr.RoR      t.RoR
## 1 10025903807    MAFF -0.0337005 -0.28692293 0.2195219   0.2095410 -0.2622784
## 2 10023809033  S100A9  0.2110714 -0.07609639 0.4982391  -0.2923145  1.4485119
## 3 10023828117  S100A8  0.3451203  0.04196200 0.6482786  -0.5498267  2.2435176
## 4 10025910394 SLCO4A1  0.2519970  0.09903672 0.4049572  -0.1675272  3.2467224
## 5 10025913228    RBM3  0.0513022 -0.11121929 0.2138237   0.1034532  0.6220906
## 6 10033668510 SLCO4A1  0.2232438  0.07352168 0.3729658  -0.1542029  2.9384744
##   P.Value.RoR adj.P.Val.RoR     B.RoR rank.RoR logFC.PFC   CI.L.PFC   CI.R.PFC
## 1 0.793350644   0.889603577 -6.428993    30005  1.296777  0.9836928  1.6098620
## 2 0.148888579   0.315580799 -5.438994     6791  1.467985  0.9966933  1.9392757
## 3 0.025853245   0.089370364 -4.023293     2201  1.599760  1.0677973  2.1317231
## 4 0.001348570   0.009199589 -1.416771     4837  1.369909  1.0801858  1.6596312
## 5 0.534522343   0.711971435 -6.273150    25696 -1.117975 -1.4205593 -0.8153897
## 6 0.003647474   0.020091543 -2.311791     6144  1.346294  1.0597799  1.6328078
##   AveExpr.PFC     t.PFC  P.Value.PFC adj.P.Val.PFC     B.PFC rank.PFC  logFC.CB
## 1  -0.4387335  8.162306 2.400085e-14  2.973986e-12 22.085137       14  1.472168
## 2  -1.0298286  6.138204 3.770683e-09  5.685698e-08 10.501658        5  1.390586
## 3  -1.3369604  5.926288 1.164502e-08  1.499726e-07  9.416353        3  1.385937
## 4  -0.4268461  9.317904 1.183489e-17  9.684888e-15 29.490555        7  1.220278
## 5  -0.5018853 -7.281049 5.610534e-12  2.403291e-10 16.792996       41 -1.282303
## 6  -0.4191853  9.259837 1.753915e-17  1.230246e-14 29.107679       10  1.224730
##      CI.L.CB    CI.R.CB AveExpr.CB      t.CB   P.Value.CB adj.P.Val.CB
## 1  1.0742354  1.8701002 -0.6482745  7.290557 5.318935e-12 1.207675e-09
## 2  0.9112558  1.8699162 -0.7375142  5.717099 3.457164e-08 1.018735e-06
## 3  0.8593854  1.9124891 -0.7871337  5.186986 4.795189e-07 9.407062e-06
## 4  0.9795842  1.4609723 -0.2593189  9.990936 1.165584e-19 2.777449e-15
## 5 -1.5976854 -0.9669198 -0.6053385 -8.012447 6.243668e-14 5.137994e-11
## 6  0.9824845  1.4669748 -0.2649824  9.963176 1.414180e-19 2.777449e-15
```

```
##        B.CB rank.CB Prioritization Transcript    Prioritized
## 1 16.858498       1   0.0004665889          1           set2
## 2  8.439451       2   0.0007362686          2           set2
## 3  5.936422       3   0.0013630168          3           set2
## 4 33.878204       9   0.0014471780          4 nonprioritized
## 5 21.140747       6   0.0015955791          5           set2
## 6 33.691404       8   0.0016276042          6 nonprioritized
```

```
ggplot(data = named.deg, aes(
  x = Prioritization,
  y = rank(Prioritization),
  color = Prioritized
)) +
  geom_jitter(width = 0.14, height = 700) +
  theme_classic() +
  scale_x_log10() +
  labs(x = "log(Desirability Score)", y = "Desirability Rank", title = 'Prioritization
  ↪  Scheme') +
  theme(plot.title = element_text(hjust = 0.5),
        legend.position = c(0.85, 0.15)) +
  scale_color_manual(values = c(
    'set1' = rgb(154, 3, 0, maxColorValue = 255),
    'set2' = rgb(0, 0, 142, maxColorValue = 255),
    'nonprioritized' = 'black'
  ))
```

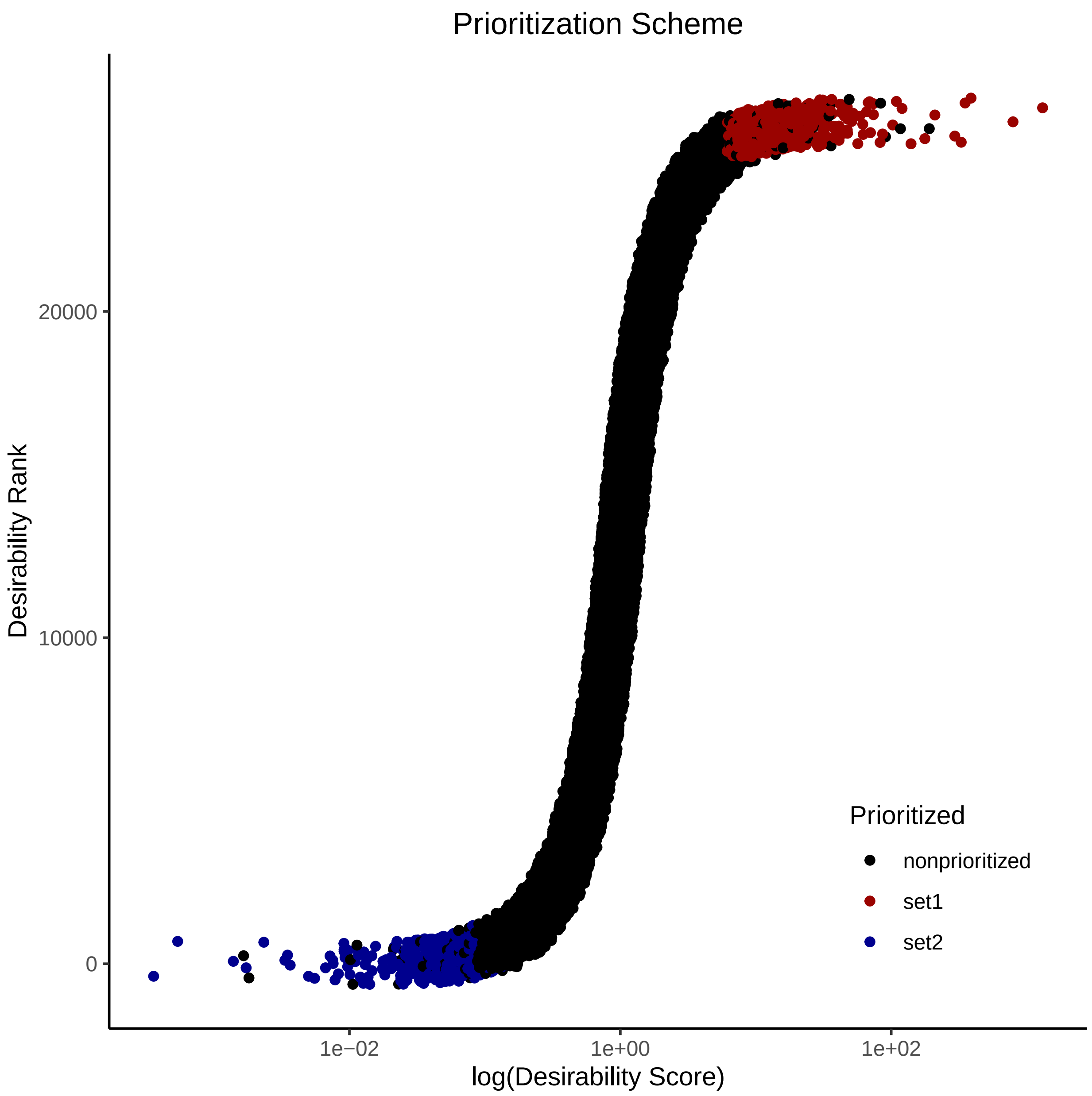

Overlap with previous publications

Instructions for GeneOverlap accessed here: https://www.bioconductor.org/packages/release/bioc/vignettes/GeneOverlap/inst/doc/GeneOverlap.pdf https://www.bioconductor.org/packages/release/bioc/manuals/GeneOverlap/man/GeneOverlap.pdf

```
AyoubLists <- split(named.deg$BestID, named.deg$Prioritized)
AyoubLists <- AyoubLists[c('set1', 'set2')]
```

Overlap with Zhang et. al Braak-stage correlated genes (Fig 3C)

Gene lists from Zhang 2013 retrieved from "Braak stage correlation" tab in supplemental data accessible here: http://dx.doi.org/10.1016/j.cell.2013.03.030.

```
# Read in Braak-stage correlated genes from Zhang et al. 2013 Supplemental Data
ZhangBraak <-
  read_xlsx('Zhang2013_SI.xlsx', sheet = 'Braak stage correlation')

# Gene overlap matrix
ZhangBraak <- split(ZhangBraak$Gene, ZhangBraak$`Brain Region`)
ZhangBraak$Both <- intersect(ZhangBraak$CB, ZhangBraak$PFC)
ZhangBraak$Union <- union(ZhangBraak$CB, ZhangBraak$PFC)

ZhangBraak.gom <-
  newGOM(AyoubLists, ZhangBraak[c("CB", "PFC", "Both", "Union")], spec = 'hg19.gene')

# Heatmap
drawHeatmap(
  ZhangBraak.gom,
  what = 'Jaccard',
  adj.p = TRUE,
  grid.col = "Reds",
  note.col = 'grey40'
)
```

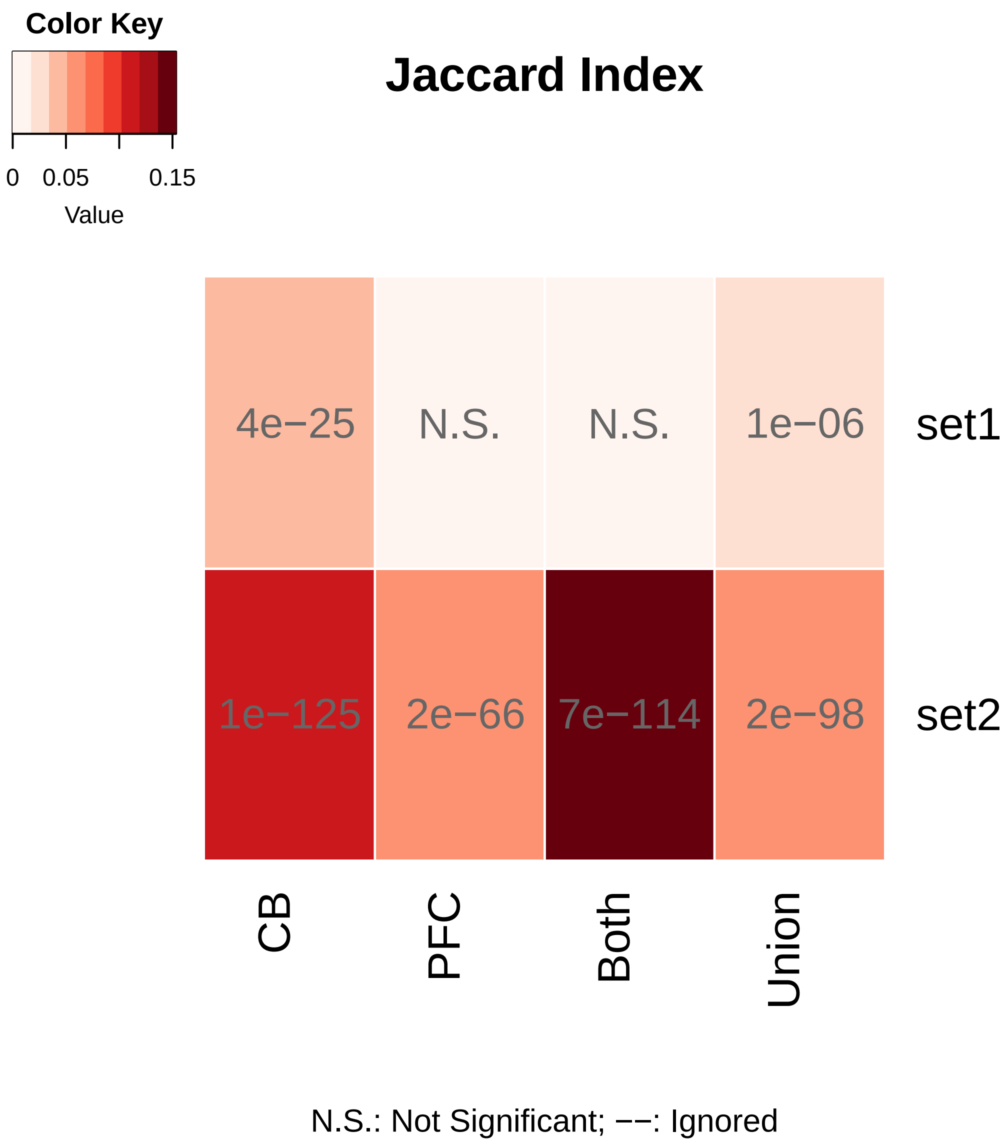

Overlap with McKenzie et al. cell-type specific marker genes (Fig 4)

Cell-type specific markers retrieved from Supplemental File 1 at: https://www.nature.com/articles/s41598-018-27293-5#Sec21.

```
# Read in marker genes from McKenzie et al. 2018 Supplemental Data
HumanSpecificity <-
  read_xlsx(path = "McKenzie2018supp.xlsx",
            sheet = "top_human_specificity",
            range = "A3:E5003")

# Split by cell-type and select top 500 markers
HumanSpecificity <-
  split(HumanSpecificity$gene, HumanSpecificity$Celltype)
for (i in 1:5) {
  HumanSpecificity[[i]] <- HumanSpecificity[[i]][1:500]
}
```

```r
# Gene overlap matrix
HumanSpecificity.gom <-
  newGOM(AyoubLists, HumanSpecificity, spec = 'hg19.gene')

# Heatmap
drawHeatmap(
  HumanSpecificity.gom,
  what = 'Jaccard',
  adj.p = TRUE,
  grid.col = "Reds",
  note.col = 'grey40'
)
```

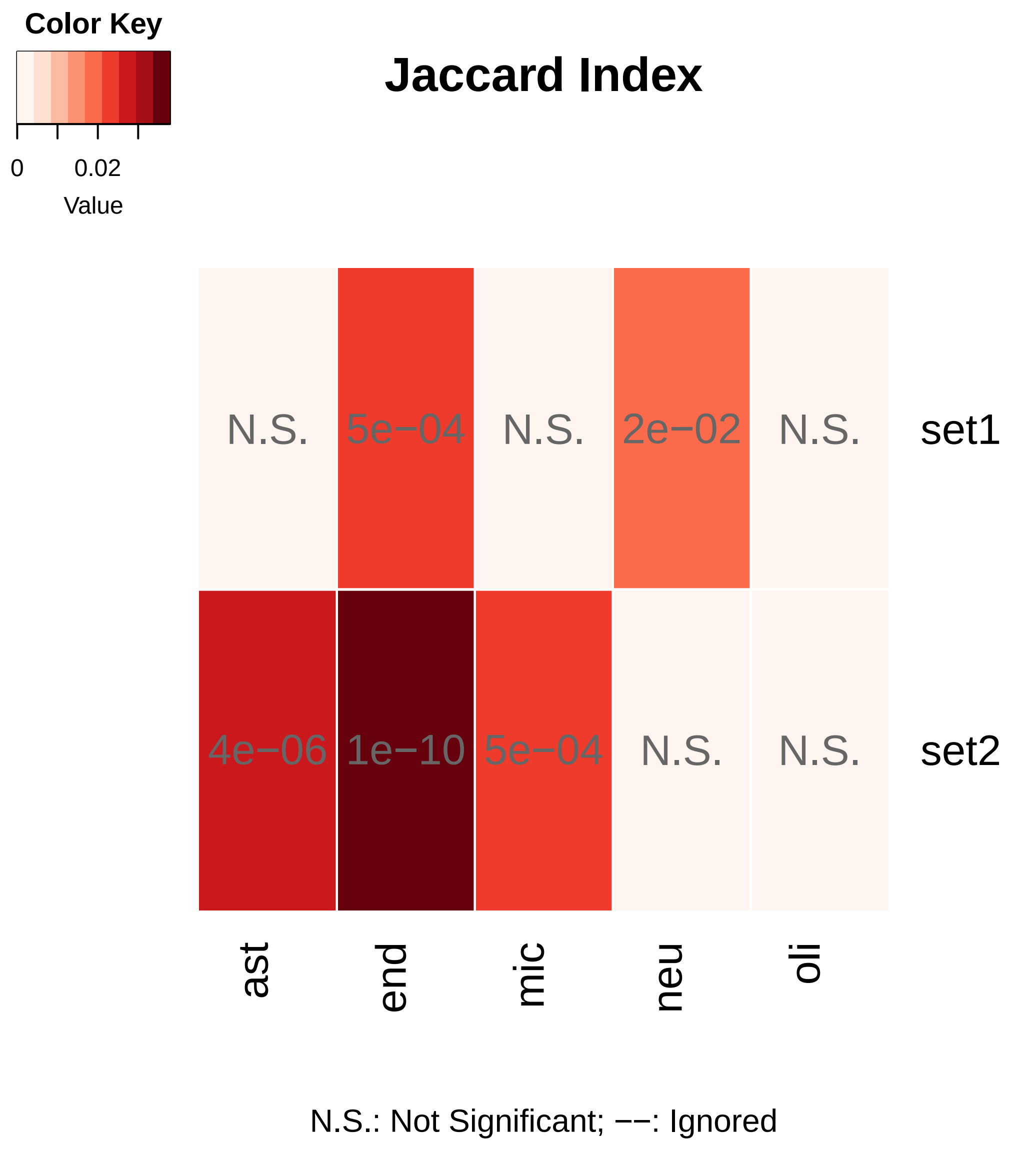

Plotting specific gene sets

Heat Shock Set 1 Genes (Fig 7A)

```
RegCellHeat.ids <-
  c(
    'ARPP21',
    'BAG2',
    'DNAJA1',
    'DNAJB4',
    'DNAJB6',
    'HSP90AA1',
    'HSP90AB1',
    'HSPA1L',
    'HSPA8',
    'HSPH1',
    'IER5',
    'MLST8',
    'PSIP1',
    'SLU7',
    'SOD1'
  )
RegCellHeat <-
  named.deg[which(named.deg$BestID %in% RegCellHeat.ids &
                    named.deg$Prioritized == 'set1'), ]

RegCellHeat.RoR <- RegCellHeat %>%
  dplyr::select(BestID, logFC = logFC.RoR) %>%
  mutate(Analysis = 'RoR')

RegCellHeat.PFC <- RegCellHeat %>%
  dplyr::select(BestID, logFC = logFC.PFC) %>%
  mutate(Analysis = 'PFC-only')

RegCellHeat <- rbind(RegCellHeat.PFC, RegCellHeat.RoR)
RegCellHeat$Analysis <- as.factor(RegCellHeat$Analysis)

RegCellHeat$BestID <-
  factor(
    RegCellHeat$BestID,
    levels = c(
      'MLST8',
      'PSIP1',
      'SLU7',
      'HSP90AB1',
      'BAG2',
      'ARPP21',
      'HSPA1L',
      'SOD1',
      'DNAJB4',
      'DNAJB6',
      'HSP90AA1',
      'IER5',
      'HSPA8',
```

```
      'DNAJA1',
      'HSPH1'
    )
  )

ggplot(data = RegCellHeat, aes(x = BestID, y = logFC, fill = Analysis)) +
  theme_classic() +
  geom_col(position = 'dodge') +
  ggtitle('Fold Change Expression of Regulation Cellular Heat Genes') +
  theme(
    plot.title = element_text(hjust = 0.5),
    axis.text.x = element_text(
      angle = 45,
      vjust = 1,
      hjust = 1
    )
  ) +
  ylim(-0.8, 0.8)
```

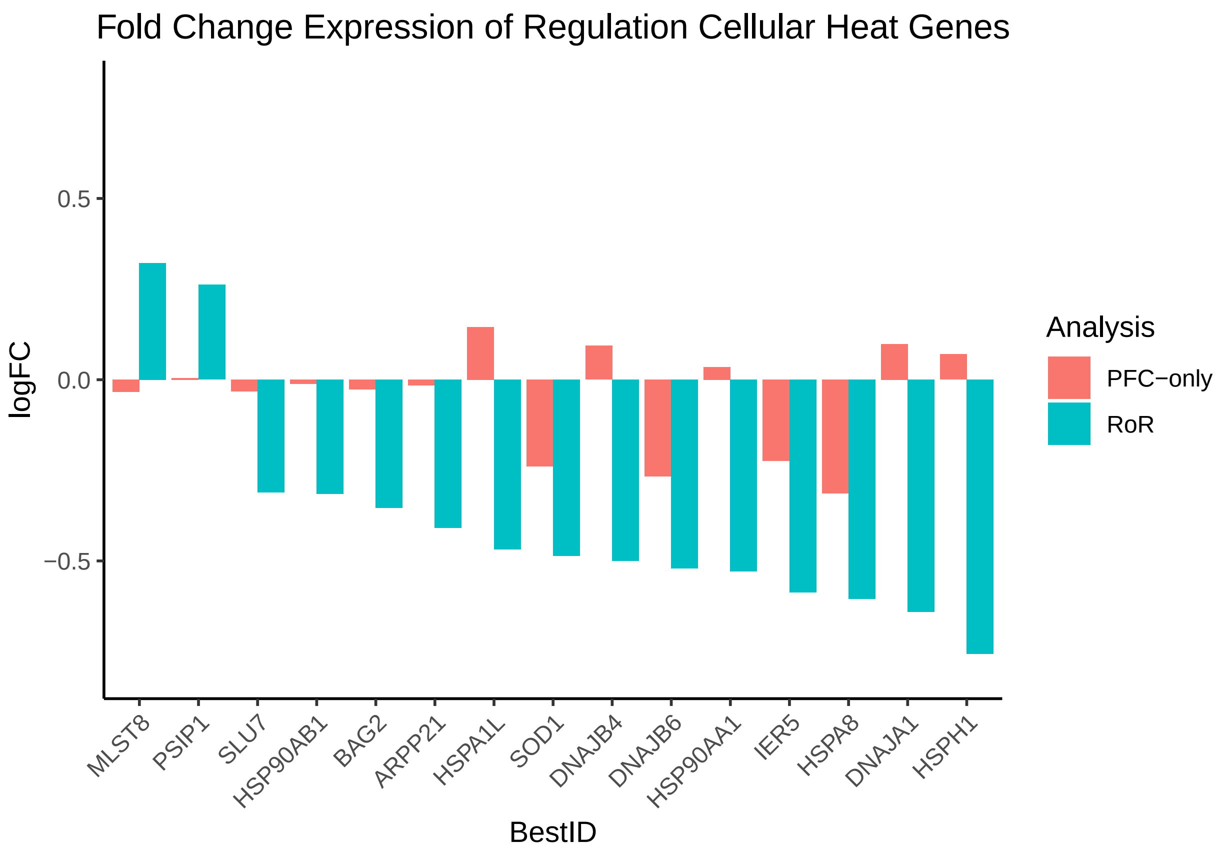

Immune Set 2 Genes (Fig 7B)

```
# Figure 6 Panel B Immune Genes
immune.ids <-
  c(
    "IL10RA",
```

```r
    "IL15RA",
    "IL18R1",
    "IL1RL1",
    "IL3RA",
    "IL4R",
    "IL6" ,
    "CX3CR1",
    "CXCL1",
    "CXCL12",
    "CXCR4"
  )
ImmuneSet2 <-
  named.deg[which(named.deg$BestID %in% immune.ids &
                    named.deg$Prioritized == 'set2'), ]

ImmuneSet2.RoR <- ImmuneSet2 %>%
  dplyr::select(BestID, logFC = logFC.RoR) %>%
  mutate(Analysis = 'RoR')

ImmuneSet2.PFC <- ImmuneSet2 %>%
  dplyr::select(BestID, logFC = logFC.PFC) %>%
  mutate(Analysis = 'PFC-only')

ImmuneSet2 <- rbind(ImmuneSet2.PFC, ImmuneSet2.RoR)
ImmuneSet2$Analysis <- as.factor(ImmuneSet2$Analysis)

ggplot(data = ImmuneSet2, aes(x = BestID, y = logFC, fill = Analysis)) +
  theme_classic() +
  geom_col(position = 'dodge') +
  ggtitle('Fold Change Expression of Regulation Cellular Heat Genes') +
  theme(
    plot.title = element_text(hjust = 0.5),
    axis.text.x = element_text(
      angle = 45,
      vjust = 1,
      hjust = 1
    )
  ) +
  ylim(-1, 1)
```

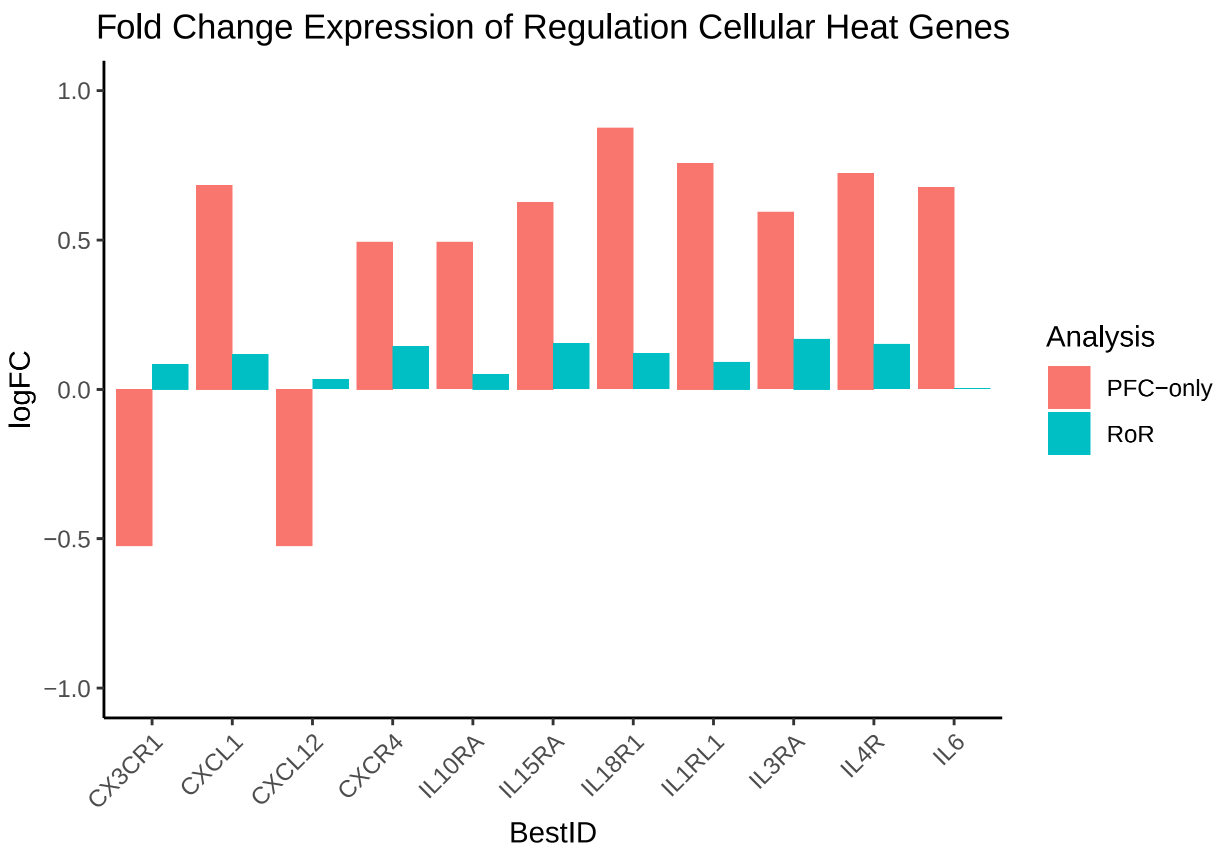

Overlap with reported AD GWAS variants (S10 Table and S11 Table, Fig 10) GWAS list from Jansen 2019 Supplementary Table 13 retrieved from https://doi.org/10.1038/s41588-018-0311-9.

GWAS list from Kunkle 2019 Supplementary Table 8 retrieved from https://doi.org/10.1038/s41588-019-0358-2.

GWAS list from Wightman 2021 Supplementary Table 10 retrieved from https://doi.org/10.1038/s41588-021-00921-z.

```
# Reading in GWAS Variants
GWASvariants <- read_xlsx('GWASvariants.xlsx', range = "A1:D1240")
Jansen2019 <- GWASvariants$Jansen2019
Kunkle2019 <- GWASvariants$Kunkle2019
Wightman2021 <- GWASvariants$Wightman2021
PooledGWAS <- GWASvariants$Pooled

# Identifying Intersecting Genes
Jansenset1 <- intersect(Jansen2019, AyoubLists$Prioritized)
Jansenset2 <- intersect(Jansen2019, AyoubLists$set2)
Kunkleset1 <- intersect(Kunkle2019, AyoubLists$Prioritized)
Kunkleset2 <- intersect(Kunkle2019, AyoubLists$set2)
Wightmanset1 <- intersect(Wightman2021, AyoubLists$Prioritized)
Wightmanset2 <- intersect(Wightman2021, AyoubLists$set2)
Pooledset1 <- intersect(PooledGWAS, AyoubLists$Prioritized)
Pooledset2 <- intersect(PooledGWAS, AyoubLists$set2)

# Subsetting Expression Table for Intersecting Genes
set1GWAS <- named.deg[which(named.deg$BestID %in% Pooledset1),]
```

```r
set1GWAS <-
  set1GWAS[-which(set1GWAS$Prioritized == "nonprioritized"),]

set2GWAS <- named.deg[which(named.deg$BestID %in% Pooledset2),]
set2GWAS <-
  set2GWAS[-which(set2GWAS$Prioritized == "nonprioritized"),]

# Identifying GWAS Reference for Intersecting Genes
set1GWAS$Jansen <- set1GWAS$BestID %in% Jansenset1
set1GWAS$Kunkle <- set1GWAS$BestID %in% Kunkleset1
set1GWAS$Wightman <- set1GWAS$BestID %in% Wightmanset1

set2GWAS$Jansen <- set2GWAS$BestID %in% Jansenset2
set2GWAS$Kunkle <- set2GWAS$BestID %in% Kunkleset2
set2GWAS$Wightman <- set2GWAS$BestID %in% Wightmanset2
```

Responding to Reviewers

Comparing logRoR to “logFC alternative”

```r
# Calculating "FC-alternative" FC values and prioritization scores
total.deg <- total.deg %>%
  mutate(logFC.PFCminusCB = logFC.PFC - logFC.CB,
         PrioritizationAlt = rank.PFC / rank(-abs(logFC.PFCminusCB)))

# Plotting FC values
FCalt <- lm(total.deg$logFC.PFCminusCB ~ total.deg$logFC.RoR)

ggplot(total.deg, aes(logFC.RoR, logFC.PFCminusCB)) +
  geom_point() +
  geom_smooth(method = "lm") +
  theme_classic() +
  labs(x = "logRoR", y = "logPFC-logCB", title = 'FC Metric Comparison') +
  theme(plot.title = element_text(hjust = 0.5)) +
  annotate(
    geom = "text",
    x = -1.5,
    y = 1,
    label = paste(
      "y =",
      signif(FCalt$coefficients[2], 3),
      "x +",
      signif(FCalt$coefficients[1], 3)
    )
  ) +
  annotate(
    geom = "text",
    label = paste("r^2 = ", signif(summary(FCalt)$r.squared, 3)),
    x = -1.5,
    y = 0.8
  )
```

```
## `geom_smooth()` using formula = 'y ~ x'
```

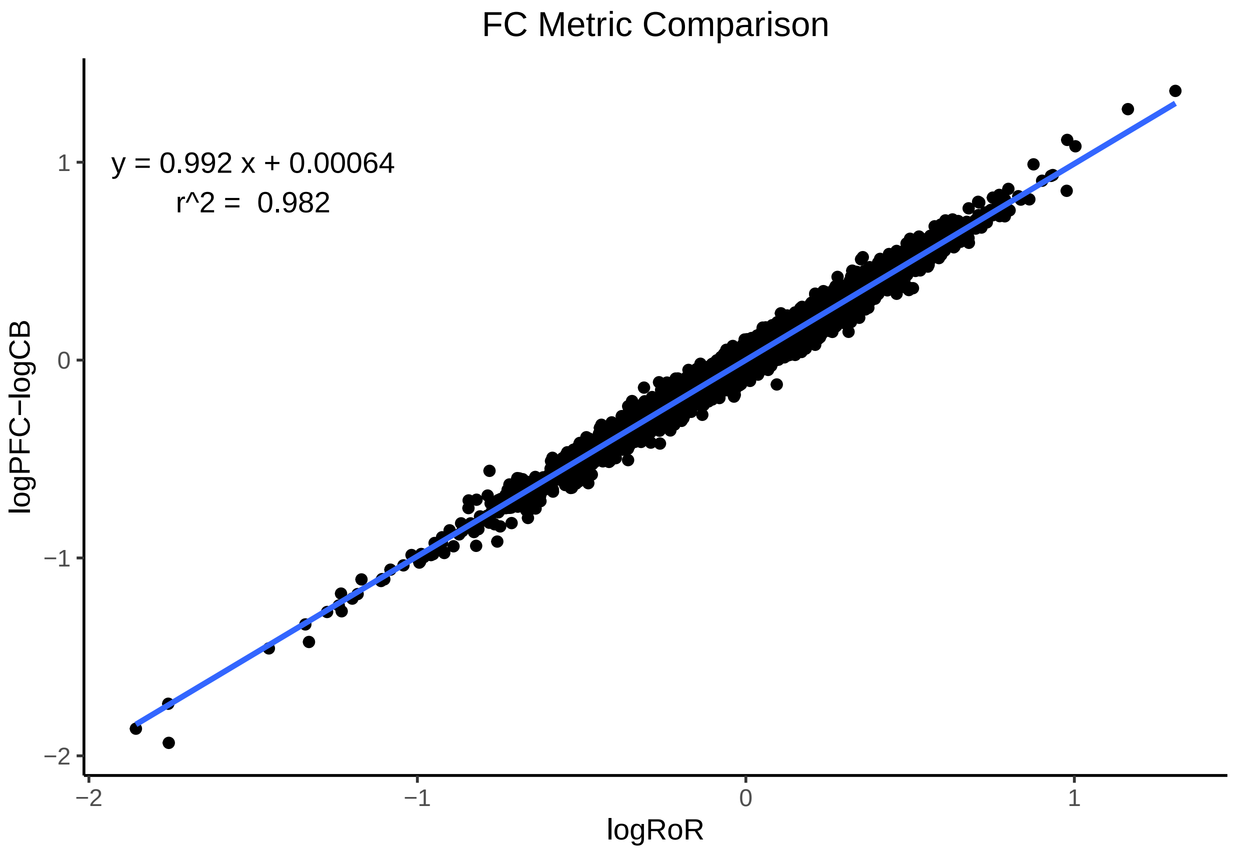


```
# Plotting prioritization scores
PrioritizationAlt <-
  lm(total.deg$PrioritizationAlt ~ total.deg$Prioritization)

ggplot(total.deg, aes(Prioritization, PrioritizationAlt)) +
  geom_point() +
  geom_smooth(method = "lm") +
  theme_classic() +
  scale_x_log10() +
  scale_y_log10() +
  labs(x = "logRoR Prioritization Score",
       y = "logPFC-logCB Prioritization Score",
       title = 'Prioritization Score Comparison') +
  theme(plot.title = element_text(hjust = 0.5)) +
  annotate(
    geom = "text",
    x = 0.01,
    y = 100,
    label = paste(
      "y =",
      signif(PrioritizationAlt$coefficients[2], 3),
      "x +",
      signif(PrioritizationAlt$coefficients[1], 3)
    )
```

```
  ) +
  annotate(
    geom = "text",
    label = paste("r^2 = ", signif(summary(PrioritizationAlt)$r.squared, 3)),
    x = 0.01,
    y = 50
  )
```

```
## `geom_smooth()` using formula = 'y ~ x'
```

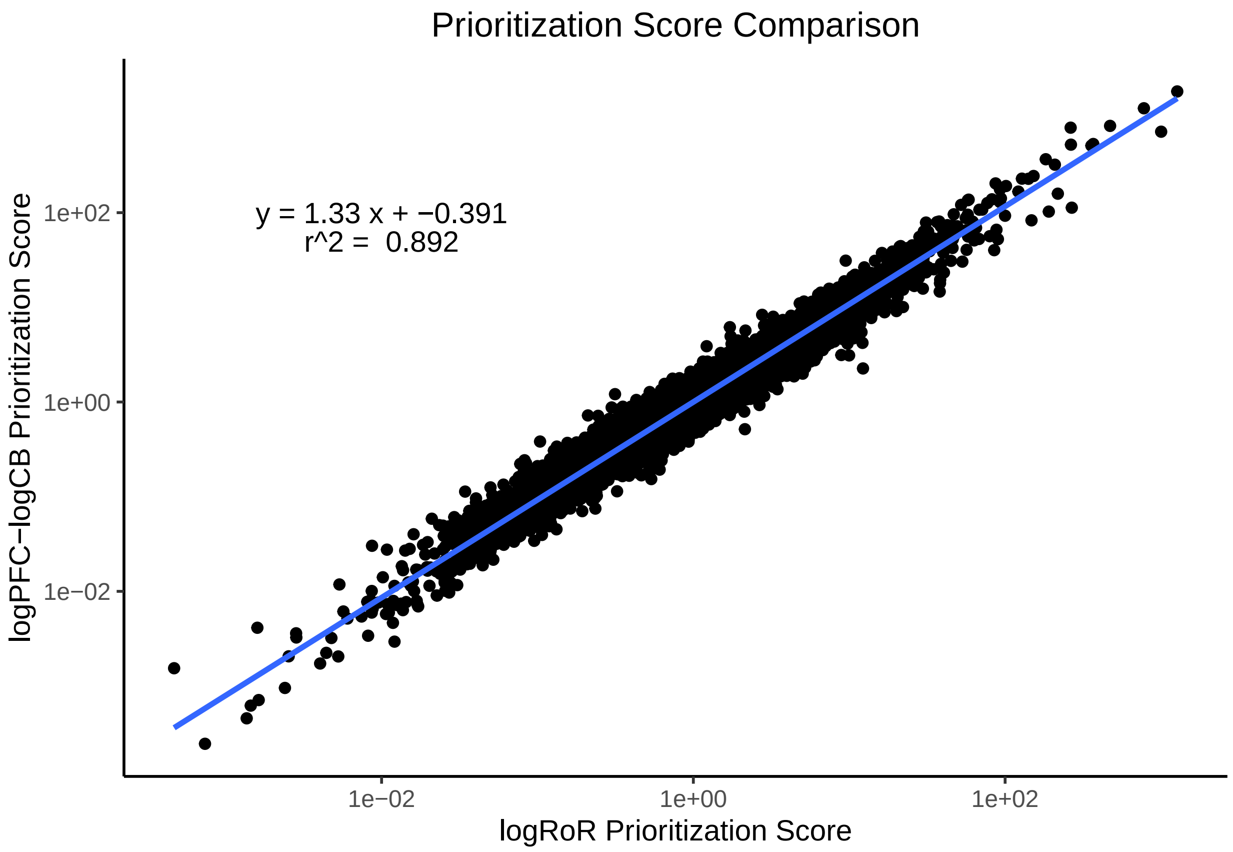

Distribution of Braak-correlated genes on Prioritization Curve

```
named.deg <-
  merge(named.deg,
        total.deg[which(!is.na(total.deg$BestID)),
                  c("ID", "logFC.PFCminusCB", "PrioritizationAlt")], by = "ID")

named.deg <- named.deg %>%
  mutate(
    BraakCorr = case_when(
      BestID %in% ZhangBraak$CB & !BestID %in% ZhangBraak$PFC ~ "CB",
      BestID %in% ZhangBraak$PFC &
        !BestID %in% ZhangBraak$CB ~ "PFC",
      BestID %in% ZhangBraak$CB &
        BestID %in% ZhangBraak$PFC ~ "Both",
```

```
    TRUE ~ "Non-correlated"
  )
) %>%
mutate(BraakCorr = factor(BraakCorr, levels = c("Non-correlated", "PFC", "CB",
↪  "Both"))) %>%
group_by(BraakCorr != "Non-correlated")
```

```
# Reviewer 2 variant 1
ggplot(
  named.deg %>% arrange(Prioritization, .by_group = TRUE),
  aes(
    x = Prioritization,
    y = rank(Prioritization),
    color = BraakCorr,
    alpha = BraakCorr
  )
) +
  geom_jitter(width = 0.14, height = 700) +
  theme_classic() +
  scale_x_log10() +
  labs(x = "log(Desirability Score)",
       y = "Desirability Rank",
       title = 'Prioritization by RoR Desirability') +
  theme(plot.title = element_text(hjust = 0.5),
        legend.position = c(0.85, 0.15)) +
  scale_color_manual(
    values = c(
      'PFC' = '#00CCFF',
      'CB' = '#FF0000',
      'Both' = 'blueviolet',
      'Non-correlated' = 'black'
    )
  ) +
  scale_alpha_manual(values = c(1, 1, 1, 1)) +
  geom_vline(xintercept = c(0.1126, 8.0883))
```

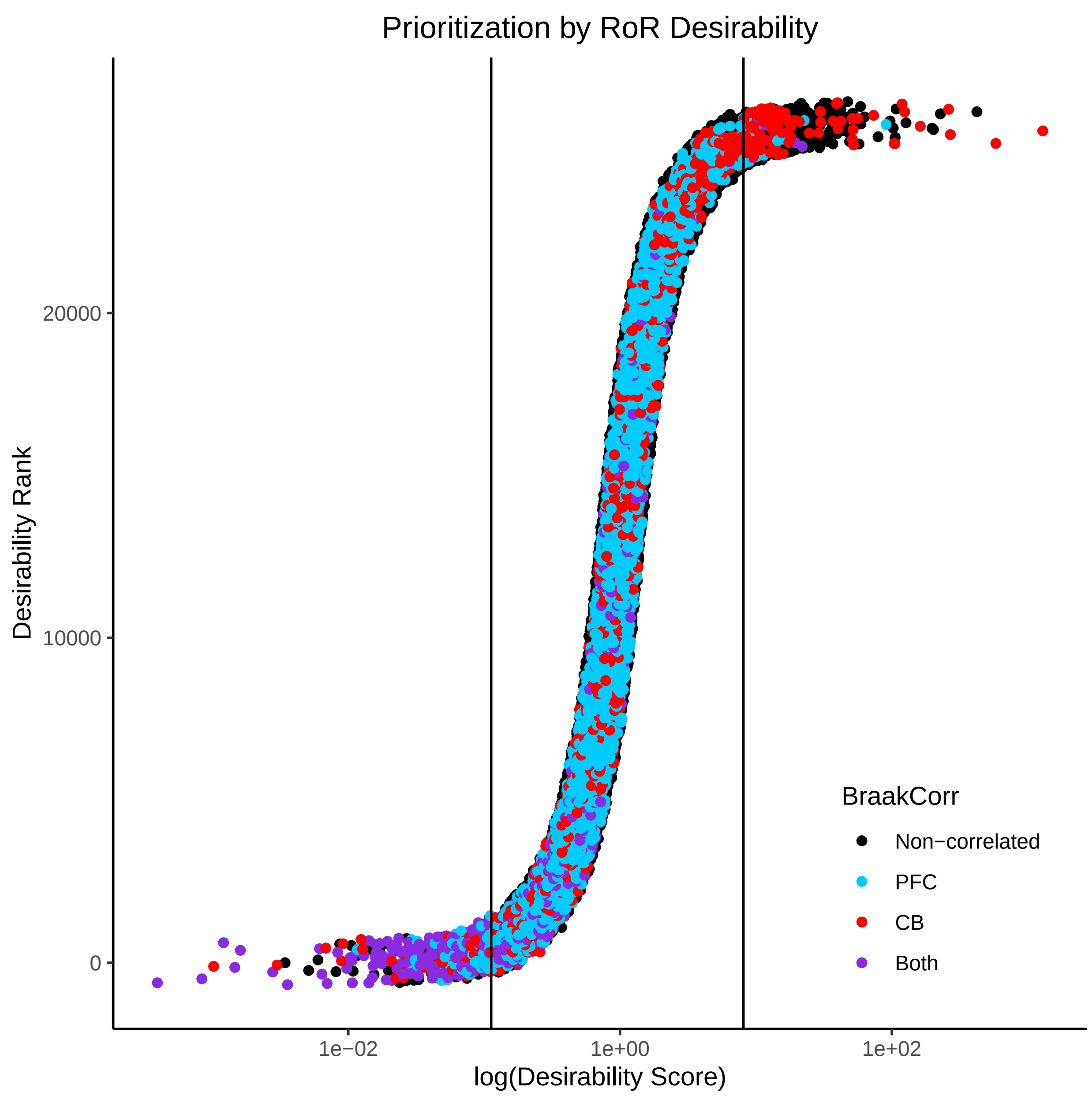


```
# Reviewer 2 variant 2
ggplot(
  data = named.deg %>% arrange(PrioritizationAlt, .by_group = TRUE),
  aes(
    x = PrioritizationAlt,
    y = rank(PrioritizationAlt),
    color = BraakCorr,
    alpha = BraakCorr
  )
) +
  geom_jitter(width = 0.14, height = 700) +
  theme_classic() +
  scale_x_log10() +
  labs(x = "log(Desirability Score)",
```

```r
    y = "Desirability Rank",
    title = 'Prioritization by logPFC-logCB Desirability') +
theme(plot.title = element_text(hjust = 0.5),
      legend.position = c(0.85, 0.15)) +
scale_color_manual(
  values = c(
    'PFC' = '#00CCFF',
    'CB' = '#FF0000',
    'Both' = 'blueviolet',
    'Non-correlated' = 'black'
  )
) +
scale_alpha_manual(values = c(1, 1, 1, 1)) +
geom_vline(xintercept = c(0.0961, 9.46))
```

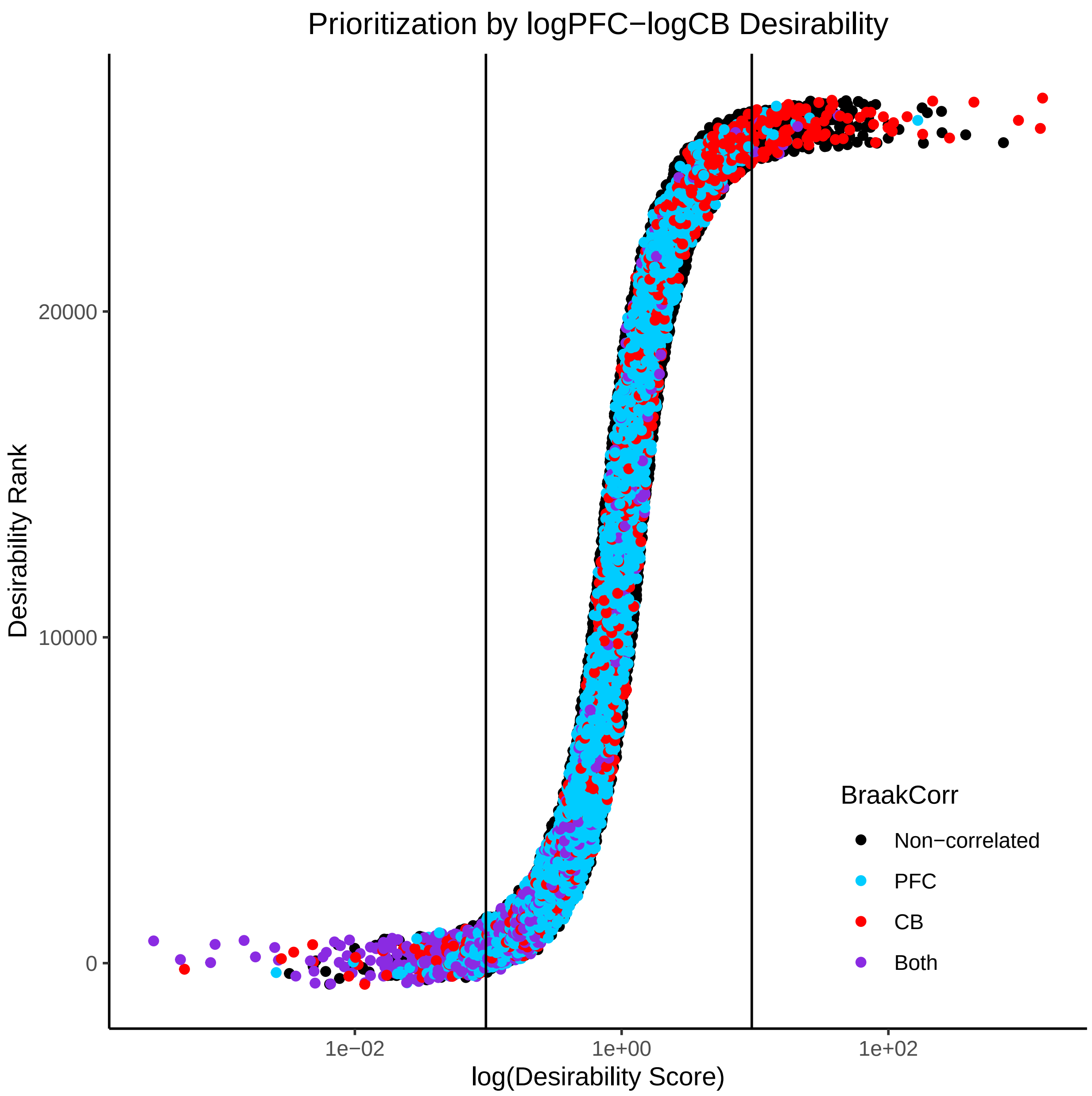


```
# Reviewer 2 variant 3
ggplot(
  data = named.deg %>% arrange(logFC.PFCminusCB, .by_group = TRUE),
  aes(
    x = logFC.PFCminusCB,
    y = rank(logFC.PFCminusCB),
    color = BraakCorr,
    alpha = BraakCorr
  )
) +
  geom_jitter(width = 0.07, height = 700) +
  theme_classic() +
  labs(x = "logRoR",
       y = "logRoR Rank",
```

```
     title = 'Prioritization by logPFC-logCB directly') +
theme(plot.title = element_text(hjust = 0.5),
      legend.position = c(0.85, 0.15)) +
scale_color_manual(
  values = c(
    'PFC' = '#00CCFF',
    'CB' = '#FF0000',
    'Both' = 'blueviolet',
    'Non-correlated' = 'black'
  )
) +
scale_alpha_manual(values = c(1, 1, 1, 1)) +
geom_vline(xintercept = c(-0.442 , 0.3931))
```

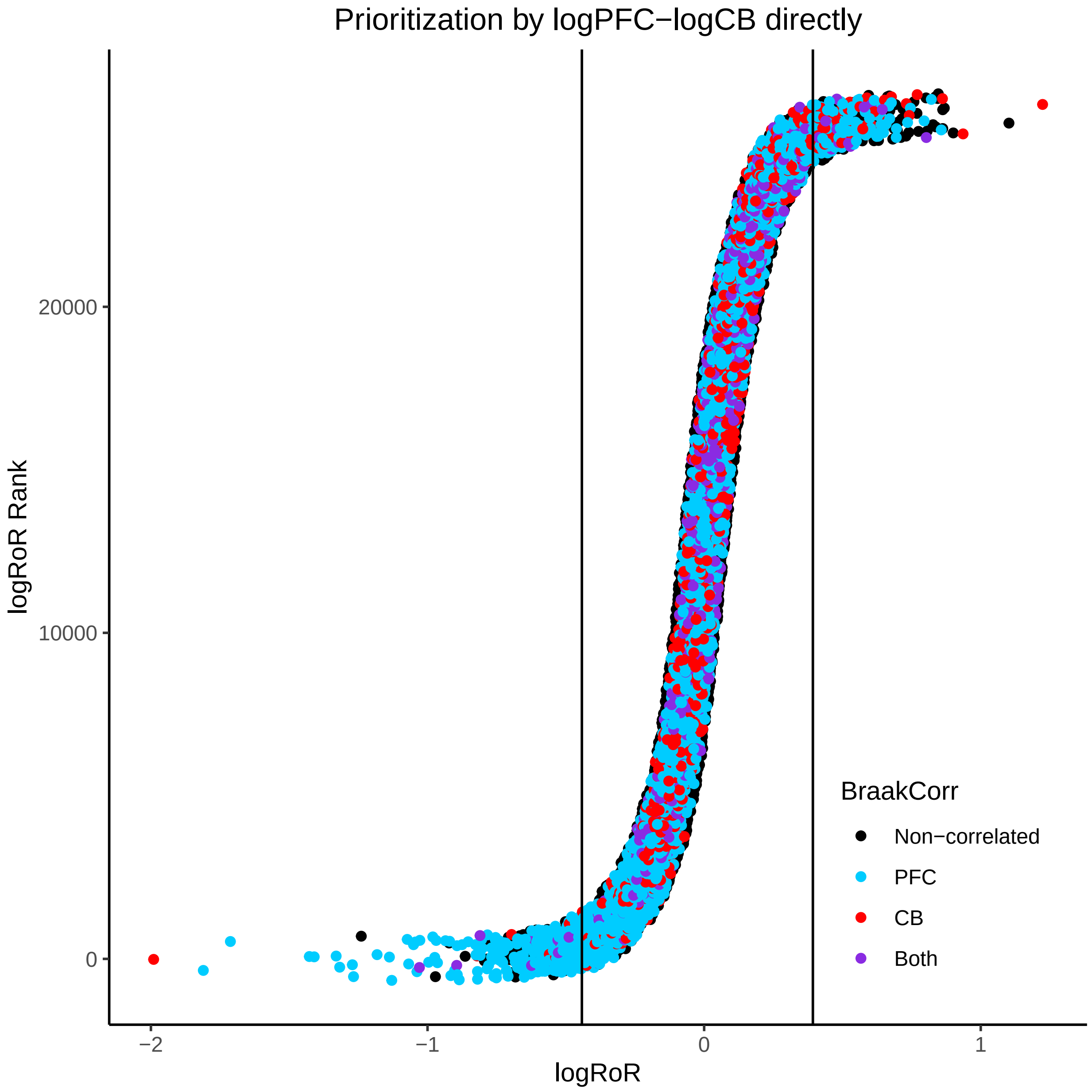

```r
# Reviewer 2 variant 4
ggplot(
  data = named.deg %>% arrange(logFC.RoR, .by_group = TRUE),
  aes(
    x = logFC.RoR,
    y = rank(logFC.RoR),
    color = BraakCorr,
    alpha = BraakCorr
  )
) +
  geom_jitter(width = 0.07, height = 700) +
  theme_classic() +
  labs(x = "logRoR",
       y = "logRoR Rank",
       title = 'Prioritization by RoR directly') +
  theme(plot.title = element_text(hjust = 0.5),
        legend.position = c(0.85, 0.15)) +
  scale_color_manual(
    values = c(
      'PFC' = '#00CCFF',
      'CB' = '#FF0000',
      'Both' = 'blueviolet',
      'Non-correlated' = 'black'
    )
  ) +
  scale_alpha_manual(values = c(1, 1, 1, 1)) +
  geom_vline(xintercept = c(-0.444, 0.3935))
```

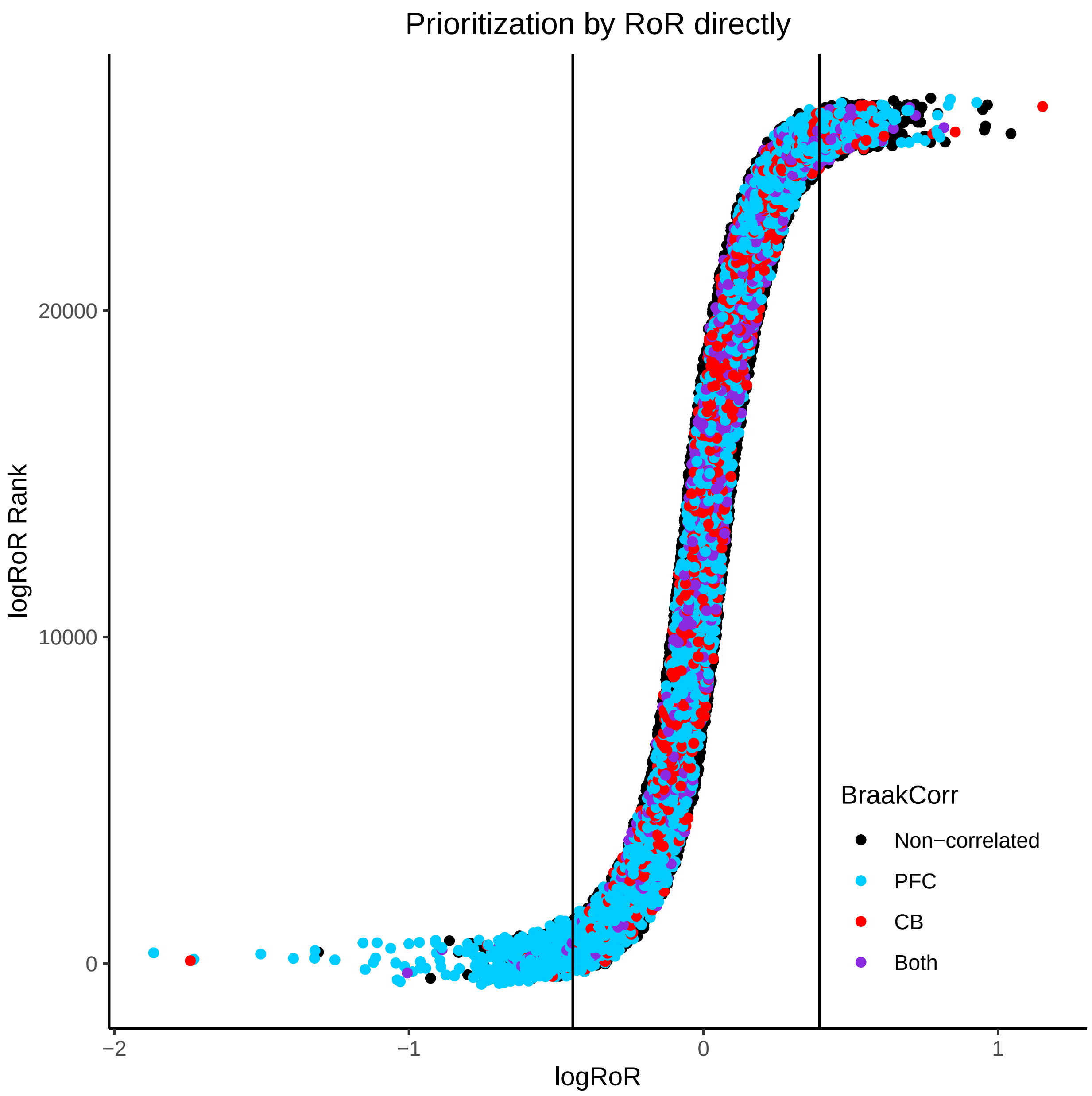

How does desirability sort?

Comparison to MayoSeq Dataset

```
namedMayo <-
  read_xlsx("S:/CAA/Gene Expression/Mayo RNAseq Analysis/R/221115_namedMayo.xlsx")

namedMayo <- namedMayo %>%
  group_by(Gene) %>%
  filter(adj.P.Val.RoR == min(adj.P.Val.RoR)) %>%
  setNames(paste0("MAYO", names(.)))

namedGSE <- named.deg %>%
```

```r
  group_by(BestID) %>%
  filter(adj.P.Val.RoR == min(adj.P.Val.RoR)) %>%
  setNames(paste0("GSE", names(.)))

shared <-
  merge(namedGSE, namedMayo, by.x = "GSEBestID", by.y = "MAYOGene")

ggcor <- function(data, x, y) {
  # Calculate best-fit line
  lm <- lm(data[, y] ~ data[, x])
  eq <-
    paste("y =",
          signif(lm$coefficients[2], 3),
          "x +",
          signif(lm$coefficients[1], 3))

  # Calculate correlation statistics
  test <- cor.test(x = data[, x], y = data[, y], method = "pearson")
  r <- paste("r = ", signif(unname(test$estimate), 3))
  p <- paste("p = ", signif(test$p.value, 3))

  # Plot annotated scatterplot
  ggplot(data, aes_string(x = x, y = y)) +
    geom_point() +
    geom_smooth(method = "lm") +
    #ggpubr::stat_cor(method = "pearson", exact = "TRUE") +
    annotate(
      geom = "text",
      label = eq,
      x = min(data[, x]),
      y = max(data[, y]),
      vjust = "inward",
      hjust = "inward"
    ) +
    annotate(
      geom = "text",
      label = r,
      x = min(data[, x]),
      y = max(data[, y]) - 0.2,
      vjust = "inward",
      hjust = "inward"
    ) +
    annotate(
      geom = "text",
      label = p,
      x = min(data[, x]),
      y = max(data[, y]) - 0.4,
      vjust = "inward",
      hjust = "inward"
    ) +
    theme_classic() +
    theme(plot.title = element_text(hjust = 0.5))
}
```

```r
ggcor(shared, "GSElogFC.RoR", "MAYOlogFC.RoR")
```

```
## `geom_smooth()` using formula = 'y ~ x'
```

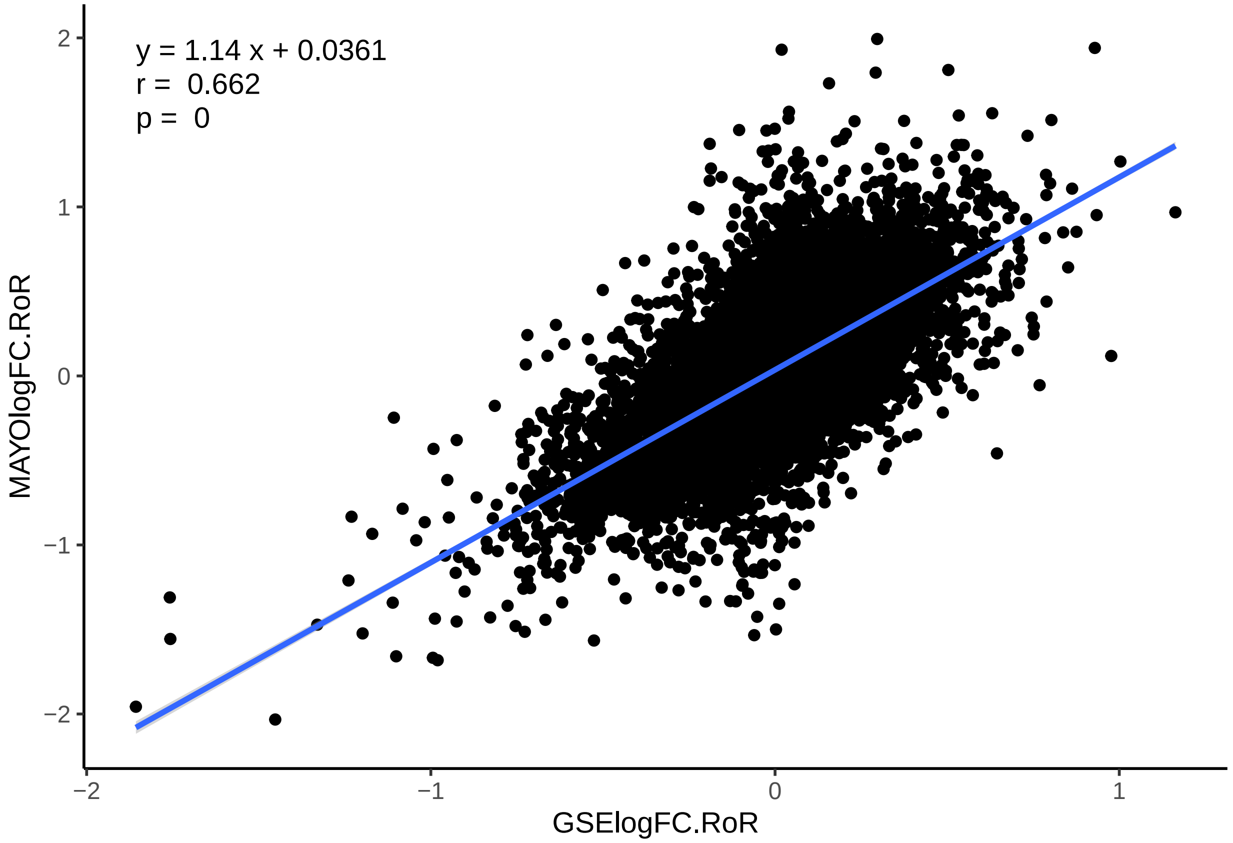


```r
ggcor(shared, "GSElogFC.PFC", "MAYOlogFC.TCX")
```

```
## `geom_smooth()` using formula = 'y ~ x'
```

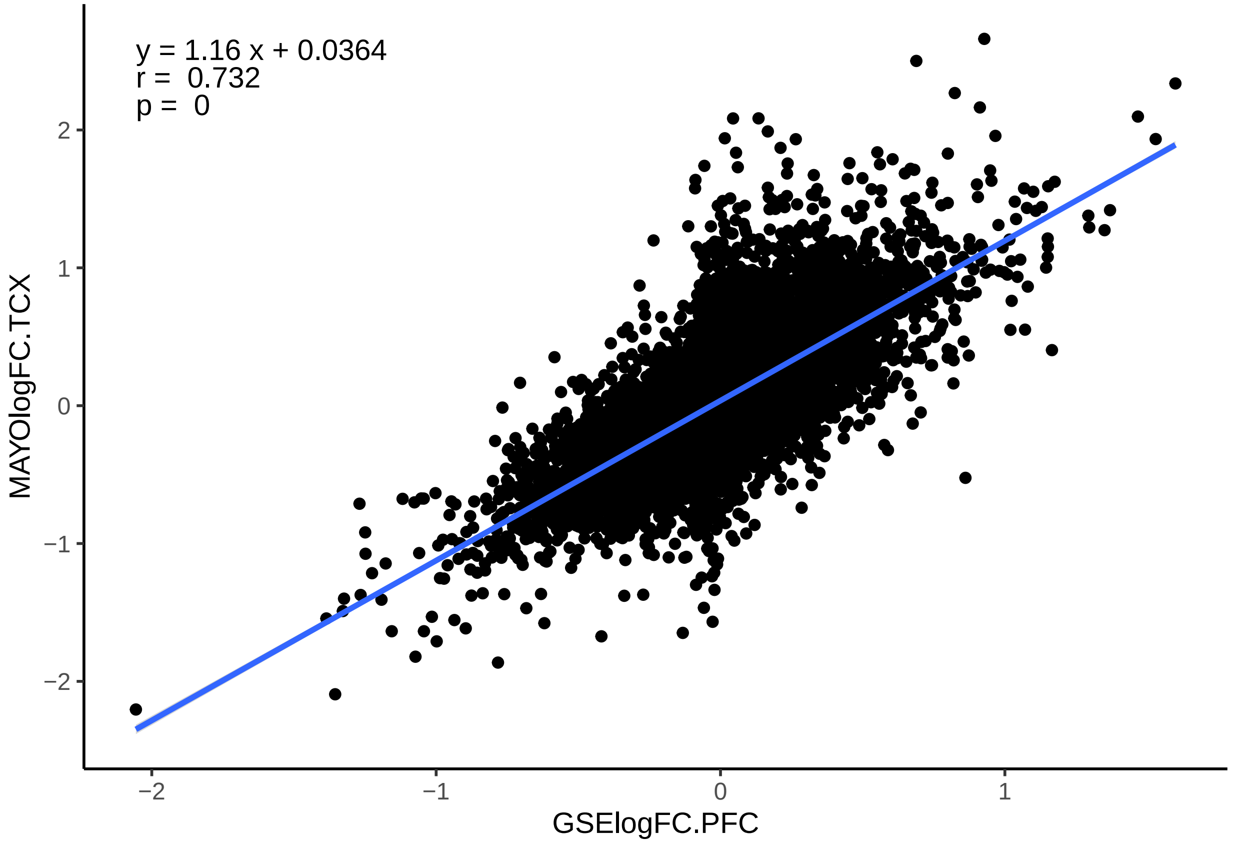


```
ggcor(shared, "GSElogFC.CB", "MAYOlogFC.CB")
```

```
## `geom_smooth()` using formula = 'y ~ x'
```

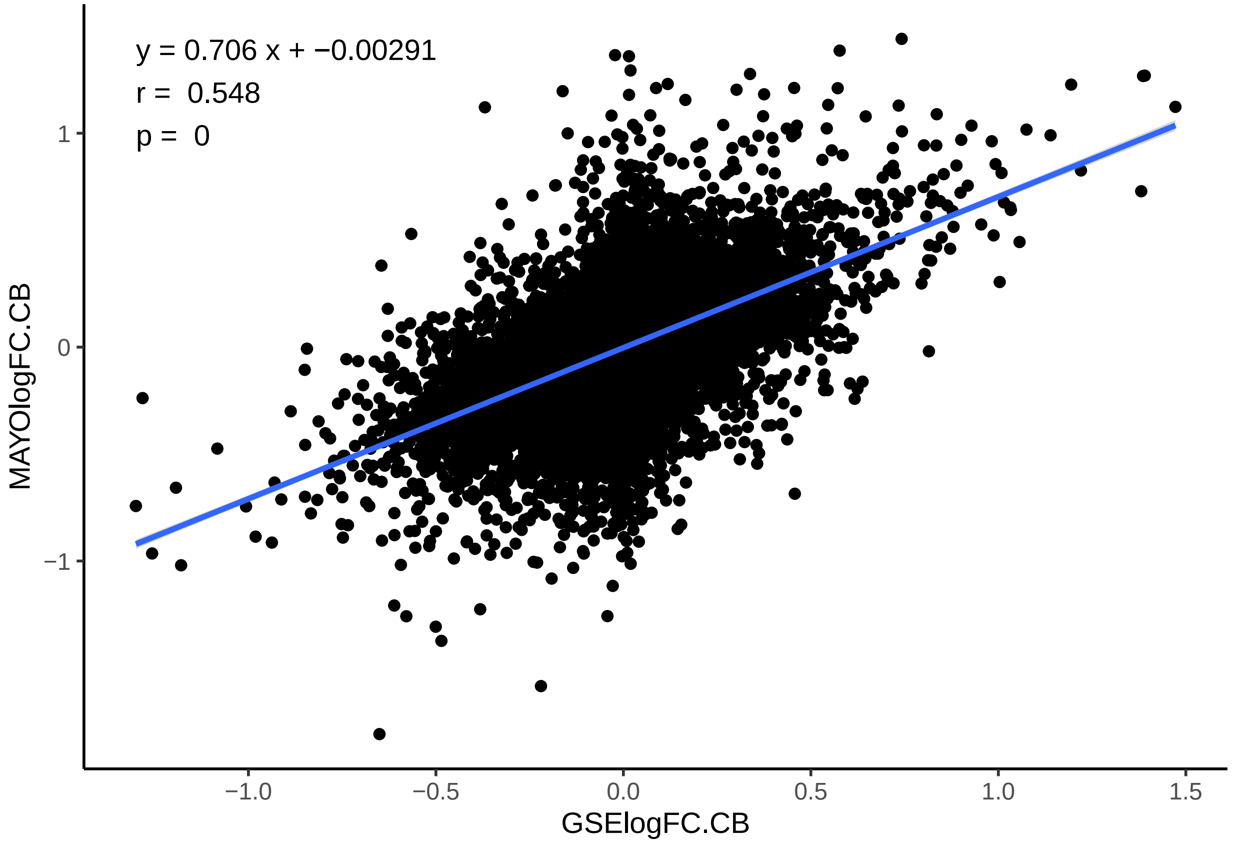


```
ggcor(
  shared %>% filter(GSEadj.P.Val.RoR < 0.05 &
                    MAYOadj.P.Val.RoR < 0.05),
  "GSElogFC.RoR",
  "MAYOlogFC.RoR"
)
```

```
## `geom_smooth()` using formula = 'y ~ x'
```

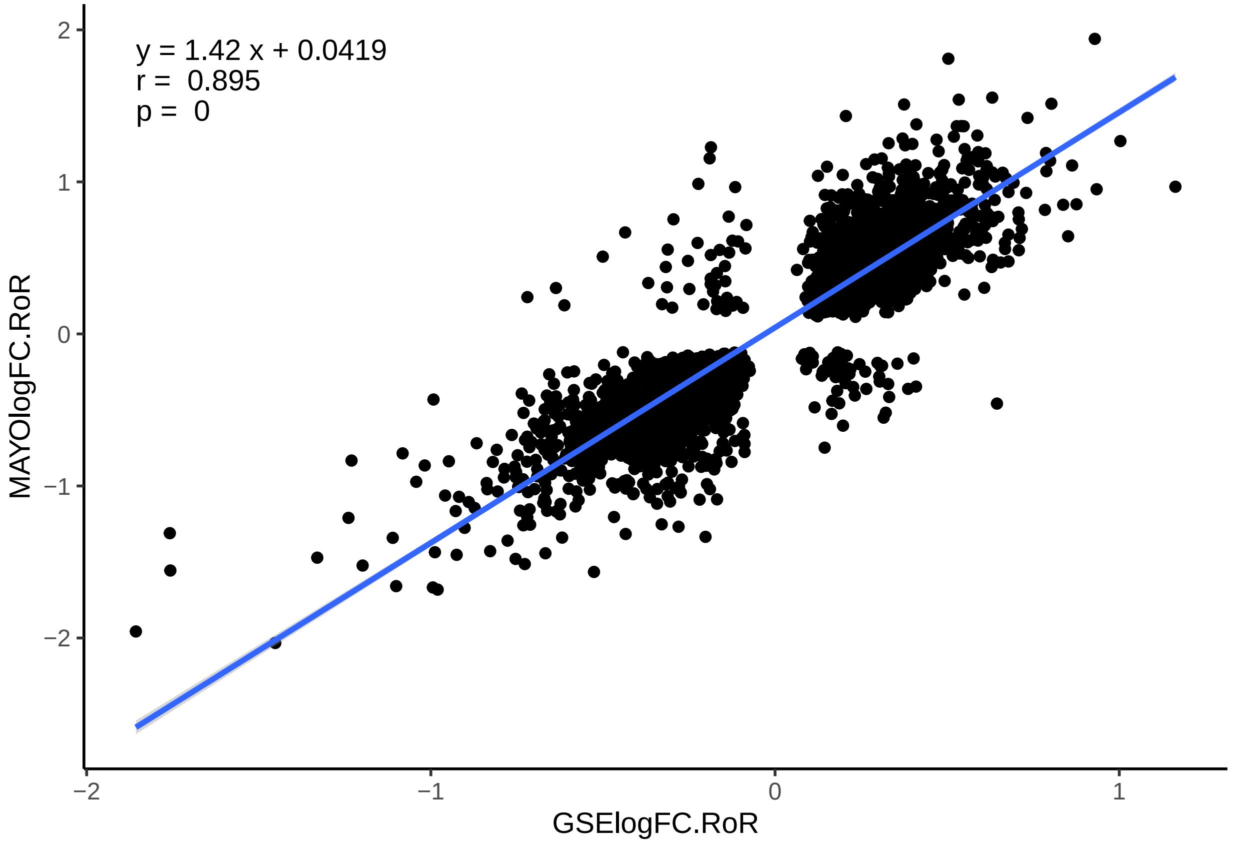


```
ggcor(
  shared %>% filter(GSEadj.P.Val.PFC < 0.05 &
                    MAYOadj.P.Val.TCX < 0.05),
  "GSElogFC.PFC",
  "MAYOlogFC.TCX"
)
```

```
## `geom_smooth()` using formula = 'y ~ x'
```

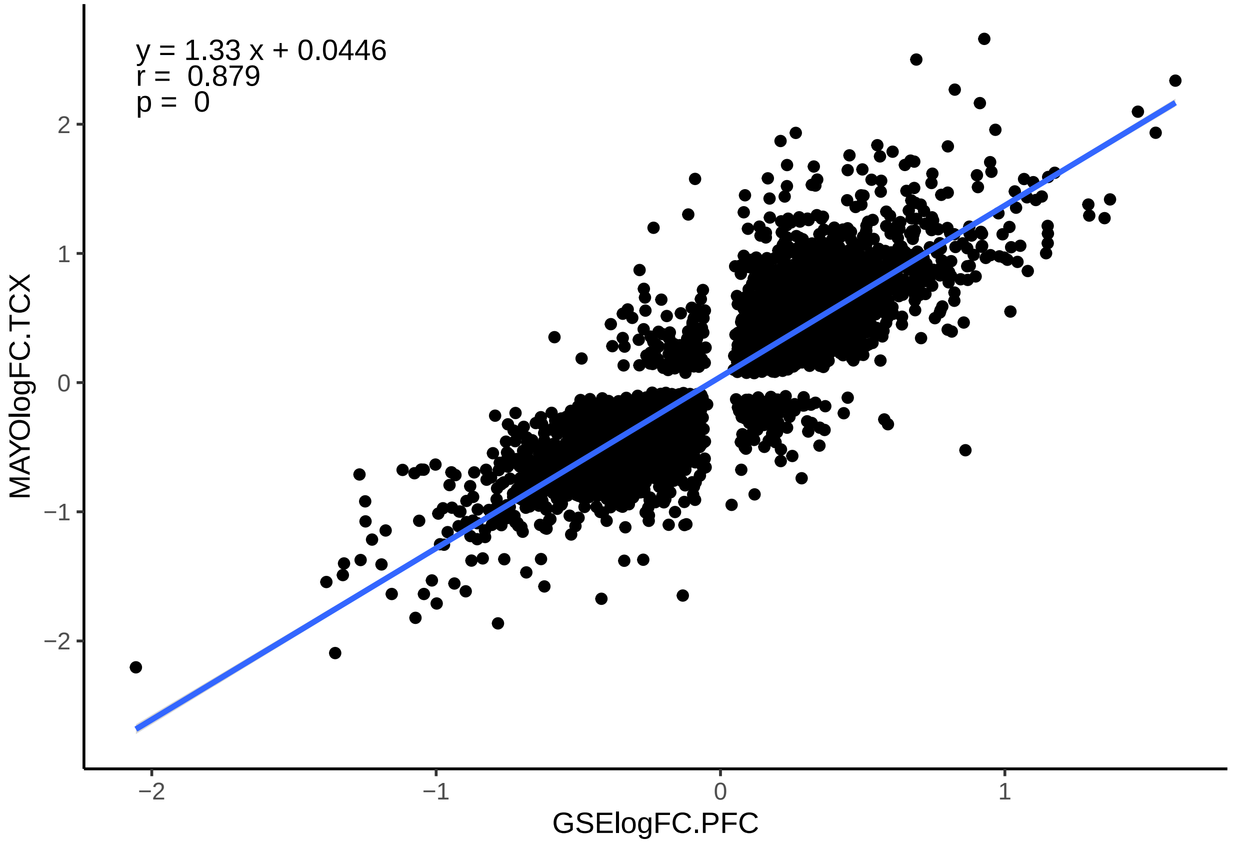


```
ggcor(
  shared %>% filter(GSEadj.P.Val.CB < 0.05 &
                    MAYOadj.P.Val.CB < 0.05),
  "GSElogFC.CB",
  "MAYOlogFC.CB"
)
```

```
## `geom_smooth()` using formula = 'y ~ x'
```

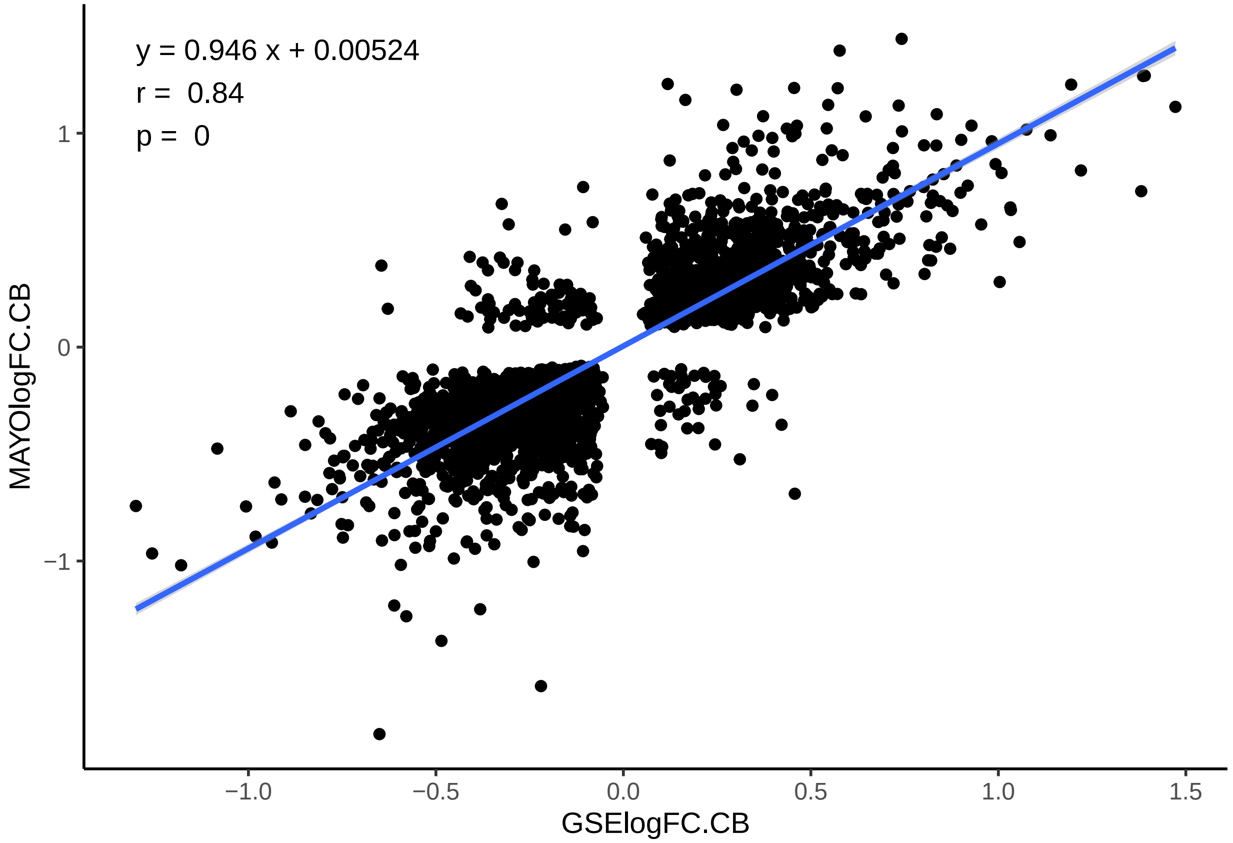

Session information for reproducibility

```
sessionInfo()
```

```
## R version 4.1.0 (2021-05-18)
## Platform: x86_64-w64-mingw32/x64 (64-bit)
## Running under: Windows 10 x64 (build 19045)
##
## Matrix products: default
##
## locale:
## [1] LC_COLLATE=English_United States.1252
## [2] LC_CTYPE=English_United States.1252
## [3] LC_MONETARY=English_United States.1252
## [4] LC_NUMERIC=C
## [5] LC_TIME=English_United States.1252
##
## attached base packages:
## [1] stats4    parallel  stats     graphics  grDevices utils     datasets
## [8] methods   base
##
## other attached packages:
##  [1] knitr_1.42
##  [2] writexl_1.4.2
##  [3] readxl_1.4.2
```

```
##  [4] GeneOverlap_1.28.0
##  [5] ggplot2_3.4.1
##  [6] mygene_1.28.0
##  [7] GenomicFeatures_1.44.2
##  [8] AnnotationDbi_1.54.1
##  [9] GenomicRanges_1.44.0
## [10] GenomeInfoDb_1.28.4
## [11] IRanges_2.26.0
## [12] S4Vectors_0.30.2
## [13] tidyr_1.3.0
## [14] ExpressionNormalizationWorkflow_1.18.0
## [15] report_0.5.6
## [16] R.utils_2.12.2
## [17] R.oo_1.25.0
## [18] R.methodsS3_1.8.2
## [19] edgeR_3.34.1
## [20] limma_3.48.3
## [21] genefilter_1.74.1
## [22] tibble_3.1.8
## [23] dplyr_1.1.0
## [24] GEOquery_2.60.0
## [25] Biobase_2.52.0
## [26] BiocGenerics_0.38.0
##
## loaded via a namespace (and not attached):
##   [1] backports_1.4.1            Hmisc_4.8-0
##   [3] BiocFileCache_2.0.0        plyr_1.8.8
##   [5] splines_4.1.0              BiocParallel_1.26.2
##   [7] sva_3.40.0                 digest_0.6.31
##   [9] htmltools_0.5.4            fansi_1.0.4
##  [11] magrittr_2.0.3             checkmate_2.1.0
##  [13] memoise_2.0.1              cluster_2.1.4
##  [15] tzdb_0.3.0                 Biostrings_2.60.2
##  [17] readr_2.1.4                annotate_1.70.0
##  [19] matrixStats_0.63.0         vroom_1.6.1
##  [21] prettyunits_1.1.1          jpeg_0.1-10
##  [23] colorspace_2.1-0           blob_1.2.3
##  [25] rappdirs_0.3.3             xfun_0.37
##  [27] crayon_1.5.2               RCurl_1.98-1.10
##  [29] jsonlite_1.8.4             snm_1.40.0
##  [31] lme4_1.1-31                survival_3.5-3
##  [33] glue_1.6.2                 gtable_0.3.1
##  [35] zlibbioc_1.38.0            XVector_0.32.0
##  [37] DelayedArray_0.18.0        scales_1.2.1
##  [39] vsn_3.60.0                 DBI_1.1.3
##  [41] Rcpp_1.0.10                xtable_1.8-4
##  [43] progress_1.2.2             htmlTable_2.4.1
##  [45] foreign_0.8-84             bit_4.0.5
##  [47] preprocessCore_1.54.0      sqldf_0.4-11
##  [49] Formula_1.2-5              htmlwidgets_1.6.1
##  [51] httr_1.4.5                 gplots_3.1.3
##  [53] RColorBrewer_1.1-3         ellipsis_0.3.2
##  [55] farver_2.1.1               pkgconfig_2.0.3
##  [57] XML_3.99-0.13              nnet_7.3-18
```

```
##  [59] dbplyr_2.3.1                deldir_1.0-6
##  [61] locfit_1.5-9.7              utf8_1.2.3
##  [63] labeling_0.4.2              tidyselect_1.2.0
##  [65] rlang_1.0.6                 cellranger_1.1.0
##  [67] munsell_0.5.0               tools_4.1.0
##  [69] cachem_1.0.7                cli_3.4.1
##  [71] gsubfn_0.7                  generics_0.1.3
##  [73] RSQLite_2.3.0               evaluate_0.20
##  [75] stringr_1.5.0               fastmap_1.1.0
##  [77] yaml_2.3.7                  bit64_4.0.5
##  [79] caTools_1.18.2              purrr_1.0.1
##  [81] KEGGREST_1.32.0             nlme_3.1-162
##  [83] xml2_1.3.3                  biomaRt_2.48.3
##  [85] compiler_4.1.0              rstudioapi_0.14
##  [87] filelock_1.0.2              curl_5.0.0
##  [89] png_0.1-8                   affyio_1.62.0
##  [91] stringi_1.7.12              highr_0.10
##  [93] lattice_0.20-45             Matrix_1.5-3
##  [95] nloptr_2.0.3                vctrs_0.5.2
##  [97] pillar_1.8.1                lifecycle_1.0.3
##  [99] BiocManager_1.30.20         pvca_1.32.0
## [101] data.table_1.14.8           bitops_1.0-7
## [103] insight_0.19.0              corpcor_1.6.10
## [105] rtracklayer_1.52.1          R6_2.5.1
## [107] BiocIO_1.2.0                latticeExtra_0.6-30
## [109] affy_1.70.0                 KernSmooth_2.23-20
## [111] gridExtra_2.3               gtools_3.9.4
## [113] boot_1.3-28.1               MASS_7.3-58.2
## [115] chron_2.3-59                proto_1.0.0
## [117] SummarizedExperiment_1.22.0 rjson_0.2.21
## [119] withr_2.5.0                 GenomicAlignments_1.28.0
## [121] Rsamtools_2.8.0             GenomeInfoDbData_1.2.6
## [123] mgcv_1.8-41                 hms_1.1.2
## [125] grid_4.1.0                  rpart_4.1.19
## [127] minqa_1.2.5                 rmarkdown_2.20
## [129] MatrixGenerics_1.4.3        base64enc_0.1-3
## [131] rematch_1.0.1               interp_1.1-3
## [133] restfulr_0.0.15
```
