# Supplementary figures and images for: Identification of gene networks mediating regional resistance to tauopathy in late-onset Alzheimer’s disease
Source: PLoS Genet. 2023 Mar 27;19(3):e1010681. doi: 10.1371/journal.pgen.1010681 (PMC10079065; doi:10.1371/journal.pgen.1010681)

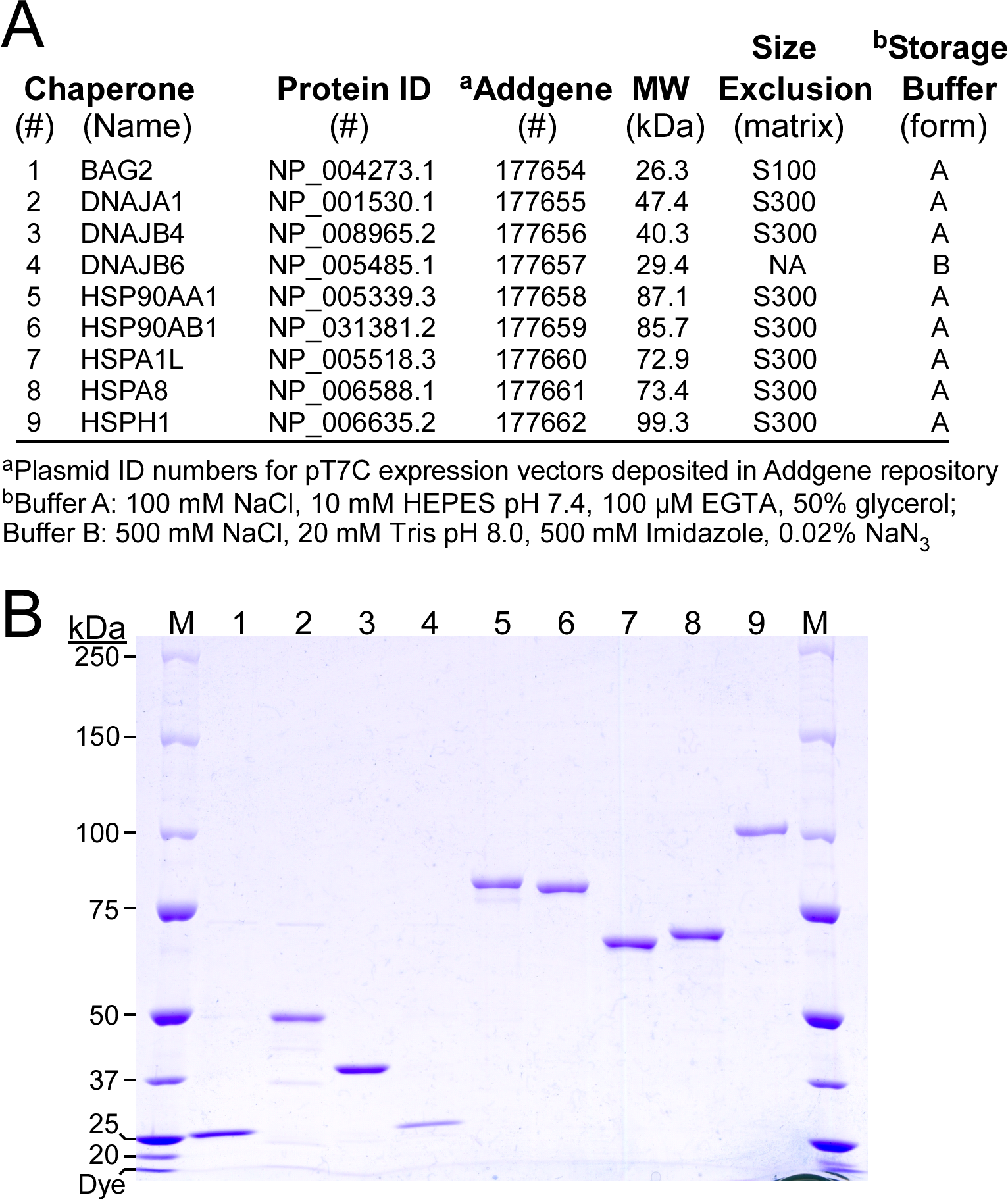

Supplement: S1 Fig — (A) Nine molecular chaperones identified in contrasting Gene Set 1 cloned from a CB cDNA library, expressed in E. coli, and purified by column chromatography. (B) SDS-polyacrylamide gel electrophoresis (6–12% acrylamide gradient) of purified chaperones listed in Panel A. Purified proteins were used in tau aggregation assays. (TIF) [file pgen.1010681.s003.tif]
